# Organic Materials for Biohybrid Photocatalysis and Photoelectrochemical Devices

**DOI:** 10.1021/acs.chemrev.5c00946

**Published:** 2026-05-15

**Authors:** Mariia V. Pavliuk, Bin Cai, Larissa Kurth, Reiner Sebastian Sprick, Gustav Berggren, Haining Tian

**Affiliations:** a Department of Chemistry-Ångström Laboratory, 8097 Uppsala University, Box 523, Uppsala SE 751 20, Sweden; b Institute for Energy Research, Jiangsu University, Zhenjiang 212013, China; c Department of Pure and Applied Chemistry, 12676 University of Strathclyde, Thomas Graham Building, 295 Cathedral Street, Glasgow, Scotland G1 1XL, United Kingdom

## Abstract

The integration of biological catalysts with organic light-harvesting materials represents a rapidly advancing strategy for sustainable solar-to-chemical energy conversion. Recent advances demonstrate that photoexcited organic materials can effectively activate redox enzymes and whole-cell systems to catalyze fuel-forming reactions such as H_2_ evolution and CO_2_ fixation as well as selective oxidations and chiral transformations under mild conditions. This review summarizes recent advances in semiartificial photosynthesis driven by organic materials and presents a systematic overview of the major classes involved. In particular, it focuses on a) organic photosensitizers including molecular organic photosensitizers, carbon dots, graphene, carbon nitride, and organic aggregation nanoparticles (e.g., polymer dots/Pdots, molecule nanoparticles) as well as b) organic redox mediators. These materials are evaluated in terms of their photophysical properties, compatibility with biocatalysts, and their roles in photoinduced charge generation, charge separation, and interfacial electron transfer between abiotic and biotic components. Emerging trends toward mediator-free and water-driven systems, challenges related to photostability, biocompatibility, as well as paired redox catalysis generating value-added products are also discussed. Additionally, the effects of the sacrificial electron donor on biohybrid performance and development of biohybrid photoelectrochemical catalysis have been evaluated. Finally, this review aims to highlight the key scientific and technological questions that must be addressed to advance the field toward efficient, scalable, and environmentally benign biohybrid photocatalytic and photoelectrochemical platforms.

## Graphical abstract

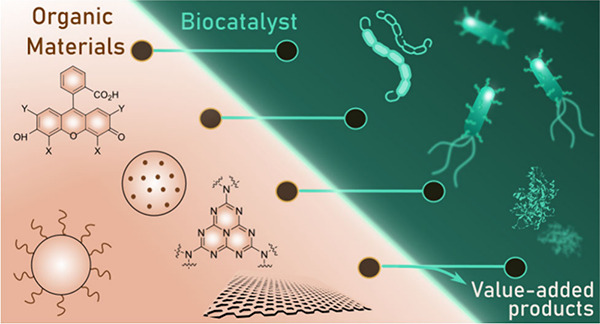

## 1 Introduction

Advanced catalytic technologies, particularly those that convert renewable resources into fuels and chemicals using solar energy, offer a transformative pathway to mitigate climate change, strengthen energy security, and enable a sustainable future.^1^ At the core of this approach is the synergy between materials that efficiently harvest solar energy and catalysts that perform redox transformations to yield target products with high selectivity under mild conditions. So-called artificial photosynthesis relies solely on synthetic materials, while natural photosynthesis depends on nature’s components, therefore semiartificial photosynthesis (also known as photobiocatalysis or biohybrid photocatalysis)^2–5^ represents an interdisciplinary approach that integrates synthetic photosensitizers with nature’s biocatalysts. This integration leverages the complementary strengths of abiotic and biotic components to achieve reactivities and efficiencies unattainable by either alone. It is distinct from biomimetic catalysis, which employs fully synthetic catalysts or materials designed to mimic the function of natural ones. In semiartificial photosynthesis, biocatalytic reactions are driven by photosensitizers, with biocatalysts either dispersed in solution or immobilized on electrodes in photocatalytic or photoelectrochemical (PEC) systems, respectively.

The growing interest in biocatalysts for producing fuels and high-value chemicals stems from their naturally evolved catalytic machinery, which guarantees high reaction selectivity and favorable reaction kinetics at generally lower overpotential.^6^ In addition, the turnover number (TON) and turnover frequency (TOF) of biocatalysts can be close to the diffusion limit. In biohybrid systems, enzymes offer exceptional selectivity and efficiency under mild conditions, subcellular organelles may enable light-driven compartmentalized processes, and whole-cell microorganisms perform complex, multistep bioconversions offering self-regeneration, together providing complementary strengths. Recent advancements in bioengineering have positioned biocatalysts as competitive alternatives to traditional synthetic molecular catalysts, thereby promoting their growing use in the chemical industry.^7–9^

Both organic and inorganic materials can be employed as light-harvesting components to drive the solar-to-bioproduct conversion reactions of biocatalysts.^6,10^ Organic photosensitizers (OPSs) are defined as metal-free materials that upon light irradiation generate electron–hole pairs or free charges, which can then be transferred to drive redox transformations. In contrast to conventional inorganic semiconducting materials, OPSs offer potential advantages, including excellent optical and electronic properties, good biocompatibility, and versatile surface functionality necessary to interact with a biocatalyst.^11^ The metal-free nature of OPSs largely reduces the dependence on harmful elements (e.g., Cd, Ru present in some inorganic materials such as quantum dots and metal oxides), making them especially suitable for integration into biohybrid systems.^5,12–17^ Structural modifications of the OPSs can effectively influence the band gap energy as well as the positions of the lowest unoccupied (LUMO) and highest occupied molecular orbitals (HOMO), thereby allowing tuning not only their photophysical properties, but also the charge transfer driving force. Figure 1 illustrates a simplified overview of the general distinctions between organic and inorganic light-harvesting materials used in biohybrid catalysis, while more comprehensive comparisons can be found in previously published reviews.^18,19^

**Figure 1:**
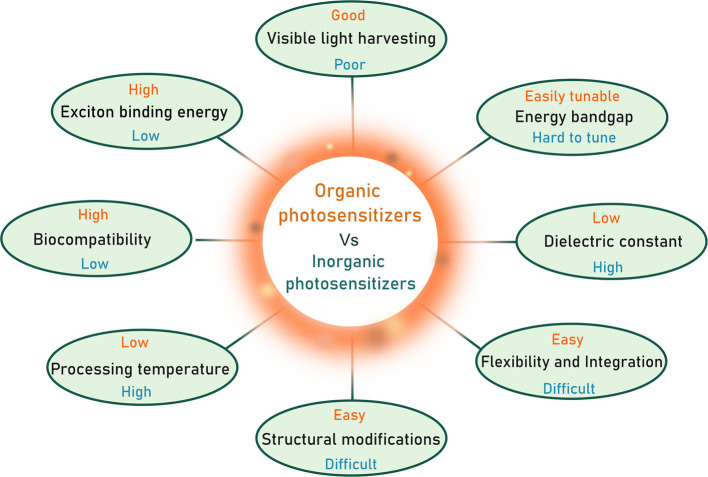
Schematic diagram of key advantages of organic photosensitizers over their inorganic counterparts.

Over the past two decades the field of organic materials-based semiartificial photosynthesis has experienced rapid and substantial growth with expanding applications in both reduction^5,20–34^ and oxidation reactions.^35–38^ In all cases, efficient charge transport from light harvesting center to catalytic center is essential for driving redox biocatalytic reactions, particularly for deeply buried active sites of biocatalysts.^39^ Despite these advances, the field still faces significant challenges, including the need (a) to better understand charge transfer mechanistic pathways within the biohybrid assemblies, particularly at the interface between an artificial photosensitizer and a biocatalyst,^40^ (b) to design organic photosensitizers with excellent light harvesting ability, while rendering long-lived charge-separated states for efficient charge transfer to the biocatalyst,^19^ (c) to improve system stability and scalability, (d) to optimize operational conditions and rationally engineer the biotic–abiotic interface to sustain productive coupling, while ensuring that the biocatalyst remains active despite operation under non-native conditions, and (e) to expand the reaction scope.

In light of these challenges, this review aims to highlight what must be addressed to advance the field of semiartificial photosynthesis based on organic materials including photosensitizers and redox mediators. Section 2 begins with a brief historical overview of organic biohybrid assemblies and their operating principles, followed by a discussion of the strengths and limitations of enzymatic- and cell-based systems. Section 3 discusses the fundamental requirements for light harvesting organic photosensitizers and surveys the main families of these photosensitizers previously integrated into the biohybrid systems. In Section 4 we examine the role of commonly used electronic coupling agents, such as redox mediators, used to wire organic photosensitizers and biocatalysts. Section 5 identifies the challenges associated with the impact of sacrificial reagents and presents alternatives that circumvent these issues. Section 6 focuses on the operational principles of biohybrid PEC systems and their components.^41^ Finally, Section 7 outlines the remaining challenges and proposes future research directions to enhance the performance, stability, and scalability of biohybrid systems based on organic materials for solar-to-fuel/chemical energy conversion. Additionally, this review focuses on organic-material-driven semiartificial photosynthesis, systematically positioning organic photosensitizers as the central functional components, establishing structure–function correlations with biocatalyst compatibility and interfacial charge transfer; and providing the first comprehensive analysis of photosensitizer regeneration and the limitations of sacrificial electron donors (SEDs) - areas not systematically addressed in prior biohybrid photocatalysis reviews.^42^

## 2 Biohybrid Components and Operational Principles

### 2.1 Historical Overview and Operational Principles

Although, one of the earliest examples of biohybrid systems using inorganic photosensitizers appeared as early as 1987, the first system based on organic photosensitizers was not reported until 2007.^23^ It took five more years until the first major breakthrough in the field, when Park’s group introduced self-assembled organic light-harvesting peptide nanotubes (FF-THPP, synthesized through the self-assembly of diphenylalanine (FF) and tetrahydroxyphenylporphyrin (THPP)) coupled with glutamate dehydrogenase (GDH) for the semiartificial photosynthesis of L-glutamate, followed by Reisner’s group that employed an Eosin Y:[NiFeSe]-hydrogenase assembly for hydrogen evolution.^27,43^ Since then, a wide variety of organic light harvesters, also commonly called photosensitizers, have been developed for photobiocatalytic applications, including carbon dots, polymer nanoparticles/polymer dots, molecule nanoparticles, carbon nitride materials, and others (Figure 2).^44^

**Figure 2:**
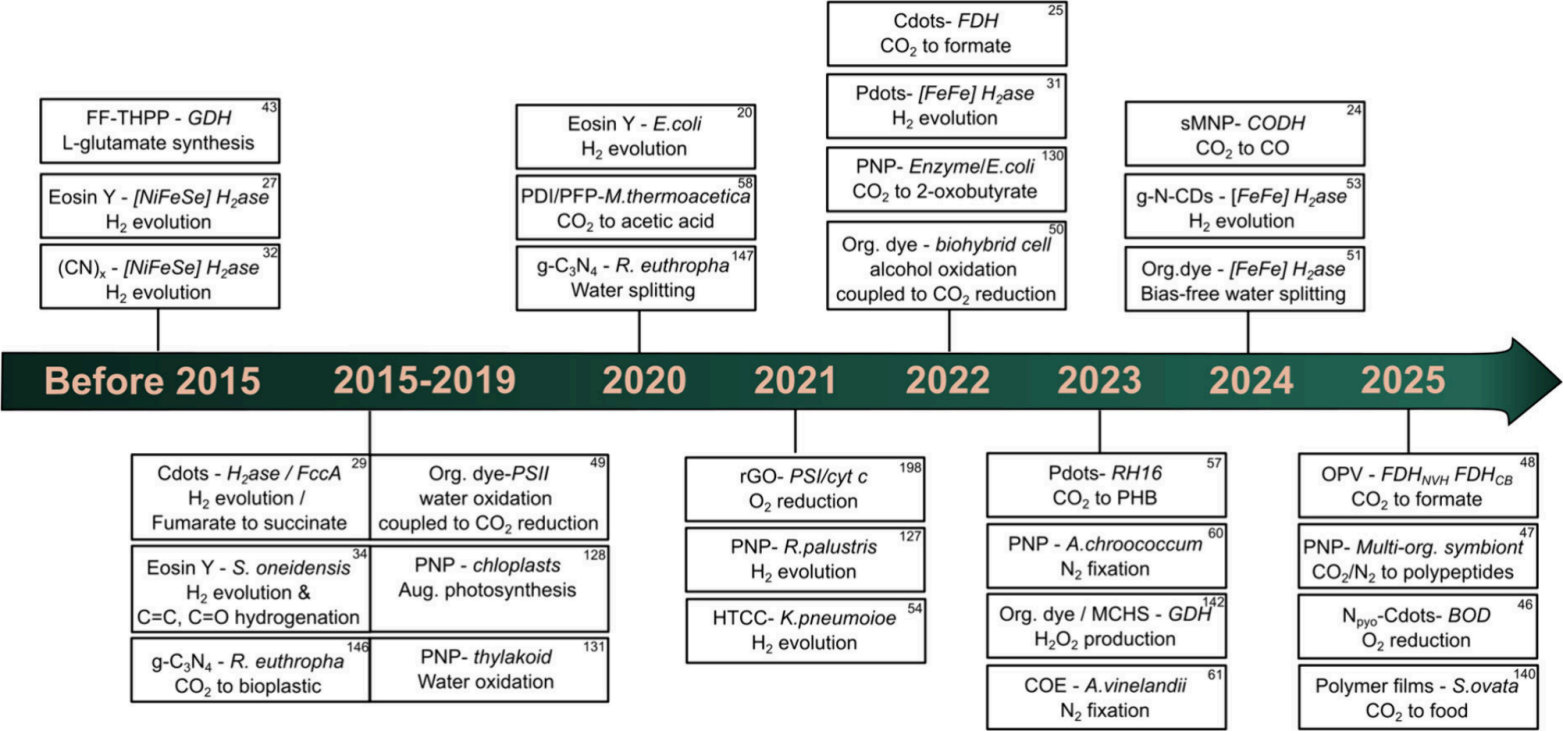
Historical development of biohybrid assemblies based on organic photosensitizers, where FccA -Fumarate reductase, g-CNx or g-C_3_N_4_ - graphitic carbon nitride, PDI - perylene diimide derivative, PFP - poly­(fluorene-co-phenylene), PNPs - polymer nanoparticles, rGO - reduced graphene oxide, HTCC - hydrothermal carbonation carbon, FDH-formate dehydrogenase, sMNPs -organic small molecule nanoparticles, COE-Conjugated oligoelectrolyte, PHB -poly­(3-hydroxybutyrate), CODH-carbon monoxide dehydrogenase. References are presented in the top right corner.^21,27,29,30,32,34,43,45–51^

Scheme 1 shows a representative biohybrid system using a biocatalyst for a reduction reaction. Primarily, the photochemical reaction is triggered by the absorption of ultraviolet and/or visible light by the abiotic OPS. Electrons that are promoted by light to higher energy states are then transferred directly to the catalytic active sites of biocatalysts (direct electron transfer, DET) or via redox mediators to the biocatalyst (mediated electron transfer, MET). For the reaction to proceed forward disadvantageous recombination processes need to be bypassed. At the same time the photogenerated holes are used to carry out an oxidation reaction, ideally to form a value-added product. Hereby, the biocatalyst is the core part of the biohybrid systems because it drives the redox reaction of interest. The systems can be transplanted to an electrode to build up a photocathode; then, the holes will be transported to a photoanode for another reaction in a photoelectrochemical cell; see Section 6. Table 1 presents a comparison of representative biohybrid systems using organic photosensitizers based on various biocatalysts, including enzymes and whole cells for different reactions.

**Scheme 1:**
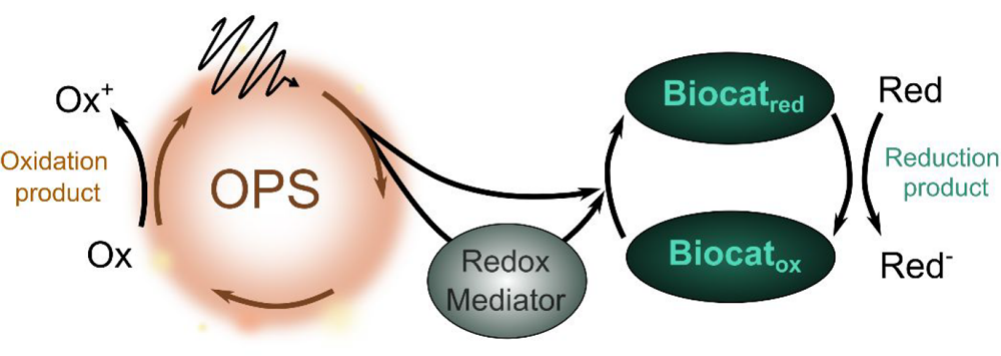
Biohybrid System Involving Biocatalyst for Reduction Reaction^a^

a Ox: Oxidant, OPS: Organic photosensitizer, Red: Reductant.

**Table 1:** Comparison of Representative Biohybrid Systems Based on the OPS

Biocat. type	OPS	Biocatalyst	Redox Mediator	Conditions	Light	Performance	Stability (h)	ref
**Hydrogen evolution**								
Enzyme	ASpCDs	[FeFe]-H_2_ase	MV^2+^ w/o	0.2 M TEOA, 0.1 M EDTA	LED (17W) λ > 420 nm 50 mW/cm^2^	1.73 μmol H_2_ mg^–1^ (hydrogenase) min^–1^	168	^30^
	polyheptazine carbon nitride polymer (melon CN_x_) loaded on TiO_2_ nanoparticle	[NiFeSe]-H_2_ase from *Desulfomicrobium baculatum*	Compare w/o MV^2+^	50 pmol Dmb [NiFeSe]-H_2_ase, 0.1 M EDTA (pH 6), 5 mg CN_x_-TiO_2_	Solar AM 1.5 G irradiation λ > 420 nm	8055 × 10^6^ μmol h^–1^ (within 72 h)		^33^
	P-dots	[FeFe]-H2ase from *Chlamydomonas rheinhardtii*	MV^2+^	16 μg/mL P-dots, 5 mM MV, 10/19/30%(v/v) TEOA, 158 pmol H_2_ase, pH 7	LED, 420–750 nm, 50 mW/cm^2^	88460 μmol H_2_ g_H2ase_^–1^ h^–1^	∼70	^31^
	Eosin Y	[NiFeSe]-H_2_ase from *Desulfomicrobium baculatum*	-	150 mM TEOA, 1 μmol EY, 10 pmol H_2_ase, 25 C, pH 7	Solar light simulator; AM 1.5 G, 100 mW/cm^2^, λ > 420 nm	13.9 ± 0.7 × 10^6^ μmol H_2_ mol_H2ase_^–1^ s^–1^	24	^27^
	Carbon dots (CDs)	[NiFeSe]-H_2_ase from *Desulfomicrobium baculatum*	MV^2+^	10 mg CDs, 50 pmol H_2_ase, 5 μmol MV, 0.1 M EDTA, pH 6, total volume 3 mL	Solar light simulator; AM 1.5 G, 100 mW/cm^2^, λ > 420 nm	26 ± 6 × 10^9^ μmol H_2_ h^–1^ (for CD-CO_2_)	>72	^29^
	CD-NHMe_2_^+^ or CD-CO_2_					24 ± 7 × 10^9^ μmol H_2_ h^–1^ (for CD-NHMe_2_^+^)		
	Porphyrin-Sensitized Nanoparticulate TiO_2_ Photoanode	[FeFe]-Hydrogenase *Ca* HydA from *Clostridium acetobutylicum* adsorbed to modified carbon felt electrode	-	1 mM PBS buffer, pH 7, 10 mM NADH	-	23.4 nmol H_2_ min^–1^	-	^52^
Whole-cell	Graphitic nitrogen-doped carbon dots (g-N-CDs)	Synthetic cells (nanoreactors) expressing [FeFe]-H2ase from *Clostridium beijernickii*	Compare w/o MV^2+^	2 nM nanoreactor, 100 mM EDTA, 150 μg/mL g-N-CDs, 50 mM PBS, pH 7.4	6200 K white LED, 29 mW/cm^2^	467 ± 61 h^–1^ (without MV^2+^)	-	^53^
						880 ± 154 h^–1^ (with MV^2+^) (TON)		
	Eosin Y	*E. coli* BL21(DE3)	MV^2+^ or DQ-OH	1 μM Eosin Y, 100 mM TEOA, pH 7.5, cell OD600 5, 10 mM RM	LED, λ > 420 nm, 50 mW/cm^2^	138 ± 19 μmol H_2_ mL^–1^h^–1^OD_600_^–1^ (for MV^2+^)	120	^5^
						453 ± 47 μmol H_2_ mL^–1^ h^–1^ OD_600_^–1^ (for DQ-OH)		
Perfect (Light) with a 420-nm cutoff filter.	Metal-free hydrothermal carbonation carbon (HTCC) microspheres	*Klebsiella pneumoniae*	-	1 g/L HTCC, SBC medium supplemented with 20 g/L crude glycerol, 10% (v/v) methanol, aliquot of 1 mL *K. pneumonia* cells	Xenon lamp (PLS-SXE300D, Beijing	0.3 μmol H_2_ h^–1^	-	^54^
**CO_2_ reduction**								
Enzyme	Phenoxanine-based organic molecule nanorod photosensitizer	[NiFe]-CODH II (from *Carboxydothermus hydrogenoformans*)	MV^2+^ or DQ-OH	38 μg/mL POZ-M(+)/POZ-M(-) pH 5.7, 0.5 M L-cysteine, 5 mM MV,250 pmol CODH	LED, 420–570 nm, 50 mW cm^–2^	With MV^2+^: 26 × 10^3^ μmol CO g_NRs_^–1^h^–1^ for POZ-M(+)	150	^24^
	POZ-M(+) (positively charged)					With DQ-OH: 530 × 10^3^ μmol CO g_NRs_^–1^ h^–1^		
	Amorphous Carbon Dots (a-CDs)	Formate Dehydrogenase (FDH)	-	10 mM EDTA in 100 mM NaHCO_3_	AM 1.5 G, 100 mW cm^–2^	23 ± 3 × 10^6^ μmol formate (mol FDH)^−1^ (during 24 h irradiation)	-	^25^
				1 mg, a-CDs, 1 μL 40 μM preactivated FDH, headspaced purged with CO_2_				
	PSI	[W]-formate dehydrogenase (FDH)	-	PSI and [W]-FdH immobilized on ITO; 100 mM MES buffer, pH 6	White light, 100 mW/cm^2^	Faradaic Efficiency of 15%	10% residual photocurrent after 22	^55^
	PCE10:EH-IDTBR	[W]-formate dehydrogenase (FDH)	-	PEC with hematite anode, polyethylene terephthalate (PET) and CO_2_ as substrates; FDH combined with carbonic anhydrase at cathode. Cathode compartment contained CO_2_-saturated NaHCO_3_ buffer (50 mM) and KCl (50 mM), pH 6.45	Simulated AM1.5G (100 mW cm^–2^)	11 μmol cm^–2^ h^–1^	Photocurrents decrease from 0.45 to 0.28 mA over 10	^56^
Whole cells/biofilms	Polymer dots (Pdots)	*Ralstonia eutropha H16* (RH16)	neutral red (NR)	10 μg/mL Pdots, OD_600_(RH16) = 0.5, 20 μM NR, 30 C, pH 7.4	Xenon fiber optics lamp with AM 1.5G filter	21.3 ± 3.78 mg/L poly-3-hydroxybutarate (PHB)	>48	^57^
	perylene diimide derivative (PDI) and poly(fluorene-co-phenylene) (PFP) forming a p-n-conjugated heterojunction layer	*Moorella thermoacetica*	Natural redox mediators rubredoxins and flavoproteins (on bacterial membrane)	Defined phtotosynthesis medium (DPM), 0.2wt% cysteine, PDI/PFP (molar ratio 1:1), *M. thermoacetica* OD_600_ = 0.2; 12h light/dark intervals for three days	50 mW/cm^2^	0.63 mM acetate (after 72 h light/dark intervals)	>72	^58^
**Fumarate reduction**								
Enzyme	Carbon dots (CDs)	Fumarate reductase (FccA)	-	1 mg CDs, 0.22 nmol FccA, 10 mM fumarate, 0.1 M EDTA	Solar light simulator; AM 1.5 G, 100 mW/cm^2^, λ > 420 nm	1.7 ± 0.2 × 10^3^ mol succinate (mol_FccA_)^−1^ h^–1^ (for CD-NHMe_2_^+^)	>24	^29^
	CD-NHMe_2_^+^ or CD-CO_2_^–^							
**Cytochrome P450 Catalysis**								
Whole Cell	Eosin Y	Various bacterial and human P450s heterologously produced in *E. coli* ^a^	-	EY (20 μM), and 7-ethoxycoumarin (1 mM) in 0.2 mL of potassium phosphate (50 mM)–TEOA (100 mM) mixture buffer (pH 7.4)	White light	TON (c(product)/c(enzyme)) of enzyme BM3m2 is 16 at 18h for product 7-hydroxycoumarin	-	^59^
Enzyme	Deazaflavins	P450 monooxygenase BM3 from *Bacillus megaterium*	-	Reaction performed in presence of lauric acid	-	TOF 117 h^–1^ for hydroxylation of lauric acid	-	^22^
**Nitrogen Fixation**								
Whole Cell	poly-(fluorene-alt-phenylene) (PFP) (light harvesting)	*Azotobacter chroococcum*	-	PFP (0.5/1/2/5 μM), *A. chroococcum*, 12 h dark/light cycles	-	107.7 nmol C_2_H_4_/10^8^ cell	48	^60^
	Conjugated oligoelectrolyte (COE-IC)	*Azotobacter vinelandii*	-	20 μM COE-IC in PBS	-	22 nmol NH_3_/mL under light conditions	-	^61^
**Photosynthesis I/II**								
Whole Cells	Carbon Dots (CDs)	Chloroplasts from rice plants	-	200 μg chlorophyll, 300 μg/mL CDs, 360 μM 2,6-dichlorophenolindophenol (DCPIP)	600 1×	- (To improve rice production)	-	^62^
	N-doped CDs	*Malus hupehensis* seedlings	-	300 mg/L CDs in cultivated Hoagland solution for seedlings	UV-A irradiation	- (To improve apple production)	-	^63^
	Dual-emissive carbon dots	Spinach chloroplasts	-	5 mL chloroplast suspension, mixed with CDs in sucrose buffer	Xenon lamp, 4 mW/cm^2^ (200–1000 nm)	- (To improve ATP production)	-	^64^
	Carbon dots	Mung bean leaf chloroplasts	-	378.45 μg/mL chloroplasts, 0.33 M sorbitol, 2 mM NaEDTA, 1 mM MgCl_2_, 50 mM HEPES, pH 7.6	-	Enhance ATP and NADPH production	-	^65^
				0.88 mM DCPIP				
							-	^45^
Enzyme	PSI-(On photocathode)		-	PSI immobilized on ITO; 100 mM MES buffer pH 6, and 400 mM KCl; photocurrents determined at an applied potential of −100 mV vs Ag/AgCl	-	–8.5 ± 0.8 μA cm^–2^ at 100 mW cm^–2^	-	^66^
	PSI-ATTO532-(On photocathode)	CytC (coimmobilized)	-	PSI immolized on ITO; 5 mM PPB pH 7; photocurrents determined at an applied potential of −100 mV vs Ag/AgCl	-	–60.9 ± 9.5 μA cm^–2^ at 100 mW cm^–2^	-	

a Tabulated information refers to bacterial P450s

Understanding how efficiently this biocatalyst performs the targeted transformation is crucial for evaluating and improving the overall system performance. The following section therefore examines the advancements and limitations associated with biocatalysis in such systems.

### 2.2 Biocatalysis: Advancement and Limitations

The design and performance of biohybrid systems strongly depend on the choice of biocatalyst, which can be broadly subdivided into either isolated enzymes, subcellular organelles, or whole-cell systems. Therefore, these three types of biohybrid systems should be compared with respect to their advantages and disadvantages. On a more general note, large-scale biotechnology employs both isolated enzymes and whole-cell systems, depending on process requirements and cost-effectiveness. Isolated enzymes are commonly favored for reactions where high specificity, ease of process control, and minimal side-reactions are critical, especially in industries such as pharmaceuticals and fine chemicals. Whole-cell biocatalysts, however, are often used for multistep reactions, cofactor regeneration, and when enzyme stability or protective environments are needed, making them prominent in large-scale fermentations and bulk chemical synthesis due to reduced costs and operational robustness.

For enzymatic systems that work with a purified enzyme as a catalytic platform, high substrate affinity and selectivity of the biocatalyst are critical advantages, enabling a high level of control. Thus, side reactions that might compete with the reaction of interest or produce byproducts that impact the enzymatic activity or overall system performance are unlikely. This is advantageous for system stability and longevity, and it further allows for a better control and characterization of the systems with respect to turnover numbers, frequencies, and other mechanistic details.^20,34,67,68^ Additionally, enzymatic systems can be optimized in a tailored fashion because the photocatalytic components can be chosen to optimize the thermodynamic driving force for electron transfer to or from the enzyme.^6,19,24,69^ With matching redox potentials of the different abiotic components, the turnover numbers and frequencies can be increased, and the efficiency of the systems can be improved, accordingly.^70–75^ When considering the artificial components, it is also essential that the photocatalytic components do not impact the enzymatic activity, for example by binding to the substrate pockets, changing protein conformations and thereby impacting the protein structures and folding, or producing degradation products that have an effect on the protein.^34,68^

A major disadvantage of enzymatic systems is that the enzymes must be purified. This process is tedious and can be economically demanding. Furthermore, it can be difficult to maintain the enzyme activity over a longer period, as isolated proteins can be significantly more unstable compared to when they are kept in a physiological whole-cell environment. With this being said, encapsulation of enzymes into various polymer networks has been shown to ensure enzyme stability under various stress conditions.^76–79^

In contrast, whole-cell-based systems are usually easier in practice as there is no need to purify the enzymes. Especially when using well-studied commercial organisms like *E. coli*, the growth conditions are generally straightforward and the growth-rate of the biocatalyst is high. In general, it is advisable to use well-studied organisms as catalytic platforms, as they are mostly fully sequenced and easy to cultivate after established protocols and molecular biology tools readily available enabling heterologous enzyme production and metabolic engineering. Yet, it has to be mentioned that this is also highly dependent on the specific reaction pathway desired and by extension the required enzyme(s), as some require a certain expression host and special conditions, which might not be met by commercial expression hosts like *E. coli*.^80^ As indicated in Table 1, biohybrid systems for N_2_ fixation for example, commonly rely on *Azotobacter* taking advantage of the organisms innate ability to reduce N_2_.

Another advantage of whole-cell systems as compared to enzymatic systems is the fact that cell division provides a straightforward path for catalyst regeneration, thus ensuring that the catalyst effectively becomes “self-healing”. The whole-cell environment also ensures a suitable environment for the enzyme catalysts and can provide partial protection from the external environment. For instance, [FeFe]-hydrogenases are highly sensitive toward O_2_ and CO and are known to be deactivated under aerobic conditions. However, whole-cell systems can provide a partial protection from these atmospheric gases, as exemplified by the report by Lorenzi et al. that showed that *E. coli* cells ensured that the CrHydA1 [FeFe]-hydrogenase remained partially active upon exposure to 5% oxygen.^67^ Further protection can be achieved through intracellular compartmentalization, as exemplified by encapsulating hydrogenases in carboxysomes.^81^ Indeed, this strategy of compartmentalization is also observed in biology, as exemplified by, for example, heterocyst formation, enabling certain cyanobacteria to perform N_2_ fixation in parallel to oxygenic photosynthesis.

On the other hand, whole-cell systems feature the key disadvantage that side reactions are always possible and that these systems are too complex to be fully understood. Metabolic processes, stress reactions, and electron transfer processes from redox mediators or photosensitizers to other redox-active components in the cell are all happening simultaneously, and it is hard to monitor all the processes and to assess how efficient the electron transfer to or from the enzyme of interest works. Side reactions can also lead to the production of cytotoxic components that eventually lead to apoptosis and, thus, decrease the long-term stability of these biohybrid systems. A good example of this is the aforementioned production of glycol aldehyde as part of TEOA oxidation (see Section 5.1.3 for details) or the production of highly reactive radicals upon reduction of MV^2+^.

## 3 Organic Photosensitizers

Organic photosensitizers (OPSs) can be classified according to their structural, photophysical, and interfacial properties. Structurally OPSs are often grouped into small organic molecules, polymeric materials, and supramolecular assemblies. However, with the rapid advancements of carbon-based nanomaterials, in this review, we have grouped OPSs based on the distinct structural characteristics of these materials in biohybrid assemblies. Accordingly, we distinguish the following classes: (a) single molecular organic photosensitizers, (b) carbon dots, (c) graphene-based materials, (d) organic aggregation nanoparticles (NPs) including polymer nanoparticles and small molecule nanoparticles, and (e) carbon nitride materials. Grouping them in this way provides a framework for targeted optimization and rational hybrid design strategies of semiartificial systems based on organic photosensitizers. In Section 3.1 we first introduce the fundamental requirements for organic photosensitizers, particularly in terms of their compatibility with biocatalysts. We then present a detailed overview of various groups of OPSs that have been integrated with biocatalysts, including molecular photosensitizers (Section 3.2), carbon dots (Section 3.3), graphene-based materials (Section 3.4), organic aggregation nanoparticles (Section 3.5), carbon nitride materials (Section 3.6), and other groups of organic-based photosensitizers (Section 3.7).

### 3.1 Fundamental Criteria for Organic Photosensitizers in Semiartificial Photosynthesis

An organic photosensitizer in the semiartificial photosynthesis system must fulfill several essential requirements to ensure efficient and stable photobiocatalytic performance:

(1) The materials should absorb light efficiently across the visible and near-infrared spectrum to maximize solar energy use, while filtering UV light to protect UV-sensitive biocatalysts.^82^
(2) OPSs should have redox potentials suited for key biocatalytic reactions, with sufficiently long-lived and mobile charge carriers to drive the reactions; DET to the biocatalyst is especially advantageous.^83^
(3) Biocompatibility must be evaluated, distinguishing between that of the organic photosensitizer itself and that of other components required for the reaction.
(4) The size, morphology, and structural compatibility of the photosensitizer relative to the biocatalyst should be considered, as these factors may influence interaction between the photosensitizer and the biocatalyst. Upon surface-modified, OPSs can localize nearby biocatalyst active sites, with compatible surface charges to enable efficient charge transfer and overall system performance.
(5) Finally, OPSs must be photostable, thermostable and chemically stable under the operating conditions.

A comparison of different groups of organic photosensitizers based on their ability to meet these requirements for the construction of biohybrid systems is presented in Table 2. While designing an “ideal” organic photosensitizer that fulfills all performance criteria is highly challenging, understanding the intrinsic strengths and weaknesses of each class allows for the strategic combination of materials to overcome individual shortcomings and advance the efficiency and versatility of biohybrid catalysis and thus is discussed below.

**Table 2:**
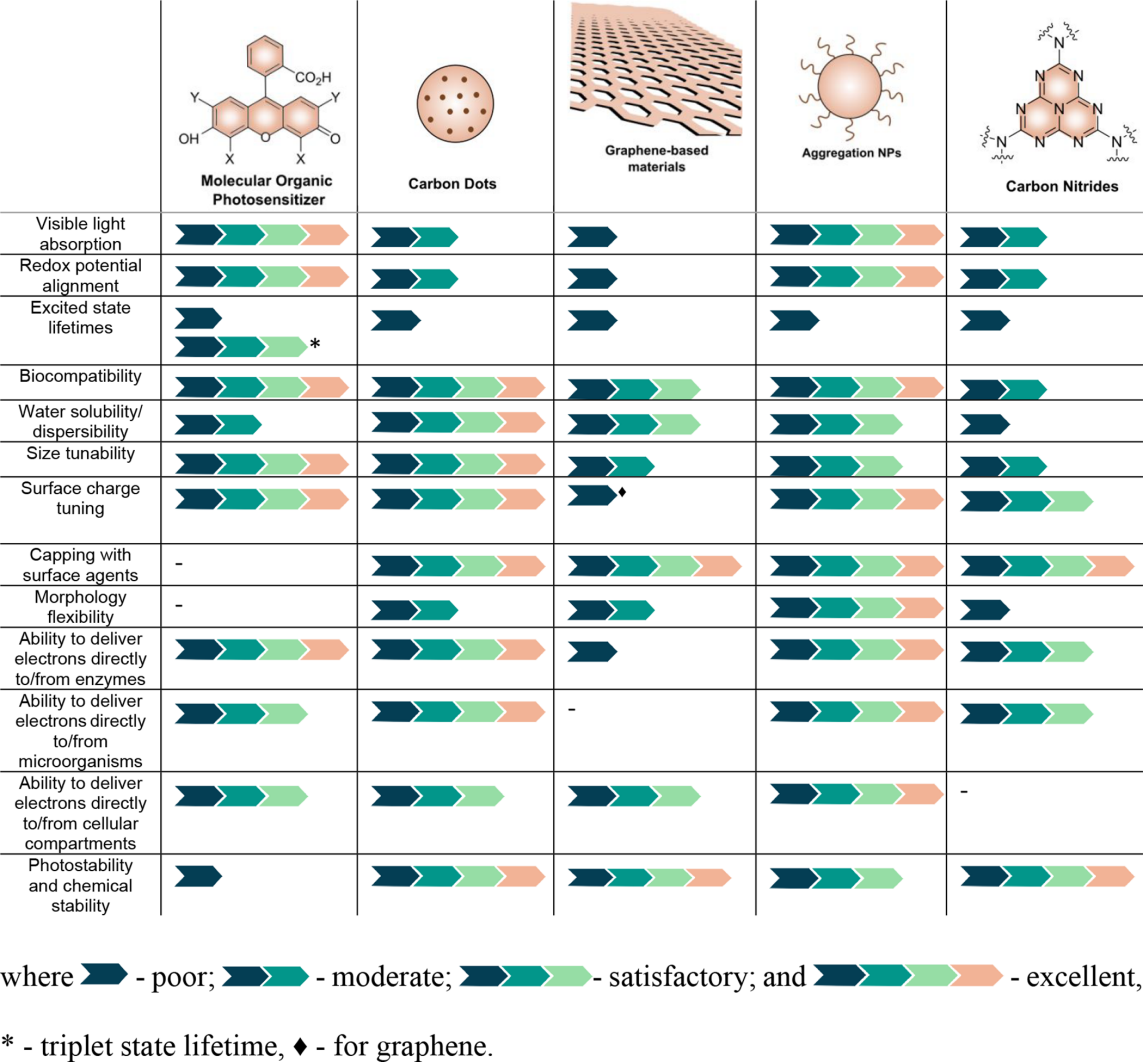
Comparative Assessment of Various Groups of Organic Photosensitizers Is Based on Their Ability to Potentially Meet Key Design Requirements for Biohybrid Assemblies

### 3.2 Single Molecular Organic Photosensitizers

One of the reasons behind the intense investigation of molecular OPSs as compared to other nonmolecular organic photosensitizers or many inorganic photosensitizers, is their flexibility of reaching the active sites of biocatalysts due to their small dimensions and suitable photophysics, that allows DET.^20^ Molecular organic photosensitizers exhibit broad absorption spectra and high quantum yields, allowing for more efficient utilization of visible light and improved rates of photocatalytic reactions.^50,84^ Their absorption is determined by the HOMO–LUMO energy gap.^85^ Molecular OPSs are biocompatible, cost-effective, and environmentally friendly.^50^ Gamache et al. have shown that xanthene organic light harvester, Eosin Y (EY), itself does not show any toxicity to cells, both with and without light exposure, and that rather the decomposition of the photocatalytic components or side products formed during the photocatalytic hydrogen production stand behind the toxicity to host cells.^5^

Honda et al. revealed that molecular OPSs (EY) can absorb visible light more effectively than TiO_2_ and have better water dispersibility. Moreover, EY avoided the necessity of harmful redox mediators (i.e., methyl viologen, MV^2+^) in whole-cell biocatalytic H_2_ formation with *Escherichia coli* (*E. coli*).^20^ Ainsworth et al. compared the efficiency of several organic molecular photosensitizers, such as xanthenes, and flavins, with that of benchmark inorganic photosensitizers, including [Ru­(bpy)_3_]^2+^ and [Ru­(bpy)_2_{4,4′-(PO_3_H_2_)_2_bpy}]­Br_2_ (RuP).^86^ The comparison was based on their performance in either the photoreduction of extracellular cytochromes MtrC and OmcA acting together with the outer-membrane-spanning porin·cytochrome complex (MtrAB) or in hydrogen production.^85^ It was shown that while only less than 15% photoreduction of the cytochromes was observed with the selected inorganic photosensitizers, organic photosensitizers like riboflavin (RF), flavin mononucleotide (FMN) and flavin adenine dinucleotide (FAD) plateaued at 60% photoreduction. At the same time EY, fluorescein, and proflavine showed complete 100% reduction of the cytochromes, furthermore highlighting the potential of organic photosensitizers.^86^ In additional work Rowe et al. found that the rate of MV^2+^ reduction under the conditions suitable for *Shewanella oneidensis (S. oneidensis)* increased when photosensitizers with a metal core were replaced with solely organic in the following order [Ru­(bpy)_3_]^2+^ < RuP < fluorescein < proflavine < EY.^34^ In accordance with these data, organic photosensitizers showed more efficient photobiocatalytic hydrogen generation with *S. oneidensis*.

#### 3.2.1 Triplet State

Upon light absorption, molecular organic photosensitizers are promoted from the singlet ground state (S_0_) to an excited singlet state (S_1_). In many organic dyes, particularly halogenated xanthene and flavins, efficient intersystem crossing (ISC) occurs due to enhanced spin–orbit coupling and favorable S_1_–T_1_ energy gaps, generating a triplet excited state (T_1_) (Scheme 2). While the S_1_ state is typically higher in energy and can exhibit slightly stronger instantaneous redox potentials, it is short-lived, usually at the nanosecond scale). In contrast, the T_1_ state is lower in energy but spin-forbidden to relax directly to S_0_, resulting in significantly prolonged lifetimes, normally at the micro- or millisecond scale. This extended lifetime increases the probability of productive intermolecular electron transfer with biocatalysts before charge recombination occurs. Therefore, in most organic photosensitizers used for semiartificial photosynthesis, the triplet state is the dominant reactive species, balancing sufficient redox driving force with kinetically favorable lifetimes.

**Scheme 2:**
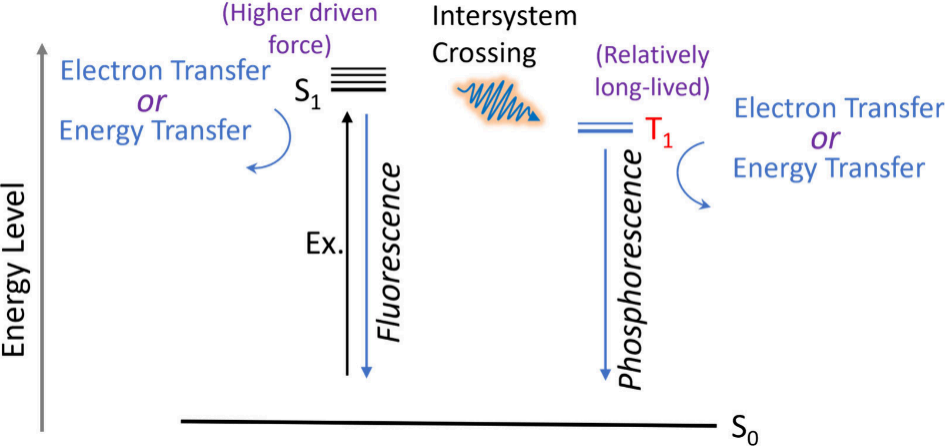
Singlet State to Triplet State

Up to date, molecular organic photosensitizers have been used to drive various chemical reactions, like hydrogen evolution,^5,20,27,28^ hydrogenation of C=O and C=C bonds,^21,34^ CH-activating hydroxylation,^22^ enantiospecific hydroxylation of nonactivated C–H bonds and epoxidations of C=C bonds,^87^ synthesis of L-glutamate,^88^ fatty-acid hydroxylation,^89^ stereoselective Baeyer–Villiger reaction,^23^ water oxidation,^29^ CO_2_ reduction,^30^ *etc.* (Figure 3).^59^ However, OPSs still face challenges, such as limited photostability^5^ and chemical stability. Moreover, many of the existing organic molecular photosensitizers, such as diketopyrrolopyrrole-based dyes, are laborious to synthesize. Below we discuss the more detailed features of these photosensitizers.

**Figure 3:**
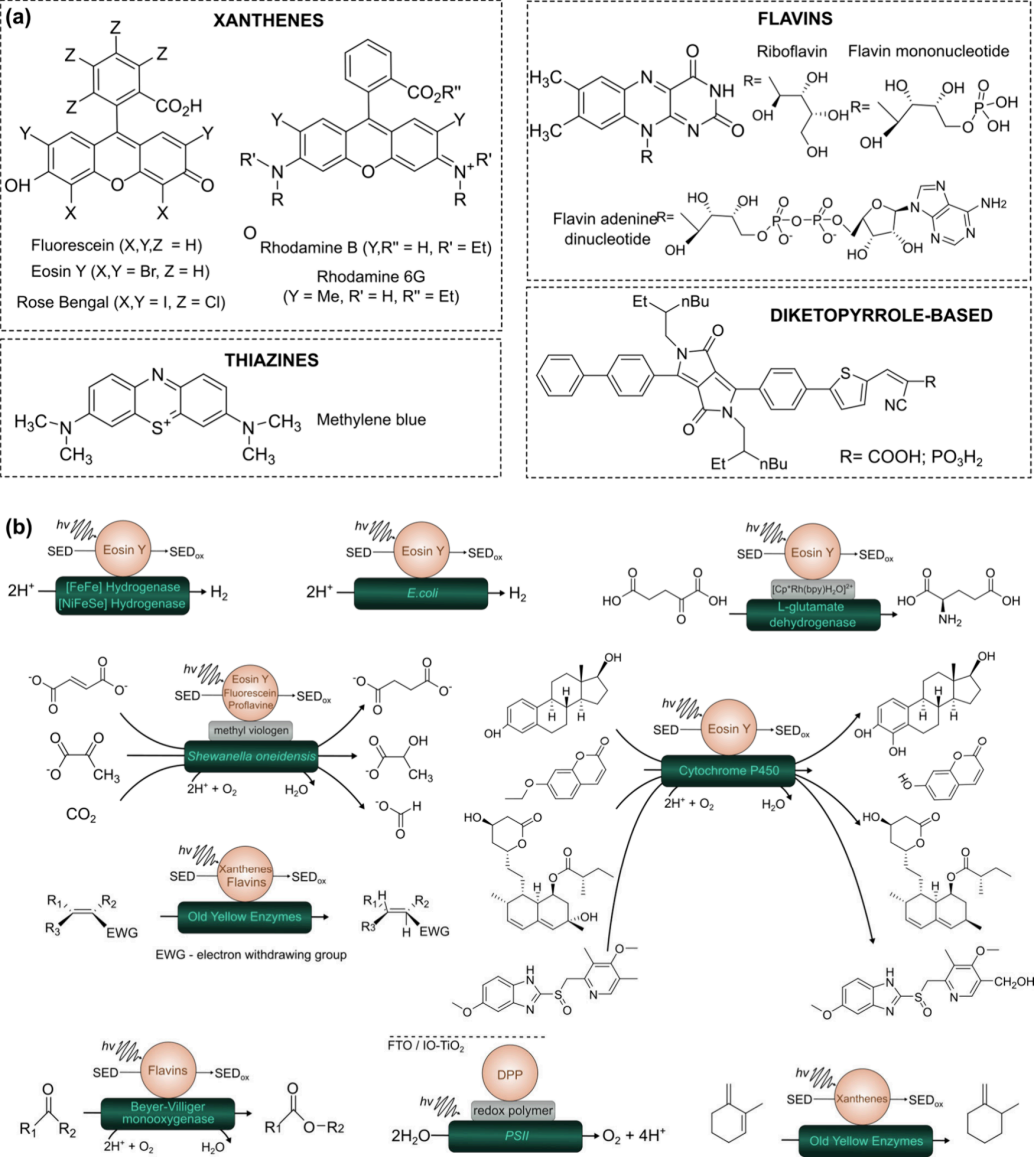
(a) Structures of molecular OPSs used for photobiocatalytic transformations; (b) the reaction schematic pathways.^5,20–23,27,28,34,49,50,59,87–89^

***Xanthenes*** (e.g., EY, Rose Bengal) are low-cost organic photosensitizers that efficiently absorb visible light (typically in the 500–600 nm range), generating photoexcited electrons with singlet–triplet transitions.^90^ In contrast to fluorescein, EY and Rose Bengal undergo rapid intersystem crossing (ISC) to their triplet state, with the quantum yield of ISC (ϕ_ISC_) increasing across the series due to the heavy atom effect introduced by halogenation.^91^ As a result, these organic photosensitizers exhibit very short singlet-state lifetimes (∼0.5–2.7 ns), making their triplet states the dominant reactive species in photoredox processes (∼24 μs for EY).^85,91^ Park et al. investigated a series of different xanthene OPSs to study their capability for photochemical NADH (nicotinamide adenine dinucleotide) regeneration, where they observed increased photoregeneration in the order of heavy halogen atom substitution. As the substituent became heavier, the OPSs’ electron-donating ability increased in the order H < Cl < Br < I.^92^ The low-lying triplet excited state (^3^OPS*) of xanthene OPSs enables both moderate oxidative and reductive electron transfer, making them highly versatile in photocatalytic applications.^90,93^ The relatively high triplet yields and energies of halogenated fluorescein stand behind the tendency to undergo triplet energy transfer, often leading to the generation of singlet oxygen (^1^O_2_),^94^ that should be specifically considered in the presence of electron donors.

Owing to their properties and commercial availability, xanthene dyes (e.g., EY and Rose Bengal) are widely used to facilitate electron transfer to biocatalyst active sites.^4,12^ It was shown that EY with E^0^(^3^EY*/EY^–^) = + 1.07 V vs standard hydrogen electrode (SHE) and E^0^(EY^+^/^3^EY*) = −0.87 V vs SHE can efficiently deliver electrons both indirectly^28,92^ and directly^21,27,59^ to different types of biocatalysts (including various enzymes and microorganisms).^20^ One of the unique features of EY that makes it stand out among other photosensitizers is its ability to serve as an intracellular agent, for example entering *E. coli*.^5^ Rose Bengal also showed the ability to directly transfer photoexcited electrons to the FMN prosthetic group of oxygen-independent flavoenzymes, such as Old Yellow Enzymes (OYEs).^21^

Xanthenes, like many other molecular organic photosensitizers, suffer from photobleaching when exposed to light for extended periods. The instability of EY mainly arises from the degradation of its reductively quenched intermediate, EY_red_, electron transfer from which to the biocatalyst or to an alternative primary electron acceptor is clearly insufficient to fully prevent degradation of the photosensitizer, thus often requiring the addition of redox mediator.^5^

***Flavins*** (e.g., RF, FMN, and FAD) are organic photosensitizers based on the isoalloxazine ring system that generally absorb light with maxima around 370 and 450 nm. They play a crucial role in the cellular metabolic processes. Upon illumination, flavins efficiently undergo intersystem crossing to form a triplet state with high quantum yields, similar to halogenated fluoresceins. In this triplet state, flavins act as strong oxidants due to their high oxidation potential.^84^ They can oxidize external electron donors, such as ethylenediaminetetraacetic acid (EDTA), generating reduced flavin species capable of subsequently reducing oxidized forms of biocatalysts. Typically, reductive quenching of the excited state yields semiquinones that undergo rapid disproportionation to generate a hydroquinone, that is acting like a photoreductant.^95^ The hydroquinone forms of FMN and FAD have *E*_m_ values much lower than the corresponding potentials for EY, fluorescein, or proflavine that in some cases lead to much lower photoreduction yields.^86^ To avoid the reliance on costly NAD­(P)­H, Reetz et al. developed a photoactivation strategy for flavoenzymes that employed free flavin molecules excited by irradiation with visible light coupled with a sacrificial electron donor to promote ketone oxidation.^23,96^ When free FAD was used to activate Baeyer–Villiger monooxygenase (BVMO), high enantioselectivity (97%) was achieved, comparable to NADPH-based or whole-cell systems.^97,98^ However, the initial TOF was significantly lower than that of NADPH-driven reactions due to the rapid reaction of photoreduced flavins with O_2_, generating reactive oxygen species. To minimize such side reactions, deazaflavin was proposed as a more oxygen-stable alternative, allowing to achieve a CH-activating hydroxylation.^22^ The study was extended further to drive enantiospecific hydroxylation of nonactivated C–H bonds and epoxidations reactions.^87^ Using flavins (RF, FMN, FAD, and deazaflavins) as photosensitizers and EDTA as the electron donor, Hollmann’s group showed direct photoactivation of the oxygen-independent flavoenzymes homologue from *Bacillus subtilis* (YqjM) for the reduction of ketoisophorone.^99^

***Thiazines*** (e.g., methylene blue, MB) are six-membered heterocyclic compounds containing one nitrogen and sulfur atom presented either at the 1,2- 1,3- or 1,4-positions. Currently there are not many examples of thiazines that have been used in semiartificial photosynthesis. One of the known examples is methylene blue.^100^ Similarly to other molecular OPSs discussed here, methylene blue has a short singlet lifetime (1.0 ns) and a relatively large triplet yield,^101,102^ causing the triplet ^3^MB^+^ to be the most relevant excited state. Thus, even though methylene blue (MB^+^) absorbs light at a relatively low energy, its triplet state (^3^MB^+^) acts as a moderately strong oxidant. Additionally, it was shown that MB^+^ can go beyond single-electron redox transfer, undergoing sequential electron transfer (ET) or hydrogen atom transfer (HAT). It was shown that under photoexcitation MB can directly activate native horseradish peroxidase during the catalytic reduction of H_2_O_2_.^103^

##### 3.2.1.1 Diketopyrrolopyrrole-Based Photosensitizers

Even though most molecular organic photosensitizers reported to be active in semiartificial photosynthesis belong to just a few classes of organic compounds discussed above, there are a few examples of diketopyrrolopyrrole-based (DPP) dyes used for photoelectrochemical water oxidation, CO_2_ reduction or alcohol oxidation coupled with photoreduction of CO_2_ to formate.^49,50,104,105^ Antón-García reported both organic dyes based on a DPP core with either carboxylic acid (DPP-CA) or phosphonic acid (DPP-PA) anchoring groups that outperformed a ruthenium-based photoabsorber (RuP) known as as a benchmarks, furthermore highlighting the benefits of organic photosensitizers.^50^ It is worth noting that most of the reported DPP-based dyes required the addition of TiO_2_ or Os-based redox polymer (P-Os, poly­(1-vinylimidazole-co-allylamine)-Os­(bipy)­2Cl) to improve the wiring with the biocatalyst.^105^

#### 3.2.2 Design Criteria

Select molecular OPSs with good water solubility and their excited-state redox potentials matched to the biocatalyst and with sufficiently long-lived triplet states to enable efficient electron transfer while minimizing recombination; Avoid dyes prone to rapid photobleaching or excessive ROS generation unless oxygen management or fast electron extraction is ensured; Prioritize aqueous compatibility, biocompatibility, and structural simplicity to balance performance with operational stability and scalability.

### 3.3 Carbon Dots

Carbon dots (CDs) are low-cost, quasi-spherical carbon-based nanoparticles, typically less than 10 nm in diameter. Upon light absorption, CDs generate photoexcited charge carriers that can participate in electron or energy transfer processes with nearby biocatalysts, enabling their use as photosensitizers. CDs have found their application in bioimaging, sensing and more recently in semiartificial photosynthesis.^30^ Due to their small size and unique chemical structure, CDs possess a high surface-to-volume ratio, which provides several advantages, including enhanced catalytic activity and strong photoluminescence.^30^ Several features make CDs particularly attractive compared to other photosensitizers, including excellent water dispersibility and high photo-, thermo-, and chemical stability,^106–108^ which arise from their graphitic structure, hydrogen bonding, and surface functional groups. These properties enable CDs to maintain their catalytic performance over extended periods, even under demanding conditions. Their nanoscale dimensions facilitate efficient DET to biocatalyst active sites^30^ or, in some cases, transmembrane electron delivery to intracellular reactive centers.^53^ Moreover, the favorable photophysical properties of CDs support both energy and charge transfer processes.^45,64,65^ CDs are also advantageous due to their low cost,^65^ low toxicity,^65^ tunable light absorption (extending beyond 600 nm), and adjustable redox characteristics through surface functionalization or heteroatom doping. Finally, their surfaces can be readily modified to further enhance performance and compatibility with various systems.^25,29,46,109,110^ The performance of CDs in photobiocatalysis depends strongly on their electronic structure, interfacial interactions with biocatalysts, and the efficiency of charge separation. These aspects can be tuned through several material design strategies discussed below. These strategies can be broadly categorized into (a) electronic structure engineering through heteroatom doping, (b) interfacial engineering via surface functionalization, and (c) structural control through size tuning and covalent wiring to biocatalysts. In recent years, CDs have found their application in photobiocatalytic reactions such as hydrogen evolution,^29,30^ N_2_O reduction,^111^ fumarate protoconversion,^29^ CO_2_ reduction,^25^ and oxygen reduction (Figure 4).^46^

**Figure 4:**
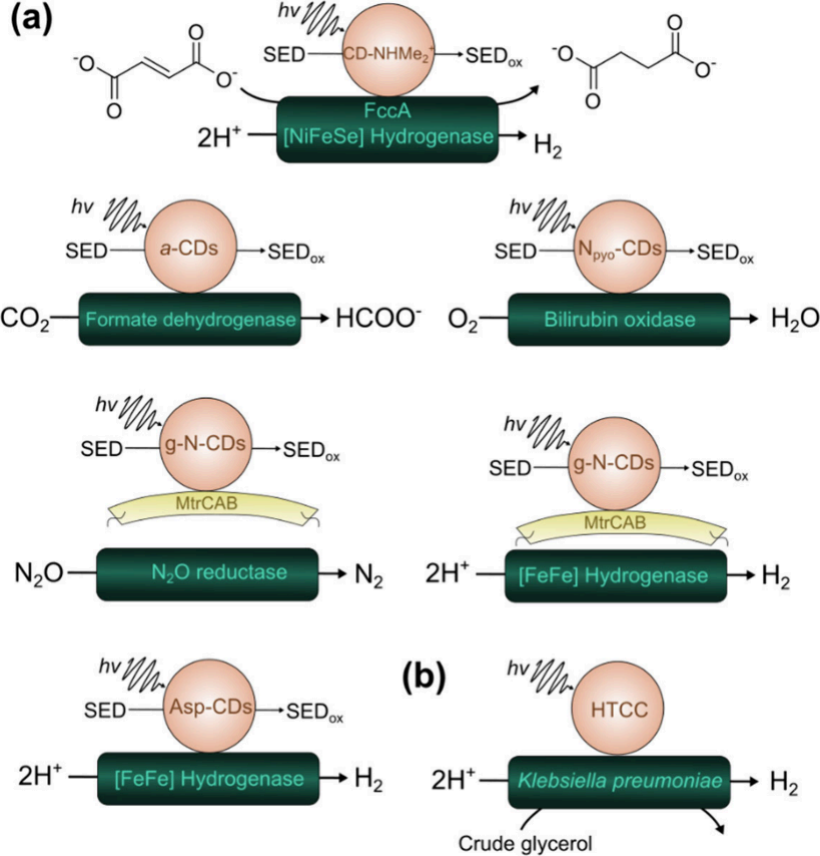
Photobiocatalytic transformations performed with the help of (a) CDs and (b) hydrothermal carbonation carbon (HTCC).^25,29,30,46,111^

#### 3.3.1 Electronic Structure Engineering: Heteroatom Doping

Heteroatom doping (e.g., N, S) of CDs is one of the common approaches to tune their electronic characteristics, surface charges, local chemical features and to unveil the overall structure–activity relationships.^46,63,110^ Fine control of nitrogen doping and corresponding tuning of the LUMO energy levels, allowed to synthesize pyrrole nitrogen-doped CDs (N_pyo_-CDs) that could efficiently deliver electrons to the active site of bilirubin oxidase,^46^ for oxygen reduction. It was found that nitrogen-doped CDs, N_pyo_-CDs, had much more favorable absorption, extending to the visible region. Furthermore, Jing et al. reported enhanced photosynthetic efficiency from nitrogen-doped CDs to plastoquinone-9.^63^ Biohybrid assemblies composed of N, S-doped CDs and *S. oneidensis* showed 2.6-fold enhanced extracellular electron transfer, compared to the raw microorganism. The ability of CDs to deliver electrons efficiently to the biocatalyst was also utilized to build photosynthetic nanoreactors with transmembrane electron transfer.^53^ It was shown that graphitic nitrogen doped CDs (g-N-CDs) could efficiently deliver photoenergized electrons to synthetic cells containing [FeFe]-hydrogenase from a *Clostridium beijerinckii* via a multiheme transmembrane protein from *S. oneidensis* MR-1 (MtrCAB).

#### 3.3.2 Interfacial Engineering: Surface Functionalization

Surface engineering is critical for controlling electrostatic interactions, enzyme orientation, and proximity between the PS and electron relays or active sites. Functionalization with charged capping agents can significantly enhance direct electron transfer between CDs and the biocatalyst. Hutton et al. showed that by functionalizing CDs with positively charged ammonium-terminated capping agents, more efficient interfacial interactions with the surface of negatively charged enzymes (e.g., fumarate reductase and hydrogenase) could be achieved.^29^ Furthermore, it was shown that not only the surface charge of CDs plays a role in efficient electron transfer but also the relative dimensions of CDs to the biocatalyst. Hola et al. synthesized significantly smaller negatively charged aspartic acid-based CDs (Asp-CDs) to match a positively charged pocket on the surface near the active site of hydrogenase that led to substantial enhancement of DET.^30^ The importance of the surface charge of CDs for effective DET to the distal FeS cluster region of formate dehydrogenase was later supported by Badiani et al. as well.^25^ Enhanced specific enzyme activity toward the oxidation of ABTS (2,2-azinobis (3-ethylbenzothiazolin-6-sulfinic acid) diammonium salt) was observed by Li et al. for laccase enzyme in the presence of phosphate-functionalized CDs.^112^ Zhang et al. synthesized chiral CDs that enhanced photosynthesis, boosting chlorophyll content, and promoting RuBisCo (ribulose-1,5-bisphosphate carboxylase/oxygenase) activity.^113^

#### 3.3.3 Structural Control and Covalent Wiring

Beyond electrostatic assembly, covalent conjugation provides defined electron-transfer pathways (Figure 5). In order to engineer an efficient electron transfer pathway in biohybrid systems, Zhang et al. have designed g-N-CDs that were covalently bound to the outermembrate decaheme protein, MtrC from *S. oneidensis*.^109^ Functionalization of g-N-CDs was achieved through a maleimide moiety by either carbodiimide chemistry or acyl chloride activation. Both methods are simple and can be readily applied to assess the labeling of other proteins and enzymes with CDs.

**Figure 5:**
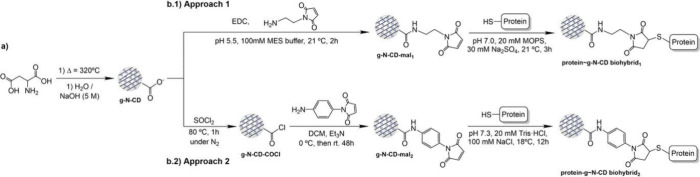
Schematic representation of the synthesis of (a) g-N-CDs and their maleimide-functionalized derivatives *via* two distinct strategies: (b.1) carbodiimide-mediated coupling and (b.2) acyl chloride activation. The resulting functionalized g-N-CDs were then conjugated to a cysteine-containing protein using the respective methods, referred to as approaches 1 and 2 (EDC, 1-ethyl-3-(3-dimethylaminopropyl) carbodiimide hydrochloride). Reproduced from ref 106. Available under a CC-BY 4.0 license. Copyright (2023) Wiley.

#### 3.3.4 Photophysical Limitations to Be a Good Photosensitizer of CDs

Despite their advantages, CDs exhibit intrinsic limitations. CDs are the relatively low fraction of long-lived excited states that can deliver charges to a biocatalyst, dissipation of photogenerated electrons as fluorescence and trapping of photogenerated electrons and holes in defects on the surfaces of CDs.^114,115^ Martindale et al. studied the photophysical properties of CDs. It was found that the majority of the excited states relaxed on the picosecond time scale that was much faster than the diffusion time scale required for bimolecular charge transfer. However, up to 10% of the excited states exhibit much longer lifetimes, in the microsecond to second range, and were attributed to the primary contributors to charge transfer.^114^ It should be also noted that there are no reports on CDs utilization in oxidation biohybrid photocatalytic transformations. Moreover, the existing reduction reactions involving CDs utilize primarily enzymes, with limited reports on the direct utilization of microorganisms or whole cells has been reported to the best of our knowledge.^116^

#### 3.3.5 Hydrothermal Carbonation Carbon

Hydrothermal carbonation carbon (HTCC) is a distinct carbon-rich material with related but structurally different characteristics from CDs, which is produced by a thermochemical process called hydrothermal carbonization of carbohydrates such as glucose, starch, grass, or various waste materials. Similar to g-C_3_N_4_, HTCC is an effective, low-cost photocatalyst. In addition to its photocatalytic performance, HTCC also offers good biocompatibility, making it well-suited for Photobiocatalytic applications. Chan et al. constructed an organic-microbe hybrid system based on HTCC for enhanced hydrogen production (Figure 4b).^54^ Under optimized conditions, a light intensity of 2000 W m^–2^ and an HTCC concentration of 1 g L^–1^, the hybrid system produced 1380 μmol of H_2_, marking a 35.3% enhancement in biological hydrogen production.

##### 3.3.5.1 Design Criteria

Select CDs with tunable LUMO levels and visible-light absorption were selected to match the redox potential of the target biocatalyst. Prioritize surface engineering (charge, size, and functional groups) to promote strong electrostatic or covalent coupling and enable efficient direct electron transfer. Avoid systems with high trap-state density or dominant fast fluorescence decay that limit long-lived charge carriers. Ensure structural stability and biocompatibility and consider covalent conjugation strategies when precise electron-transfer pathways are required.

### 3.4 Graphene-Based Materials

While the previous section highlighted carbon-based nanomaterials with quasi-zero-dimensional architectures and tunable surface chemistries that facilitate light harvesting and interfacial electron mediation, it is equally important to consider carbon allotropes with extended π-conjugated frameworks and long-range charge transport characteristics. In contrast to nanodot-type materials, higher-dimensional carbon nanostructures provide continuous conductive pathways, high carrier mobility, and mechanically robust platforms for enzyme immobilization and biointerface engineering. Such features are particularly advantageous in biohybrid photoelectrochemical systems, where interfacial resistance is minimized, direct electron transfer is enhanced, and stabilization of immobilized biocatalysts. Moreover, extended carbon networks offer opportunities for rational band-structure modulation, surface functionalization, and electrostatic tuning, thereby enabling an improved energy-level alignment between photosensitizers and enzymatic active sites. These properties are especially relevant when designing architectures that aim to suppress recombination losses and promote efficient charge separation under illumination. Accordingly, graphene-based materials represent a different yet complementary class of carbon scaffolds that emphasize conductivity, structural integration, and electrode interfacing over purely dispersible photocatalytic behavior. Their dimensionality and electronic delocalization allow not only enhanced charge transport but also controlled bionano interactions through covalent modification or noncovalent π–π and electrostatic coupling. Therefore, moving from carbon dots and related nanostructures to graphene reflects a logical progression from localized light-harvesting mediators toward conductive frameworks engineered for integrated biohybrid assembly. Graphene is a two-dimensional material composed of sp^2^-hybridized carbon atoms arranged in a hexagonal (honeycomb lattice). Graphene and its chemically converted derivatives (CCG) are well-known materials with exceptional electrical conductivity, material strength and high surface area with a large number of surface-active sites, making them highly attractive for applications in semiartificial photosynthesis (Figure 6).^117–120^ Although pristine graphene exhibits limited visible-light absorption and is not an efficient photosensitizer on its own, its oxidized forms may display semiconducting behavior. More importantly, graphene can serve as an effective electron-accepting and charge-transporting matrix when hybridized with photoactive chromophores. In such hybrid systems, the chromophore acts as the primary light absorber, while graphene functions as a conductive scaffold that facilitates rapid photoinduced electron transfer. The disturbance of graphene’s π-conjugation leads to a rise of a bandgap, thereby facilitating the photoinduced energy/charge transfer from the chromophore to the graphene matrix.

**Figure 6:**
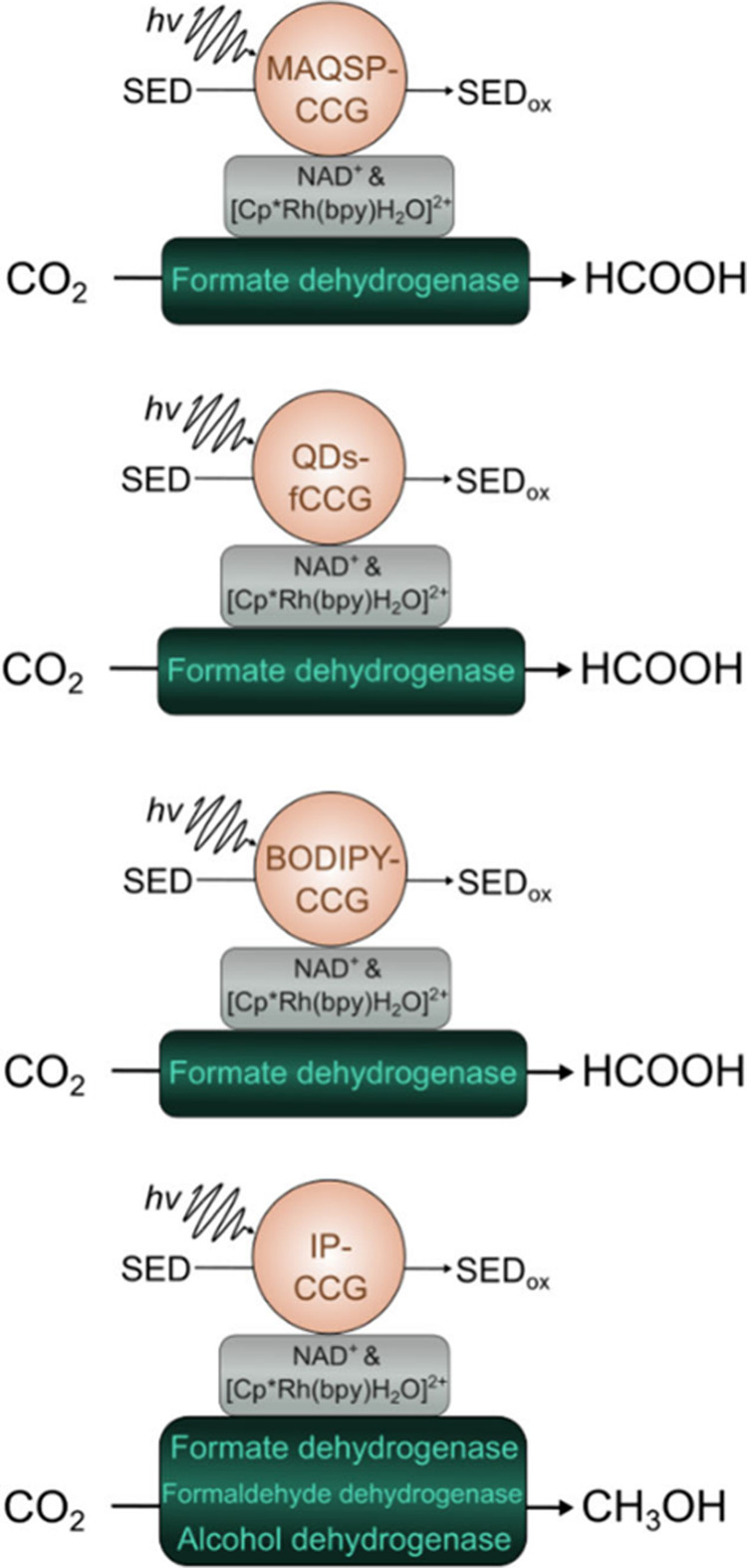
Biohybrid assemblies based on functionalized chemically converted graphene (CCG) and the reactions that they drive.

Yadav et al. reported a biohybrid assembly composed of chemically converted-graphene with multianthraquinone-substituted porphyrin as a photocatalyst and formate dehydrogenase (FDH) as a biocatalyst. Electron transfer from the porphyrin to graphene was facilitated by the well-matched energy levels between the porphyrin’s LUMO and graphene’s Fermi level that finally resulted in successful CO_2_ reduction to formic acid in the presence of a molecular redox mediator.^117^ In the follow-up study, they additionally modified graphene with a chromophore with trisubstituted isatin on the porphyrin rings. The resulting hybrid could drive multielectron CO_2_ reduction to methanol in the presence of a redox mediator and a multienzyme cascade involving FDH, formaldehyde dehydrogenase (FaldDH) and alcohol dehydrogenase (ADH).^120^ In a few years the same group reported a functionalized graphene quantum dots system integrated with FDH that showed much greater enhancement of both NADH photoregeneration and the production yield of formic acid from CO_2_.^119^ However, challenges such as band gap engineering of graphene and optimization of its energy level alignment with biocatalysts remain areas of active research.

#### 3.4.1 Design Criteria

Effective graphene-based systems should be engineered to exhibit a bandgap of ∼1.55–3.1 eV (corresponding to absorption of 400–800 nm) and a Fermi level aligned within 0.2–0.3 V of the target enzymatic redox potential, typically achieved through chromophore hybridization or heteroatom doping to enable efficient charge transfer. Concurrently, surface charge (|ζ| ≥ 20–30 mV) and specific anchoring functionalities must be tuned to control enzyme orientation and promote direct electron transfer with low overpotential (<100 mV). In contrast, the use of pristine graphene without optical or interfacial modification should be avoided, as its poor visible-light absorption and weak biocatalyst coupling generally result in low photocatalytic performance.

### 3.5 Organic Aggregation Nanoparticles

#### 3.5.1 Polymer Nanoparticles

Organic semiconducting polymers are a class of materials with extended π-conjugated backbones and delocalized electronic structures that grant them tunable optical and electronic properties to meet the biocatalyst needs.^121–123^ One challenge, particularly in applications such as semiartificial photosynthesis, lies in the lack of water solubility or dispersibility of many bulk conjugated polymers, which in turn restricts the availability of their accessible surface area. Since the surface area is one of the factors that significantly affects photocatalytic efficiency, significant effort has focused on designing conjugated polymer materials that maximize this parameter. One effective strategy involves leveraging the inherent hydrophobic nature of conjugated polymers to promote self-assembly into water-dispersible polymer nanoparticles (PNPs). By preparing into PNPs, the aggregate can assemble a series of multifunctional organic components into a closely packed particle, which is beyond of their single component counterpart, e.g., monomer in solution state. A notable subclass of these are polymer dots (Pdots), which are defined by their small particle sizes, typically less than 100 nm.^124,125^ Both PNPs and Pdots have garnered increasing attention across the chemical, biological, and materials science disciplines.^26,47,57,58,60,126–132^ Over the past few years, polymer-based biohybrid assemblies have demonstrated the ability to successfully produce a range of valuable compounds (Figure 7), including hydrogen,^31^ oxygen,^131,132^ ammonia,^60^ poly­(3-hydroxybutyrate) (PHB),^57,133^ acetic acid,^58^ 2-oxybutyrate,^130^ γ-polyglutamic acid (γ-PGA) and bacitracin A.^47^

**Figure 7:**
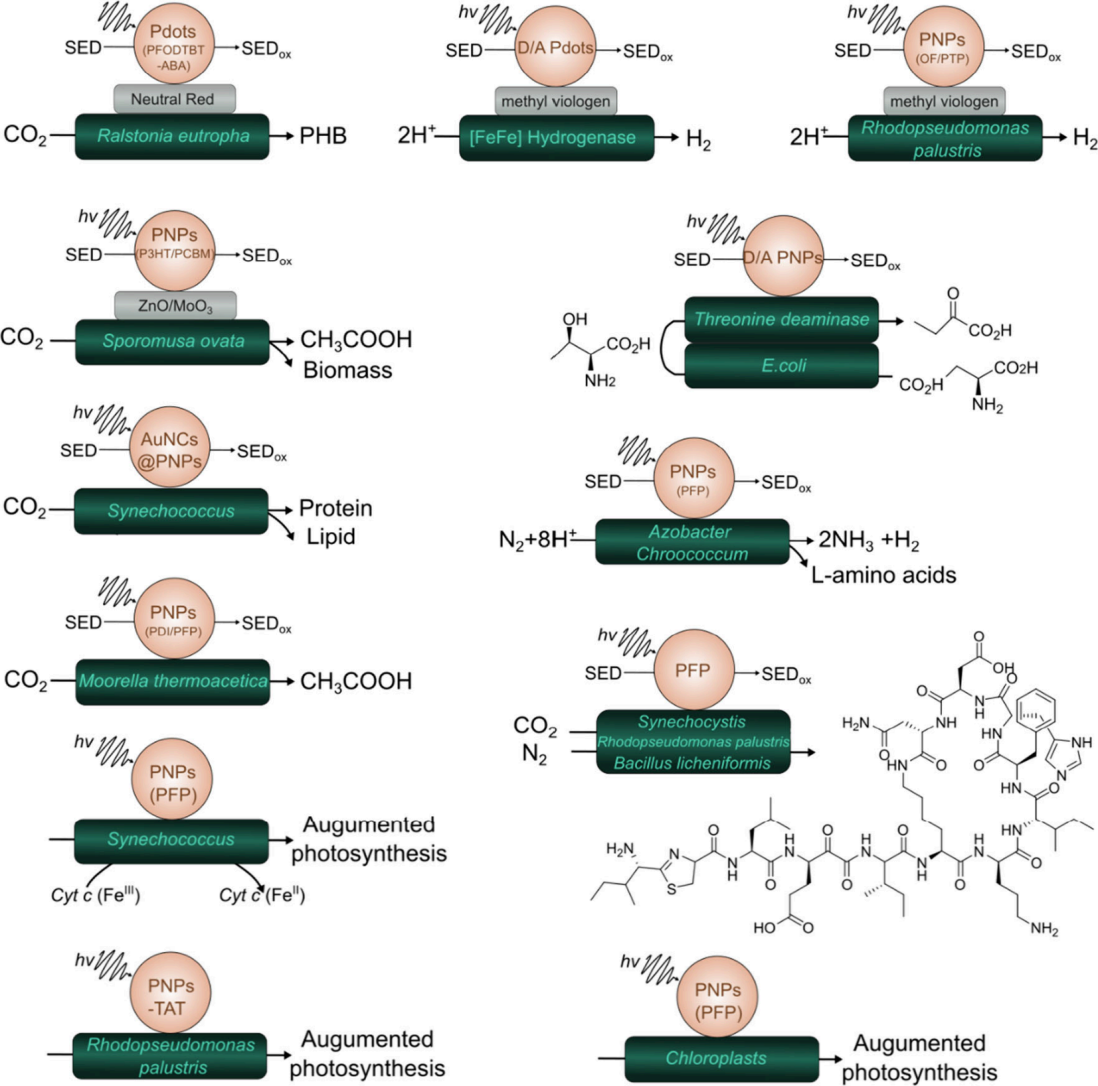
Photobiocatalytic transformations utilizing PNPs.^31,47,57,58,60,126–138^

Key features that make PNPs especially promising for the design of advanced biohybrid platforms and related bioconversion technologies include tunable electronic properties, high photostability, biocompatibility, structural versatility, and efficient surface functionalization. In particular, the structural versatility of these polymers, achieved through chemical modification of their relatively rigid backbones, provides the basis for tailoring electronic properties and enabling diverse surface functionalization strategies, thereby directly supporting their performance in biohybrid assemblies.^134,135^ To enhance the performance of polymer-based biohybrid assemblies, various structural modifications of organic semiconducting polymers have been explored, as discussed in the following section.

##### 3.5.1.1 Unary Polymer Nanoparticles

Unary polymer NPs are defined here as NPs that are contrary to binary polymers and composed of a unary polymer, which may contain donor and acceptor building blocks. Several unary polymer-based PNPs with for example fluorene units in their backbones (e.g., PFP and PFBT) have been used to promote the natural photosynthetic efficiency of photosynthetic organisms.^128^ Qi et al. developed a synthetic method for in situ polymer preparation on the surface of living cells using a palladium-catalyzed cross-coupling reaction. This approach led to the creation of a biohybrid assembly that enhanced ATP production in *Chlorella pyrenoidosa* by broadening light absorption and accelerating the algae’s cyclic electron transport.^136^ Méhes et al. designed a composite of nanocellulose and the PEDOT-based conducting polymer system PEDOT:PSS (poly­(3,4-ethylenedioxythiophene):polystyrene sulfonate) as the anode in biohybrid cells that included thylakoid membranes and redox mediators in solution to enhance energy conversion efficiency.^137^

Functionalization of PNPs is seen as a straightforward approach to facilitate electrostatic interaction between abiotic and biotic components. This can be achieved either through (I) the addition of specific functional groups to the polymer backbone or (II) through the addition of charged surfactants during the PNPs preparation. Yet, these approaches have some challenges such as synthetic complexity in (I), or the formation of a dense surfactant shell around the NPs’ surface that could sterically limit DET in biohybrid systems in (II). Efficient energy transfer and close interaction with *Chlorella pyrenoidosa* through the cationic quaternary ammonium groups embedded in the PBF polymer backbone or PPE polymer backbone favored the enhanced system activity through approach I.^129,136^ Following approach II, Yu et al. functionalized Pdots with a positively charged surfactant to achieve efficient PHB production with the help of *Cupriavidus necator (formerly* *Ralstonia eutropha* *)* H16.^57^ Recently a few more reports on the utilization of both electrostatic immobilization approaches for the generation of biohybrid assemblies appeared, consequently allowing the reduction of N_2_ (Figure 8),^60^ and the formation of value-added chemicals, such as PHB,^57,133^ γ-polyglutamic acid (γ-PGA) and the synthesis of bacitracin A.^47^

**Figure 8:**
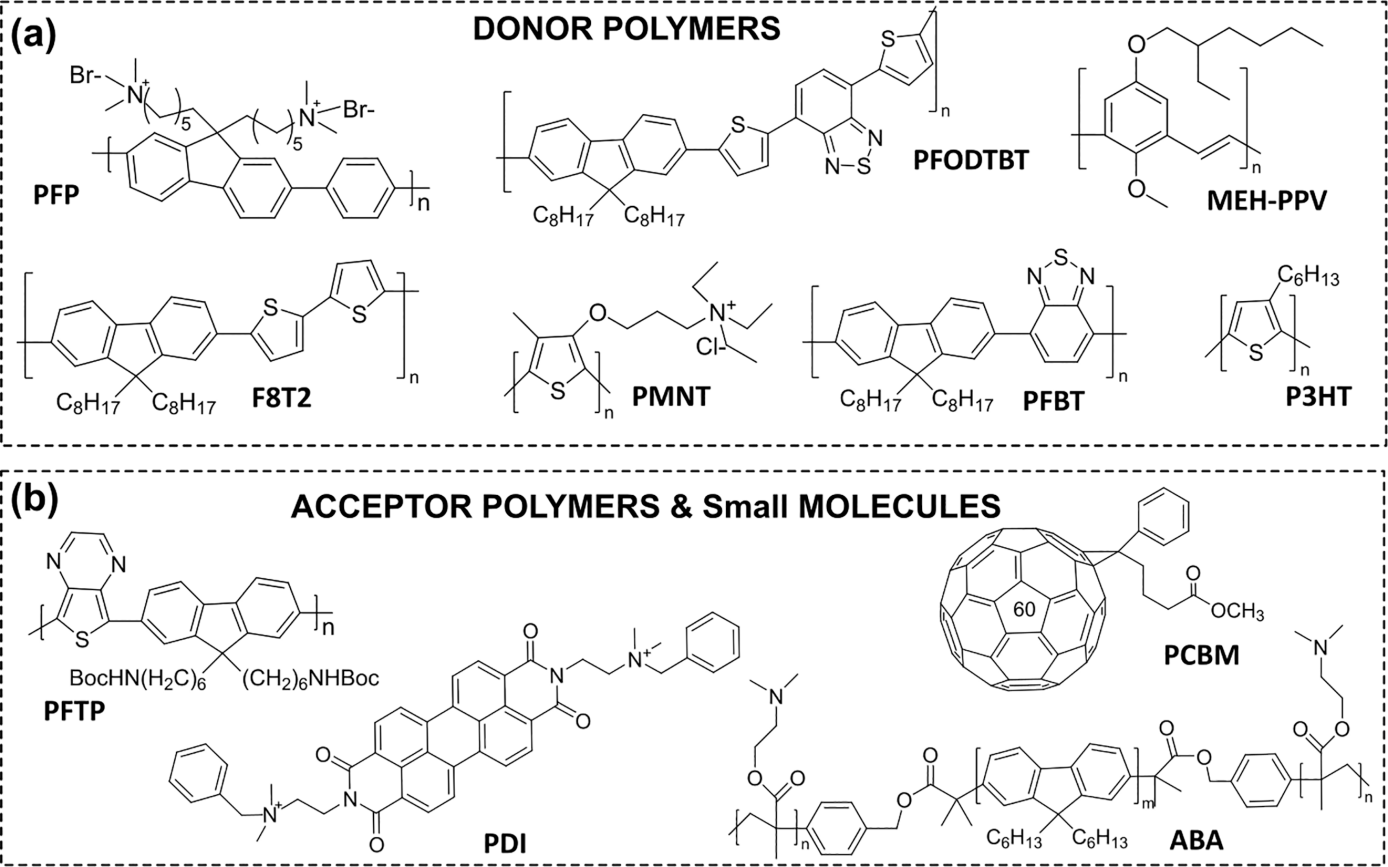
Corresponding structures of utilized donor polymers (a) and acceptor polymers and small molecules (b) used in PNPs.

##### 3.5.1.2 Heterojunction Polymer Nanoparticles

One of the main challenges that limits the performance of most PNPs is poor charge generation. Upon photoexcitation, PNPs, which typically have low dielectric constants, generate electrostatically bound excited electrons and hole pairs (excitons), leading to weak charge separation and, consequently, severe recombination. Since the generation of free charge carriers is a key step in any redox reaction, organic semiconductors must first overcome the challenge of exciton dissociation in a low-dielectric environment to enhance their solar energy conversion efficiency. One of the most effective solutions to this problem is inspired by organic photovoltaic solar cells,^139^ where ultrafast exciton dissociation is achieved by promoting electron transfer along an energetically favorable path. In case of heterojunction NPs electron transfer occurs from conjugated donor polymers and either a conjugated acceptor polymer or small-molecule acceptors.^19^ Intermolecular electric fields between donor and acceptor components, driven by interfacial built-in field, are believed to facilitate subpicosecond, long-range charge separation. In addition to rapid separation, incorporating additional acceptor units also helps to generate long-lived charge carriers by charge hopping, which are less prevalent in single-donor PNPs.

The energy level alignment between donors and acceptors within the heterojunction system is critical to achieving effective charge separation and high energy conversion efficiency. Recent studies have demonstrated that heterojunction PNPs and Pdots outperform their single-polymer counterparts in photocatalytic applications, due to enhanced light absorption and more efficient charge separation.^31^ Pavliuk et al. studied the photophysical properties of heterojunction NPs composed of F8T2 (P1) and ABA (P2) polymers used to drive [FeFe]-Hydrogenase for solar-driven hydrogen evolution (Figure 9a). Efficient subpicosecond charge separation at the donor/acceptor interface and > 1.6 ns long-lived charge separated state led to much higher hydrogen evolution yields than the corresponding single-polymer NPs.^31^ Gai et al. designed a heterojunction PNPs composed of PFP and PDI polymers with suitable energy levels to directly inject electrons into the redox mediators rubredoxins (Rd) and flavoproteins (Fp) located on the surface of *Moorella thermoacetica* bacterial membrane.^58^ The resulting biohybrid assembly could efficiently convert CO_2_ to acetic acid (Figure 9b) under light illumination with an efficiency of 1.6%, which is comparable to that of reported inorganic biohybrid systems. In order to enhance the production of hydrogen by *Rhodopseudomonas palustris*, Wang et al. coupled two single polymers (oligofluorene and polythiophene) with efficient fluorescence resonance energy transfer (FRET) to the microorganism.^127^ Furthermore, Yu et al. presented a heterojunction PNP using MEH-PPV as an electron donor polymer and PFTP as an electron acceptor polymer. Threonine deaminase was covalently attached to build a ternary biohybrid system for the generation of 2-oxybutyrate.^130^

**Figure 9:**
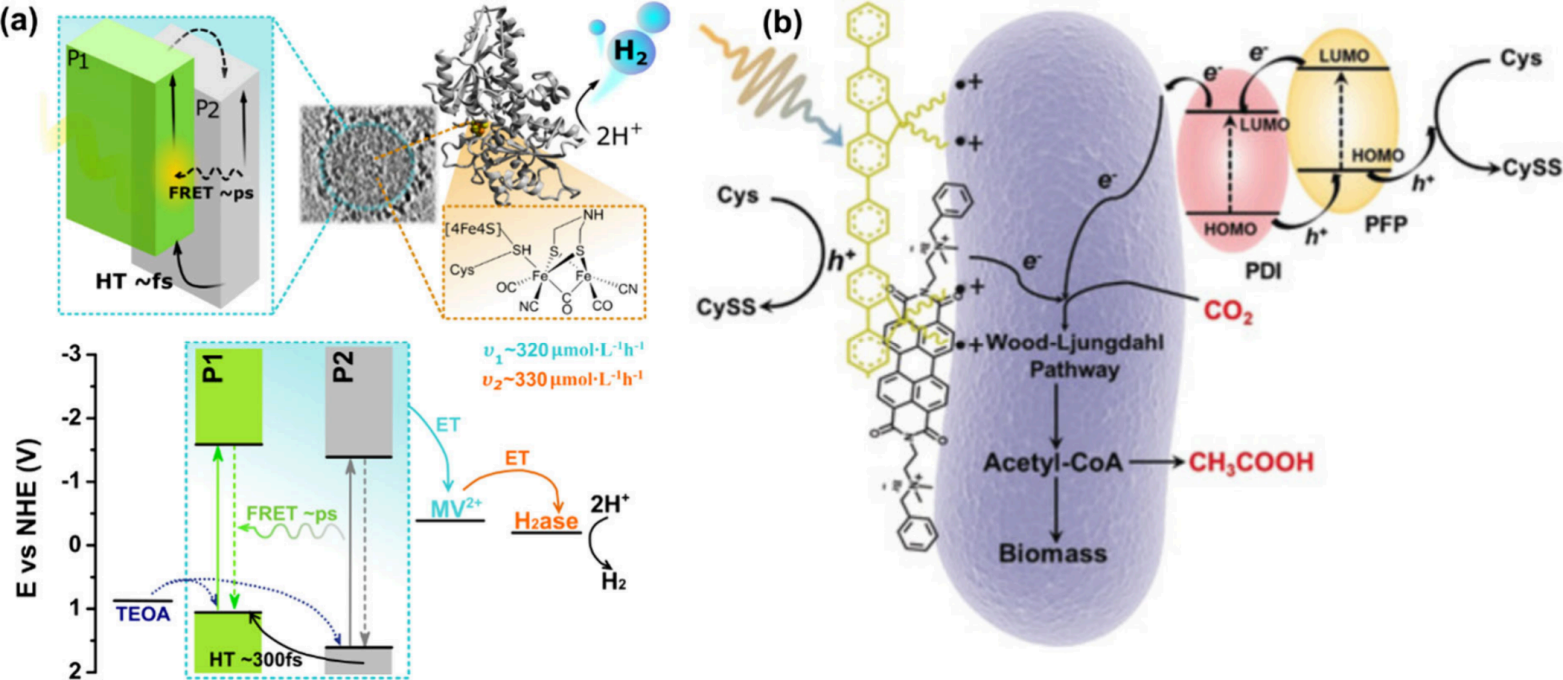
(a) Energy diagram summarizing the photophysical processes involved in a biohybrid assembly composed of heterojunction P1/P2 Pdots and [FeFe]-hydrogenase from *Chlamydomonas reinhardtii* during photocatalysis (where ET-electron transfer, HT-hole transfer, curved arrows represent charge transfer processes, and P1 - F8T2, and P2 -P2 -ABA. Reproduced from ref 31. Copyright (2022) American Chemical Society. (b) Diagram of the PDI/PFP/*M. thermoacetica* photosynthesis hybrid system used for the reduction of CO_2_ to acetic acid. Reproduced with permission from ref 58. Copyright (2020) Wiley.

In some cases, the donor/acceptor heterojunction PNPs utilize not only organic components but also a blend of organic and inorganic materials. Cong et al. synthesized an innovative heterojunction system combining gold nanoclusters with organic semiconductors (AuNC@OFTF), by covalently linking the amino groups of the conjugated OFTF to the carboxyl groups of MUA ligands on pristine AuNCs. This heterostructure featured prolonged excited-state lifetimes and efficient charge separation (Figure 10). Once internalized into cyanobacteria, the hybrid material bound closely to the electron transport chain, enabling direct involvement of photogenerated electrons in PSII activity and improving the PHB yield, along with enhanced protein and lipid production. The strategy shows strong potential for broadly activating microbial metabolism for complex product synthesis while minimizing energy losses typically associated with photoelectron transfer across membranes.^138^

**Figure 10:**
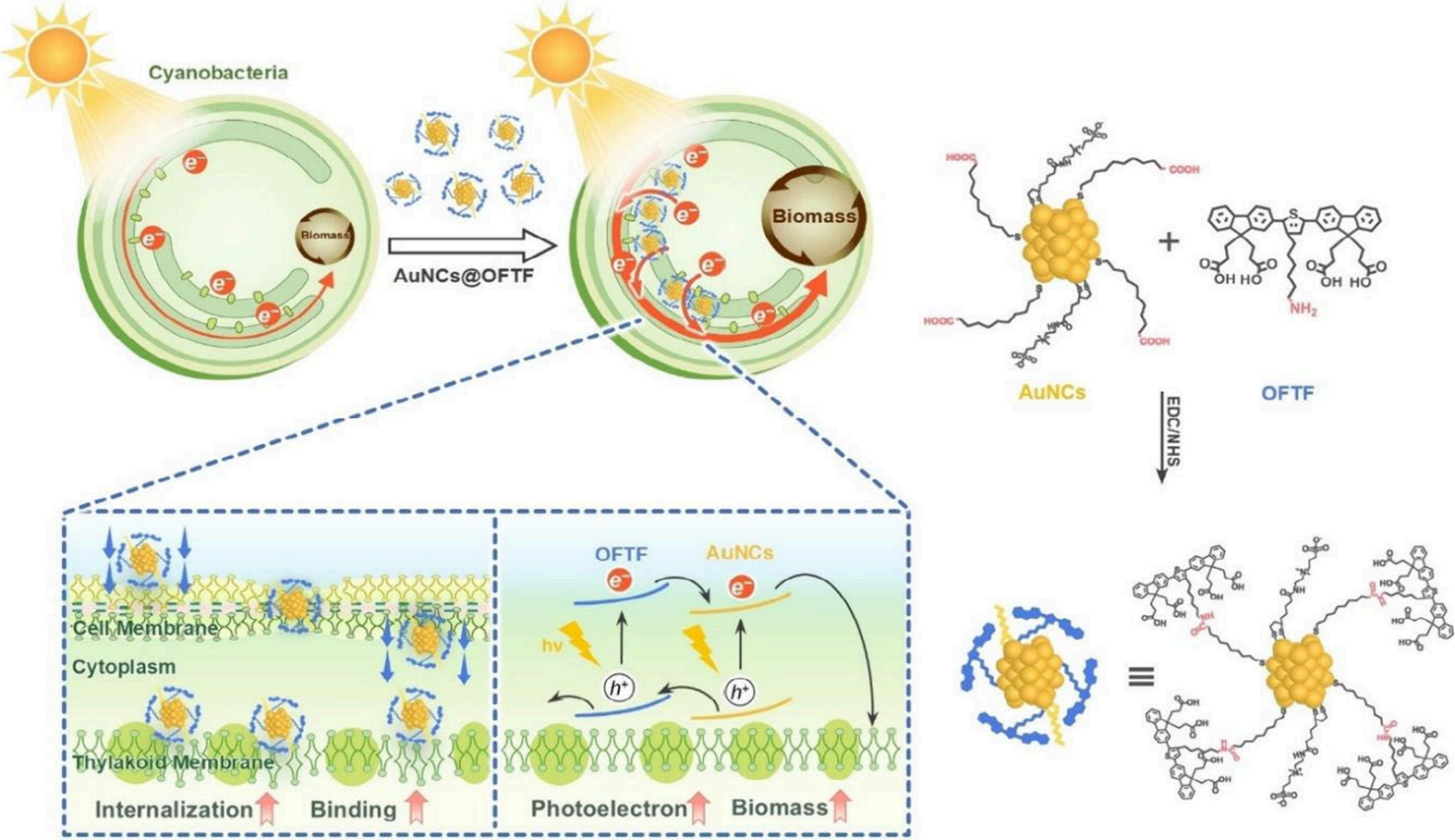
Schematic diagram of AuNCs@OFTF entering cyanobacteria for enhanced biomass production. Reproduced with permission from ref 138. Copyright (2024) Wiley.

To further enhance electron–hole separation in heterojunction PNPs, an alternative approach has been developed. Wen et al. introduced a ZnO electron transport layer and a MoO_3_ hole transport layer on either side of P3HT:PCBM organic semiconductor films, creating a biocompatible photocatalyst with an ITO/ZnO/P3HT:PCBM/MoO_3_ multilayer structure. This carefully aligned energy level configuration enabled efficient energy transfer from the active layer to microbial sites, improving the overall performance.^140^

#### 3.5.2 Organic Molecule Nanoparticles

Besides polymer nanoparticles, organic small molecule nanoparticles have also recently been employed in biohybrid photocatalytic systems.^24,43^ Compared to polymers, organic small molecules possess well-defined structures, and their absorption properties and energy levels can be feasibly tuned by modifying different structural units, such as donor, acceptor, and π-linker components.^141–145^ Similar to polymers, these small molecules typically require a large π-conjugated system to extend their optical absorption into the visible-light region. This often leads to solubility or dispersibility challenges in aqueous environments, unless specific hydrophilic units are introduced.

Forming nanoparticles of these molecules in the presence of surfactants can address the issue of water dispersibility while also allowing control over surface charge to facilitate coupling with biocatalysts. Moreover, appropriate molecular packing (for instance, through J-aggregation) can red-shift the absorption spectrum, providing an important advantage of molecular nanoparticles. By using diphenylalanine (FF) as a building block capable of forming various nanostructures and tetra­(p-hydroxyphenyl)­porphyrin (THPP), Kim et al. synthesized efficient light harvesting hybrids (FF-THPP) for a redox enzymatic reaction using glutamate dehydrogenase. Supramolecular FF-THPP hybrids with J-aggregation showed much higher conversion efficiency than free THPP light harvesters, due to the designed directional morphology of the scaffold material that facilitated electron collection and transport to the biocatalyst (Figure 11a).^43^ Pavliuk et al. have synthesized small organic phenoxazine dye based nanorods to build a biohybrid assembly with carbon monoxide dehydrogenase (CODH) as a biocatalyst (Figure 11). They showed that functionalization of organic nanorods with suitable surfactant could facilitate the intimate interaction and product formation.^24^

**Figure 11:**
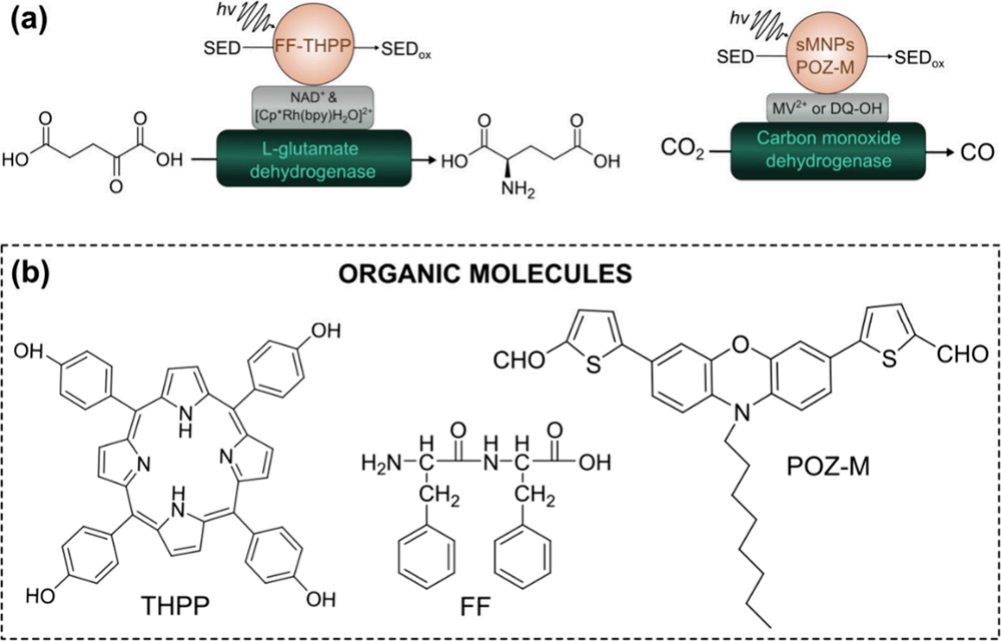
(a) Photobiocatalytic transformations driven by organic molecule NPs-based biohybrid assemblies and (b) and the structures of corresponding molecules.^24,43^

##### 3.5.2.1 Design Criteria

Design polymer or molecular nanoparticles with energetically matched donor–acceptor components promotes efficient exciton dissociation and long-lived charge separation, particularly in heterojunction architectures. Prioritize surface functionalization strategies that enable close electrostatic or covalent coupling to biocatalysts without forming insulating surfactant shells that hinder direct electron transfer. Avoid single-component systems with poor dielectric properties and severe recombination losses unless compensated for by built-in internal electric fields. Ensure aqueous dispersibility, photostability, and scalable synthetic routes while maintaining precise energy-level alignment for targeted redox transformations.

### 3.6 Carbon Nitrides

Graphitic carbon nitride (g-CN_x_ or g-C_3_N_4_) is a metal-free polymeric semiconductor composed mainly of carbon and nitrogen atoms arranged in a layered graphite-like structure. It has a moderate band gap of approximately 2.7 eV, allowing it to efficiently absorb light and generate photoexcited electrons and holes; and its band positions are suitable for driving various reactions, such as water splitting. Because of its chemical robustness and high specific surface area, g-CN_x_ is particularly appealing for integration into biological systems. However,, the application of g-CN_x_ in biohybrid assemblies is often limited by its poor dispersion in water, and weak interactions with electron transfer partners such as redox mediators, microorganisms and enzymes.^32^ Caputo et al. reported a g-CN_x_ and [NiFeSe]-hydrogenase system with biotic-abiotic interactions that relied on the utilization of a redox mediator (MV^2+^), and that only poor direct electron transfer was observed in a mediator-free system. g-CN_x_ often has limited visible-light absorption, and by relying on only UV-light, it is more likely to contribute to photodamage of biocatalysts.^32^ Currently, g-CN_x_ has found application in driving hydrogen evolution,^32,33^ PHB production,^146^ water splitting and CO_2_ reduction (Figure 12).^147^

**Figure 12:**
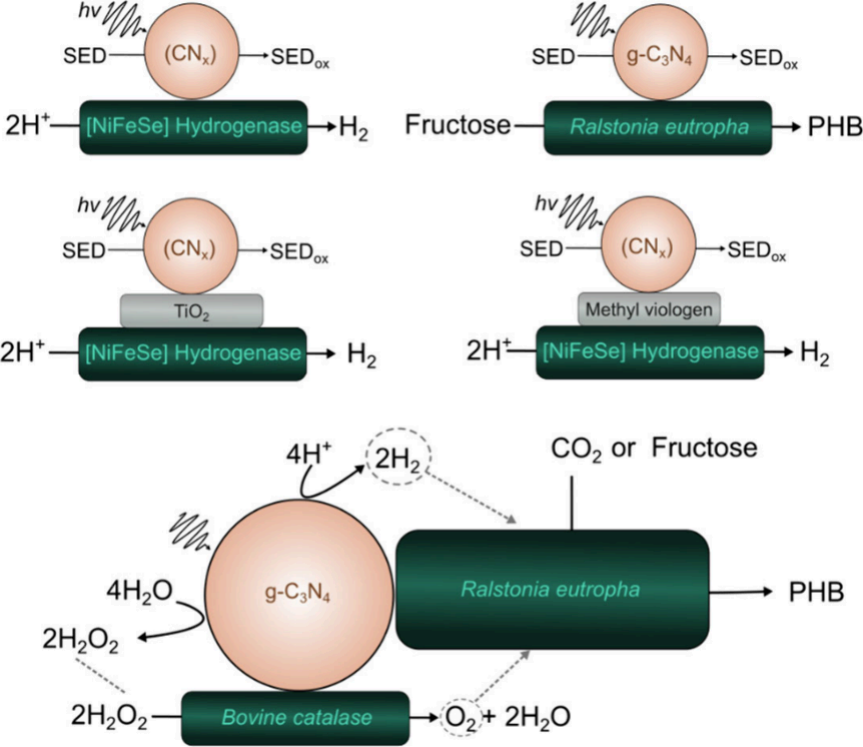
Photobiocatalytic reactions driven by carbon nitride biohybrid assemblies.^32,33,146–148^

Recent strategies to improve g-CN_x_ performance and enhance electron transfer efficiency include surface functionalization, nanostructuring, and hybridization with other materials (e.g., carbon dots or TiO_2_).^33,149^ By hybridizing CN_x_ with TiO_2_ (Figure 13) a slightly broader spectral absorption and more efficient charge transfer were achieved via photoinduced electron transfer from the CN_x_ LUMO to the conduction band of TiO_2_ and *via* direct optical charge transfer from the HOMO of CN_x_ to the conduction band of TiO_2_, extending light absorption up to ∼540 nm.^33^ Similarly, Xu et al. decorated nanoporous g-C_3_N_4_ with graphene oxide quantum dots to achieve improved visible-light harvesting ability and higher charge transfer efficiency. However, in their case, the designed hybrid system deactivated about 99.6% of *E. coli* cells within 4 h under visible light irradiation, indicating poor biocompatibility of CN_x_.^148^ Tremblay et al. designed a novel g-C_3_N_4_ -catalase system that was employed to further build a hybrid photosynthesis system, where the production of PHB from either CO_2_ or fructose by *R. eutropha* was driven by water-splitting photocatalysis. The authors highlighted the necessity of a comprehensive investigation of the electron transfer mechanisms to fully confirm the suggested electron transfer pathways.^147^

**Figure 13:**
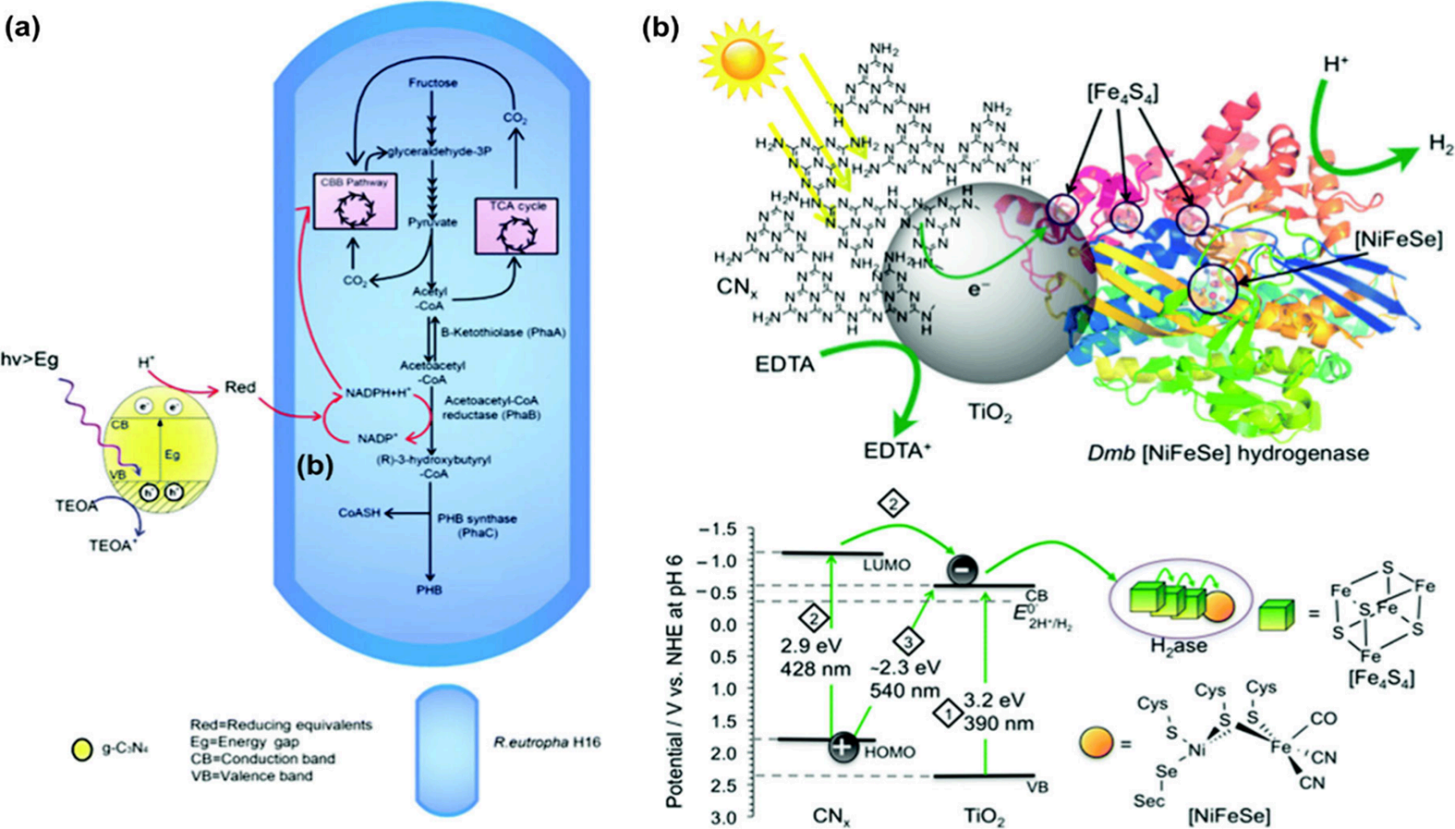
(a) Scheme of a biohybrid system based on g-C_3_N_4_ and fructose-fed *R. eutropha* for PHB production. Reproduced with permission from ref 146. Copyright (2019) Royal Society of Chemistry; (b) Schematic representation of light-driven H_2_ production with *Dmb* [NiFeSe]-H_2_ase (PDB ID : 1CC1) on CN_*x*_–TiO_2_, and the corresponding energy diagram that represents the occurring processes. Reproduced from ref 33. Copyright (2015) American Chemical Society.

#### 3.6.1 Design Criteria

Prioritize surface functionalization and hybridization (e.g., with CDs or TiO_2_) to improve water dispersibility, broaden visible-light absorption, and enhance charge transfer to biocatalysts. Avoid relying solely on UV-active g-CN_x_ to prevent photodamage to cells or enzymes. Ensure energy-level alignment with the target redox partners and consider mediator-assisted electron transfer when direct DET is inefficient. Balance chemical robustness with biocompatibility to maintain activity without harming the biological component.

### 3.7 Other Organic Photosensitizers

#### 3.7.1 Covalent–Organic Frameworks

are a class of crystalline, porous polymers formed by the covalent linkage of organic building blocks into well-defined, extended two- or three-dimensional networks. Yadav et al. designed a two-dimensional triazine based covalent–organic frameworks (COFs) (2D COF) *via* the condensation–polymerization of cyanuric chloride and perylene diimide.^150^ The resulting 2D COF contained highly ordered stacked columnar π-electron channels that facilitate the efficient transfer of photoexcited electrons in the stacking direction. Compared to monomeric perylenediimide, the COF film demonstrated an enhanced NADH photoregeneration yield when using ascorbic acid as the sacrificial electron donor. Furthermore, the two-dimensional COF film exhibited strong conjugation with FDH and enabled efficient CO_2_ conversion to formate (Figure 14a).

**Figure 14:**
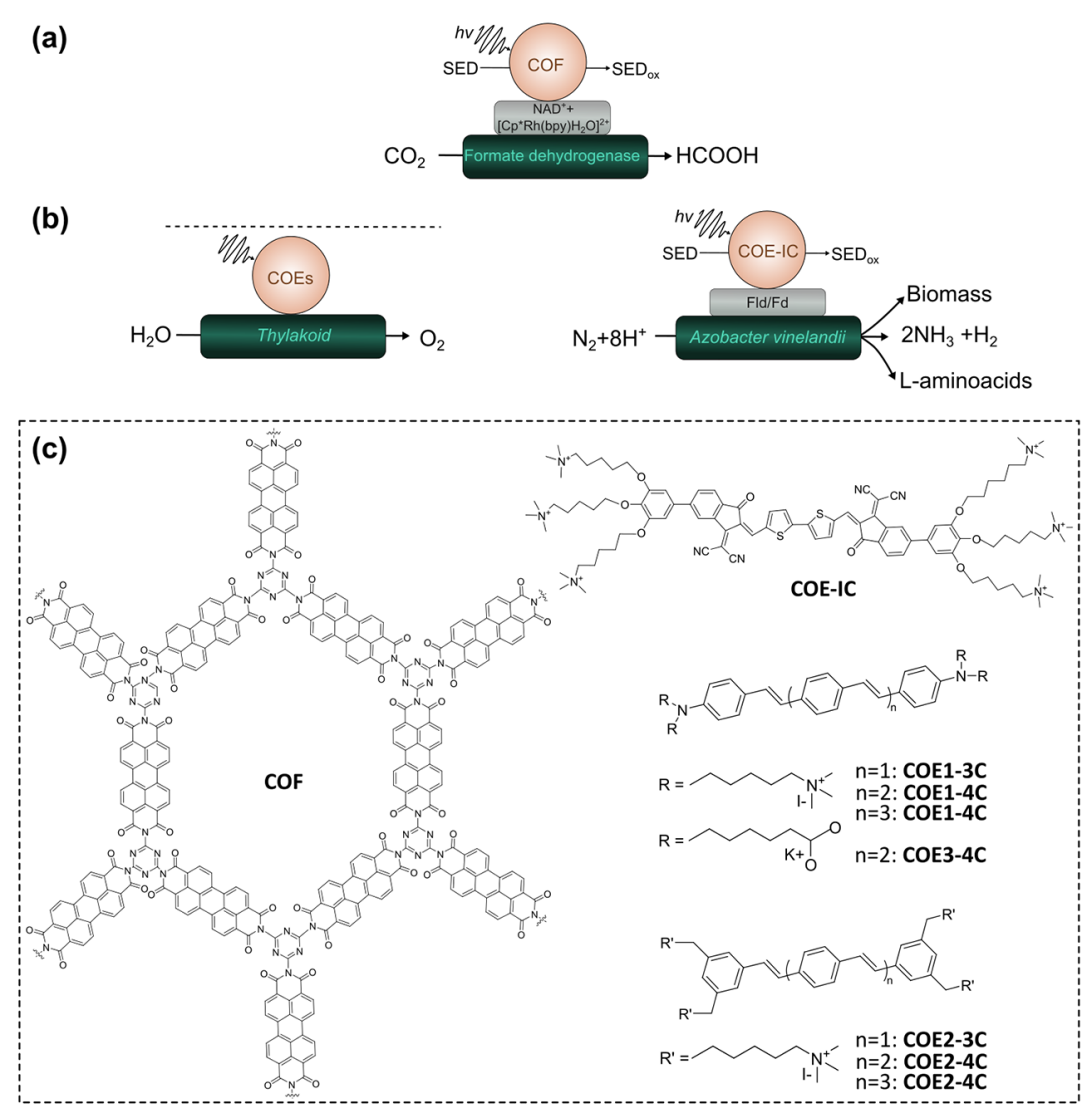
Photobiocatalytic transformations driven by biohybrid assemblies based on (a) covalent–organic framework (COF),^150^ (b) conjugated oligoelectrolytes (COEs),^26,151^ and (c) their corresponding structures. Note that the structure of the COFs is more complex than depicted here.

#### 3.7.2 Conjugated Oligoelectrolytes

similar to conjugated polymers, they are defined as π-conjugated materials with alternating single and double bonds that create a delocalized electron system and additionally contain polar ionic pendant groups. In contrast to conventional polymers, conjugated oligoelectrolytes (COEs) contain chemically defined structures with a defined number of repeating units (usually 3–6 units).^44^ COEs’ pendant ionic side groups are generally used to improve solubility and interactions with biocatalysts.^152^ Here we discuss COEs in light-driven catalysis, and refer the reader to other reviews for their use in bioelectrocatalysis.^44^ In 2015 Bazan group reported the utilization of COEs in thylakoid-membrane-based photobioelectrochemical devices.^151^ They investigated the impact of the repeating unit length, the charge of the ionic side groups, and the overall optoelectronic properties of COEs on photocurrent generation and interfacial electron transfer. In 2023, the same group reported the utilization of COEs with an acceptor–donor–acceptor type conjugated core for photocatalytic nitrogen fixation to ammonia, L-amino acids and biomass (Figure 14b).^26^

##### 3.7.2.1 Design Criteria

Select COFs or COEs with well-ordered π-conjugated channels promote directional electron transfer and efficient coupling with biocatalysts. Use ionic or polar side groups in COEs to enhance solubility and interfacial interactions but avoid excessive steric hindrance that can block electron transfer. Prioritize materials with tunable optoelectronic properties for energy-level matching with target enzymes or redox partners. Consider the balance between structural complexity and synthetic accessibility to maximize the performance without introducing unnecessary preparation challenges.

## 4 Redox Mediators as Tools for Activating Biocatalysts

To enable efficient electron transfer between an organic photosensitizer and a biocatalyst, two strategies can be employed, as mentioned before: DET and MET. A biohybrid system requiring a redox mediator to shuttle the charges to biocatalyst can be treated as MET. Without the need of a redox mediator, the biohybrid system can be considered as a DET. Specially, whole-cell systems are inherently complex, and apparent DET behavior may involve mixed or parallel pathways, such as electron transfer via endogenous redox-active metabolites or accelerated membrane electron transfer chains.^153,154^ In this review, we treat all systems without a need for additional redox mediator as DET. DET offers significant advantages, such as process simplicity, cost efficiency and the avoidance of potentially cytotoxic redox mediators (e.g., the commonly used MV^2+^) which may also undergo unwanted competitive light absorption or cause cross-reactivity with intermediates in the reaction medium.^4^ However, DET presents a greater challenge, as it often requires direct physical contact and precise orientation between the active site of the biocatalyst and the photosensitizer as well as photosensitizers with long-lived excited states to ensure efficient charge transfer. As discussed in Section 3, many organic photosensitizers have short-lived excited states or cannot transfer electrons directly to the biocatalyst, often due to encapsulation within dense surfactant shells, collectively posing a major challenge for developing biohybrid systems based on organic-materials. The use of electron mediators in MET offers a practical solution by facilitating electron transfer between the source of photoexcited electrons and the biocatalyst’s active site, even when the biocatalyst is poorly oriented or weakly coupled with the photosensitizer. Additionally, redox mediators may protect enzymes from the deactivation at highly reducing potentials of light harvester.^76,155^ In Section 4 we discuss organic redox mediators that have been used to enhance electron transfer between organic photosensitizers and biocatalysts.

Three types of organic redox mediators have been used in biohybrid assemblies with organic photosensitizers: molecular, protein-based, and polymeric. Table 3 lists representative examples with their structures, redox potentials, common OPS pairings, and key advantages and limitations.

**Table 3:**
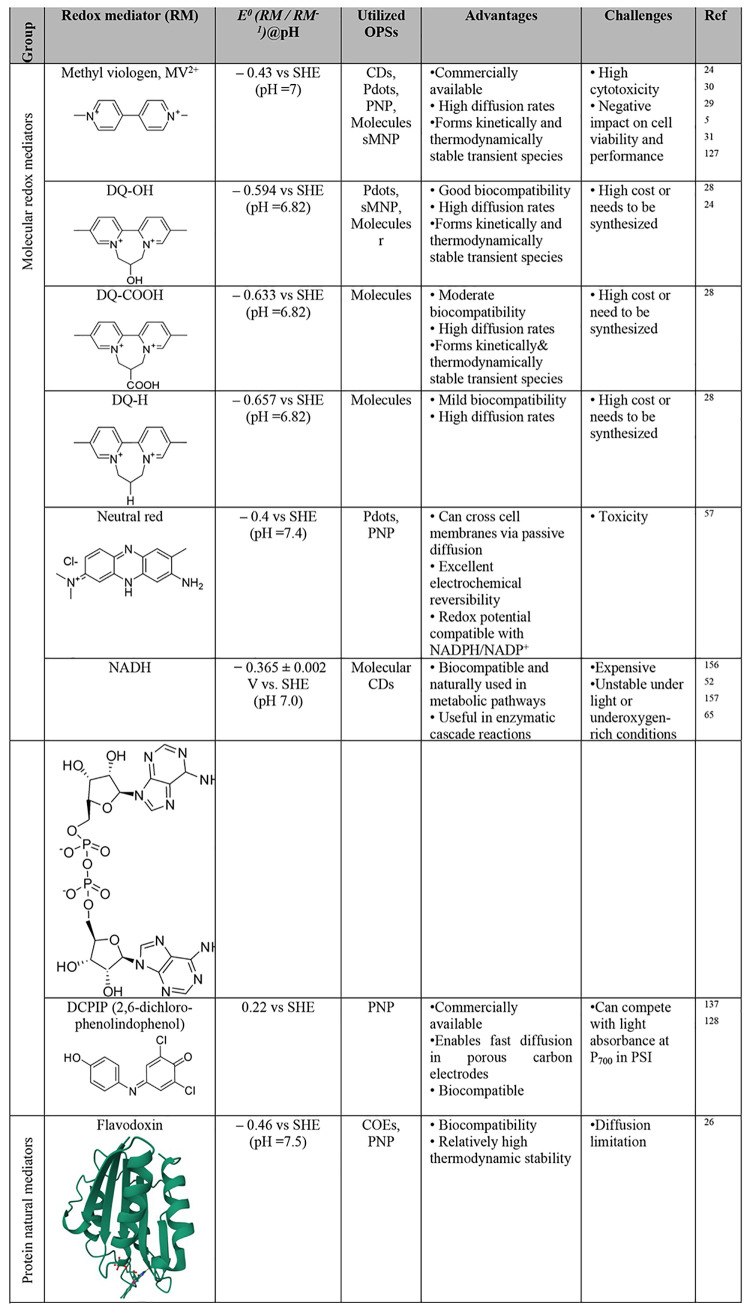

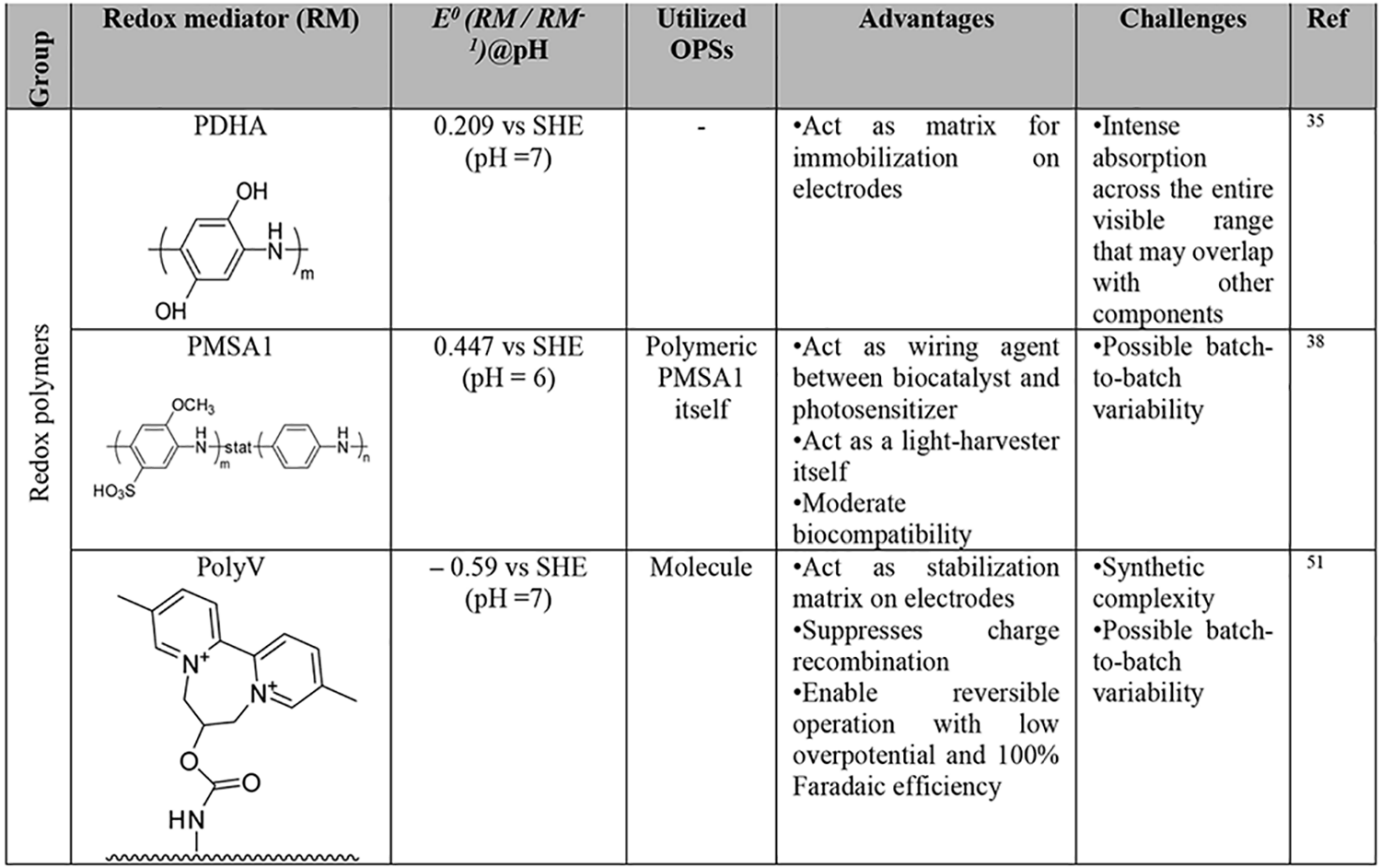
Structures and Properties of Redox Mediators and Scaffolds Used with Organic Photosensitizers^156,157^

### 4.1 Common Redox Mediators

#### 4.1.1 Molecular Redox Mediators

Molecular redox mediators are defined as small, diffusible molecules that shuttle electrons between a photosensitizer and a biocatalyst in biohybrid systems. These mediators are widely used due to their chemically tunable redox potentials, fast and reversible electron transfer kinetics, high solubility, and good compatibility with various organic photosensitizers and biocatalysts. Molecular redox mediators are commonly classified into two categories: natural cofactors and synthetic (artificial) mediators. One of the natural redox mediators essential for enzymatic catalysis, particularly for oxidoreductases, is the nicotinamide adenine dinucleotide cofactor [NAD­(P)­H], which mediates the transfer of two electrons and one proton.^4^ However, the high cost and demanding regeneration of [NAD­(P)­H] often limit its utilization in biohybrid systems. Common examples of synthetic molecular mediators include bipyridinium cations, various transition metal complexes, *etc* (Table 3). Among their challenges are high toxicity and the tendency to undergo side reactions, or photodegradation under extended light exposure. Moreover, the performance of molecular redox shuttle mediators strongly depends on the alignment of redox potentials with both the excited-state photosensitizer and the redox-active site of the biocatalyst, as well as on the diffusion dynamics in the reaction medium.

***Bipyridinium cations***, such as viologens and diquats (DQs) have been extensively used as redox mediators in biohybrid systems due to their high solubility in water and their ability to form thermodynamically (normally around 300 mV for organic component) and kinetically stable transient species with lifetimes in the range of a few hundred of microseconds to minutes or even days under an inert atmosphere.^158–160^ High stability and long lifetimes of singly reduced diquats derivatives have been attributed to the steric reorganization of the pyridinium rings into a coplanar structure.^159^ By tuning the structure and molecular geometry of DQ derivatives, one can affect the reduction potentials and thus the driving forces for forward electron transfer from the aforementioned ionized OPSs. Gamache et al. investigated a range of redox mediators in an EY-based whole-cell system, such as MV^2+^, DQ, DQ-OH and DQ-COOH, (Table 3).^5,28^ It was found that redox mediators could suppress the photodegradation of the reductively quenched intermediate EY_red_ and dramatically increase the system performance and long-term stability (e.g., with DQ-OH). DQs with higher and more favorable driving forces for the electron transfer toward hydrogenase, as well as with more favorable kinetic effects (e.g., reorganization energy) showed significantly higher rates of product formation than when applying MV^2+^, provided that there is a sufficient reservoir of DQ^•+^- species.^5^ Cell viability studies revealed that MV^2+^ was highly toxic to cells even under anaerobic conditions, possibly due to the higher reactivity of MV^•+^ compared to DQ^•+^, or due to its ability to act as a reductant for cellular components (see Section 5.2 for details).^161^ In this regard, the lower reactivity of DQ^•+^ due to the structural rearrangement of the electron-withdrawing groups,^159^ was suggested to underlie the slightly higher biocompatibility of DQ derivatives.^5,28^ Furthermore, Pavliuk et al. compared the impact of MV^2+^ and DQ-OH mediators in powering a biohybrid assembly with a phenoxazine-based organic molecular nanorod photosensitizer and carbon monoxide dehydrogenase II from *Carboxydothermus hydrogenoformans* as the biocatalyst. DQ-OH with a more favorable reduction potential for CO_2_ reduction could contribute to the rate-limiting step of the system and improve both the kinetics (TOF ∼ 0.6 s^–1^), and the product selectivity as well as the product yield.^24^

***Alternative molecular redox mediators*** for organic-material-based semiartificial photosynthesis include neutral red and DCPIP (2,6-dichlorophenolindophenol).^57,128,137^ The interest in DCPIP mediators is attributed to their bio- and redox potential compatibility, e.g., with PSI enzymes, and to their frequent use in combination with ascorbate salts as sacrificial electron donors.^158,162^ However, a potential challenge in using DCPIP arises from its light absorption within the Q transition band of chlorophylls (650–750 nm), which may compete with photosynthetic light harvesting. Neutral red is a phenothiazine molecule, though it also exhibits competing light harvesting that can cross cell membranes and enter the cell through passive molecular diffusion. Yu et al. revealed that neutral red with redox potentials suitable for driving the Calvin cycle of *Ralstonia eutropha* H16, enabling the conversion of CO_2_ into PHB in the presence of Pdots.^57^

#### 4.1.2 Protein Natural Mediators

Protein-based redox mediators offer a biologically compatible alternative to synthetic molecular mediators. In organic-material-based semiartificial photosynthesis, commonly used examples include flavoproteins. These proteins can interact with both photosensitizers and biocatalysts via well-defined, often directional electron pathways, enhancing selectivity and minimizing unwanted side reactions, thus facilitating electron transfer. However, the integration of protein-based mediators into hybrid assemblies poses challenges in terms of stability, scalability, and structural integrity under photochemical conditions. For example, Chen et al. demonstrated that photoexcited COEs could thermodynamically drive electron transfer to carrier proteins, such as ferredoxin/flavodoxin, which subsequently delivered electrons to nitrogenase for N_2_ reduction.^26^

#### 4.1.3 Redox Polymers

Redox polymers are macromolecules that incorporate a polymer backbone, redox-active functional groups, and chemical linkers, all of which can be strategically modified to achieve favorable thermodynamic and kinetic properties for efficient electron transfer by these mediators.^163,164^ Redox polymers offer several advantages over diffusible molecular redox mediators, including consistent physical contact, improved mechanical stability, and, in some cases, protection from deactivation, e.g. by molecular oxygen. Moreover, like their small molecule counterparts, their redox potentials can be finely tuned through structural modification. By tuning the functional features of redox polymers, such as hydrophobicity, hydrophilicity, and charge, to match those of the biocatalyst or the light-harvesting unit, it is possible to promote specific interactions, including electrostatic interactions, or to physically entrap biological components within the polymer matrix.^165^ This may additionally allow achieving high catalytic loadings and enhance electron and mass transport.^166^ Despite these benefits, challenges remain in (1) the optimization of polymer conductivity, (2) maintaining mediator accessibility within densely packed or highly cross-linked matrices, and (3) reducing batch-to-batch variability in redox polymer synthesis. Based on whether the redox-active units in the redox polymer mediators are incorporated directly into the polymer backbone or attached via flexible tethers or linkers, redox polymers are typically classified as unbranched or branched (see discussion below).

***Redox moieties embedded in a conducting polymer backbone*** contain redox-active units immobilized within an electron-rich, π-conjugated polymer backbone. They facilitate efficient electron transport through a combination of intrachain delocalization and interchain hopping mechanisms. Riedel et al. reported that poly­(2-methoxyaniline-5-sulfonic acid)-co-aniline (PMSA1) can simultaneously act as a photosensitizer for the semiconducting mediator TiO_2_, and as a redox-active wiring polymer at the interface between TiO_2_ and the biocatalyst (PQQ-dependent glucose dehydrogenase).^38^ A dual-function redox polymer, polydihydroxy aniline (PDHA), acting as both a mediator and an immobilization matrix was reported by Weliwatte et al.^35^ It was shown, that intense light absorption by nearly black PDHA could compete with light harvesting in chloroplasts, and that one needs to design a layered architecture in order to achieve sufficient photocurrent density.

Redox moieties peripherally attached to a conducting/nonconducting polymer backbone via a spacer arm contain redox active units grafted to either a nonconductive or a conductive backbone *via* linkers. In the case of a nonconductive backbone, electron transfer is typically diffusion based hopping mechanisms.^167,168^ At the same time, for branched polymers with a conductive backbone, electron transfer involves multiple contributions from the backbone itself if it has involved a suitable redox potential, as well as from the redox-active units, and the linkers that connect them when closely attached.^169^ Zhao et al. showed that the hydrophobicity of branched redox polymers can be tuned to enhance biocatalyst integration by enhancing biocompatibility through three main strategies: (1) incorporating switchable functionalities (such as pH-responsive tertiary amines, thermo-responsive N-isopropylacrylamide segments, or photoswitchable azobenzene units) in the backbone, (2) introducing hydrophobic substituents at the redox-active moiety, and (3) embedding hydrophobic groups directly into the polymer backbone.^170^

### 4.2 Key Factors in the Selection of Redox Mediators

When selecting a redox mediator for a certain biocatalytic reaction, we consider several factors.

(1) The mediator’s redox potential must be aligned with both the excited state of the organic photosensitizer and the redox-active site of the biocatalyst to allow for thermodynamically favorable electron transfer. Large differences in energy or insufficient reducing potentials should be avoided.^76,155^ The electron transfer ability of organic photosensitizers toward redox mediators can be evaluated by analyzing their fluorescence quenching behavior using the Stern–Volmer relationship as a function of the redox mediator concentration (F_0_/F = 1+K_SV_ [M], where K_SV_ is the Stern–Volmer constant, F_0_ and F represent the fluorescence intensities in the absence and presence of the redox mediator, respectively).^91^ A higher K_SV_ value reflects more efficient quenching by the redox mediator and may serve as a valuable parameter for identifying the most compatible redox mediator for a specific organic photosensitizer. However, when applied to a nanoparticular photosensitizer, due to the microenvironment of the nanoparticle that traps the redox couple inside or the random (Poissonian) distribution of the redox mediator on the nanoparticle surface, the Stern–Volmer plot might not show a classic linear relationship typical of dynamic quenching. A static quenching model should be therefore considered.^31,171^

(2) The mediator should undergo fast and reversible redox cycling over multiple turnovers without degradation under operational conditions, effectively outcompeting both radiative and nonradiative recombination (*k*_*CR*_) of the excited organic photosensitizer (OPS*) to maximize catalytic efficiency. In this context, the electron transfer rate from the OPS to the redox mediator must exceed the excited-state lifetime of OPS* (*k*_*ET*_ > *k*_*CR*_), which can be quantified using time-resolved transient spectroscopy techniques.
(3) The ability to form long-lived and stable transient species beyond diffusion limits.
(4) The biocompatibility of the redox mediator should be assessed with the help of colony-forming unit assays,^165^ respiratory activity measurements, assessments of cell membrane integrity,^128^ or utilization of multiomic approach, etc (for details see Section 4.3).^166^
(5) The compatibility of the mediator and the organic photosensitizer/biocatalyst in terms of size, shape, and charge matching, as well as favorable interactions.
(6) Sufficient solubility and mobility in the reaction media and, where applicable, membrane permeability to access intracellular targets.
(7) Both photo- and chemical stability under operational conditions.

While the ultimate goal is to develop semiartificial photosynthetic systems that operate without the need for electron mediators and avoid an energy penalty, exploring environmentally and biocatalyst-friendly, as well as easily recyclable alternatives to conventional mediators remains essential. Both strategies, such as those eliminating mediators altogether and those replacing them with greener options, are critical for advancing more sustainable and efficient biohybrid systems.

### 4.3 Biocompatibility of Redox Mediators

The longevity of biological systems is inevitably dependent on the chemical environment, meaning that components that cause physiological stress to the biological system, can impact their long-term stability.^28^ Generally, biocompatibility can be assessed with the help of colony-forming unit assays,^172^ respiratory activity measurements, assessments of cell membrane integrity,^54^ or utilization of multiomic approaches, etc.^173^

Photophysical components, i.e., abiotic compounds that carry out light-driven electron transfer toward the biocatalyst (photosensitizer, sacrificial electron donor, and redox mediator) can, for example, influence protein folding and thus alter the activity of the respective enzyme if misfolding occurs. It is also important to consider that both redox mediators and sacrificial electron donors undergo redox reactions within the process, which results in an alteration in their reactivity. The latter can significantly influence biocompatibility as side-reactions can occur.^28^ Taking TEOA as an example, this sacrificial electron donor ensures efficient generation of photoreductant with photosensitizers like EY.^159^ However, Gamache et al could show that *E. coli*-based whole-cell systems that use a combination of EY as a photosensitizer, methyl viologen as a redox mediator, and TEOA as a sacrificial electron donor displayed very limited cell lifetimes,^28^ and plating assays showed that the cells did not survive longer than 24 h. The issue was determined to be mainly caused by the use of TEOA as a sacrificial electron donor and its degradation products (see Section 5.1.3 for details). In their studies, it was further shown that the substitution of TEOA with a biologically compatible sacrificial electron donor like L-cysteine significantly improved the biocompatibility without impacting the overall system performance.

Nonetheless, the sacrificial electron donor is not the only component that poses a potential toxicity to cells or enzymes. Redox mediators, upon one electron reduction, can also influence the biocompatibility of the respective biological components. Taking methyl viologen as an example, a MV^•+^-radical is formed upon single-electron reduction. One option is the formation of a C=C bond between the two pyridinium rings, accounting for the maintenance of a coplanar structure, as shown in Scheme 3.^155,159,174^ However, these radicals are highly reactive and can be involved in side-reactions besides electron transfer toward the biocatalyst. Therefore, cellular metabolism can be distorted and a stress reaction in whole-cell systems can occur. For enzymes, reactions with the amino acids in the protein are also conceivable, which can impact the secondary structures and, hence, the activity of the catalyst. As a result, MV is also thought to not be an optimal redox mediator in biohybrid systems.^28^

**Scheme 3:**
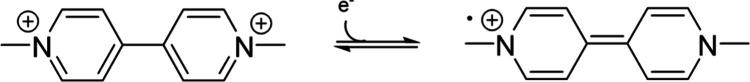
Structures of MV^2+^and MV^·+^ Oxidized MV^2+^ Features Positive Charges on the Nitrogens of the Pyridinium Rings^a^ ^,^ ^155,159,174^

a Upon one-electron reduction, a radical species MV^·+^ is formed, which is delocalized over the two pyridinium rings. Here, a representative localization at the pyridinium nitrogen with concomitant intramolecular electronic redistribution to form a C=C double bond between the two pyridinium rings is depicted on the right.

Diquat derivatives represent an alternative to MV, providing mediators with more reducing potentials, and have also shown improved biocompatibility in whole-cell systems.^28^

For photosensitizers, it is equally important to determine their biocompatibility, as not all photosensitizers are suitable for use in biological systems. In addition to that, it is also crucial to have the knowledge about whether the photosensitizers are intra- or extracellular when working with whole-cell systems, as this fact significantly influences whether a redox mediator is necessary or can be omitted. For example, EY is known to be intracellular and not cytotoxic, and it has been shown that whole-cell-based biohybrid systems are also functional without an additional redox mediator.^5,175^ In contrast, the metallic complex [Ru­(bpy)_3_]^2+^ cannot pass cellular membranes, and the electron transfer into the cytosol has to be enabled *via* redox mediators.^176,177^

## 5 Regeneration of Organic Photosensitizers

### 5.1 Challenges Introduced by Sacrificial Electron Donors

In organic-material-based biohybrid catalysis, different SEDs have been used, such as amines (TEOA, TEA, EDTA), organic acids (L-ascorbic acid, lactic acid), thiols (L-cysteine, DL-dithiothreitol), zwitterionic buffers (HEPES, MOPS), and others (see Table 4 for details). SEDs facilitate electron transfer efficiency and prolong catalyst lifetime, but their integration into biohybrid systems comes with significant challenges. Oxidation products of SEDs may inhibit enzymes or compete with target substrates, and the need for continuous replenishment limits the scalability and undermines process sustainability. Selecting an appropriate electron donor remains a challenging task, especially with regard to their suitability for an organic photosensitizer. For example, PNPs can often be affected by the high ionic strength of buffers, which can lead to aggregation and deactivation of light-harvesting NPs.^178^ Thus, the ability of SEDs to serve as “buffers” simultaneously is also highly appreciated, as it eliminates the necessity to use high concentrations of external salts and anions/cations from typical buffers.^179,180^

**Table 4:**
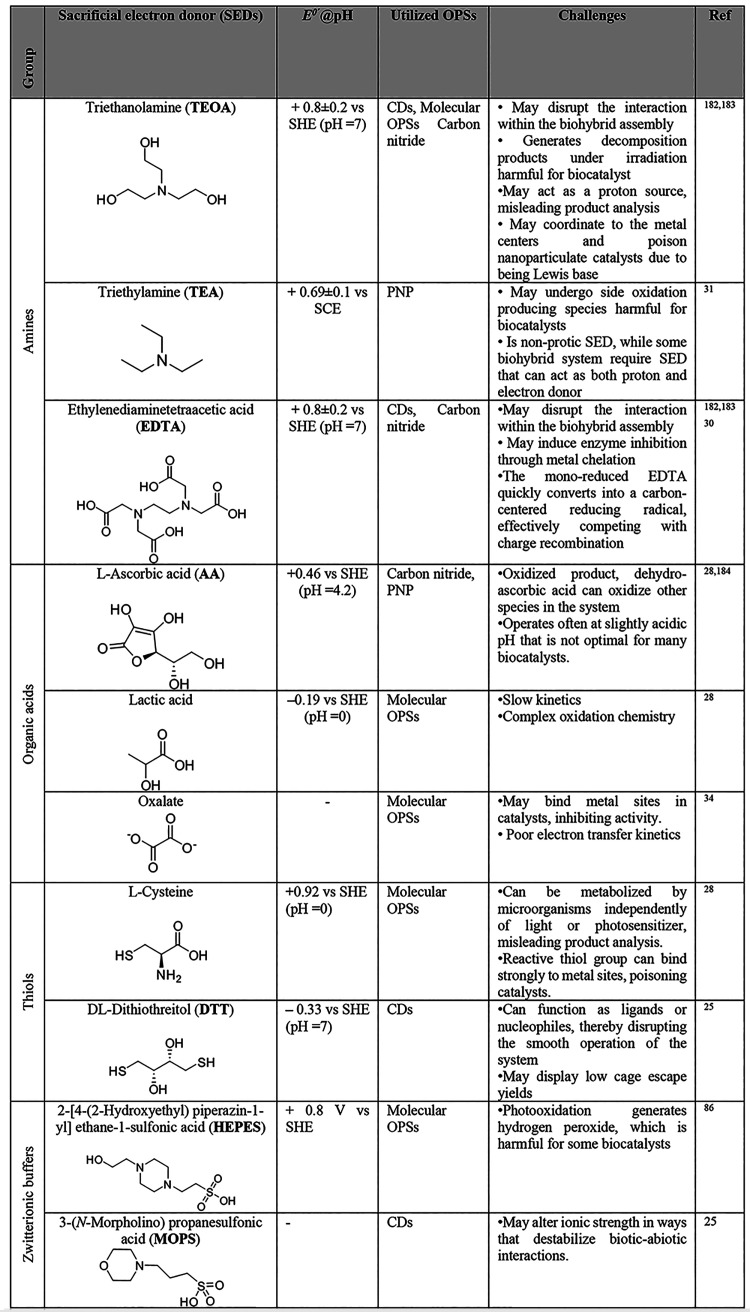

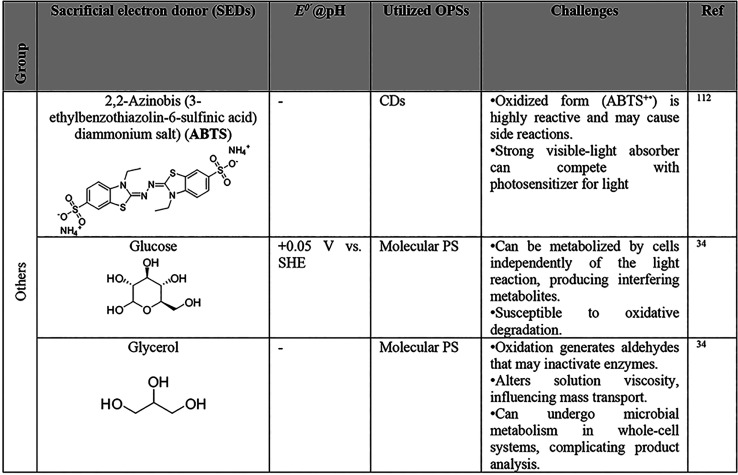
Structures and Key Challenges Introduced by the SEDs’ Properties in Biohybrid Assemblies Based on Organic Photosensitizers^a^ ^182,183,184^

a The reported redox potentials should be interpreted with caution and regarded as approximate indicators of the underlying thermodynamic driving forces, as literature values vary significantly and typically are highly pH dependent.

SEDs are generally regarded as reductants or oxidants that facilitate the regeneration of organic photosensitizers. Ideally, they should provide a sufficient thermodynamic driving force for the corresponding acceptor species, undergo rapid and irreversible oxidation into chemically inert products, and exhibit high cage escape yields.^181^ In practice, however, they can initiate several counterproductive processes that disrupt the interactions within the biohybrid assembly, reduce product yields, alter the pH, and, in some cases, lead to misleading conclusions about the origin of product formation. Therefore, in sections 5.1.1–5.1.3 and Table 4, we discuss the challenges introduced by the addition of sacrificial reagents.

#### 5.1.1 Interference of SEDs on Biohybrid System Interactions

The delicate balance of interactions within biohybrid assemblies can be disrupted by SEDs, which compete for binding sites on the photocatalyst or photosensitizer, thereby weakening biotic-abiotic coupling. Holá et al. investigated how different SEDs, and the electrostatic environment they create affect the interplay within a biohybrid assembly composed of CDs and [FeFe]-hydrogenase.^30^ They found that negatively charged CDs interacted more strongly with the positively charged active site of [FeFe]-hydrogenase in solutions containing TEOA as an SED, than in those containing EDTA. The authors hypothesized that this difference arose from the nature of the SEDs under photocatalytic conditions. At pH 7, negatively charged EDTA (predominantly in the HY_3_^–^ form) could bind to the enzyme’s positively charged active site, thereby blocking its interaction with the CDs. In contrast, positively charged TEOA allowed the biohybrid complex to maintain this interaction, supporting DET and resulting in a higher charge-transfer rate and improved photocatalytic performance.

Analysis of the impact of the electrostatic environment generated by SEDs was further expanded by Badiani et al. When using positively charged CDs (CD–NHMe_2_^+^) electrostatic repulsion with the positively charged TEOA (pH = 7) suppressed its electron donating efficiency and thus limited the overall performance of the biohybrid assembly. Replacement of TEOA with negatively charged EDTA showed the potential to promote electrostatic interaction and efficient electron transfer between the organic light-harvesting material and the SED. At the same time, this came at the cost of CDs surface charge shielding, and hindering from the enzyme binding in an electroactive orientation.^25^ It was summarized that the impact of the electrostatic environment generated by SEDs needs to be considered for both the organic photosensitizer and the biocatalyst when aiming to achieve efficient coupling within a biohybrid assembly. The authors suggested that SEDs that do not influence the surface charge of light-harvesting NPs and have a more favorable thermodynamic driving force, such as DL-dithiothreitol (DTT), could increase the fraction of enzyme moieties in a favorable orientation, leading to higher activities. Furthermore, Badiani et al. revealed that zwitterionic buffers such as MOPS are less likely to screen the electrostatic interaction between biohybrid assemblies and electrodes.^25^

#### 5.1.2 “Non-innocent” Metabolized Sacrificial Electron Donors

In some cases, SEDs that are commonly added to photocatalytic systems are not metabolically inert. Instead, they may be converted by the biocatalyst or other components of the hybrid system into reactive or competing intermediates that alter the reaction pathway and influence the yield of the desired product. Such side reactions can obscure the true origin of the final products, complicate mechanistic interpretations, and potentially result in inaccurate conclusions about the reaction process. A study that addressed the impact of “non-innocent” SEDs was reported by Göbbels et al.,^185^ who demonstrated that L-cysteine, a typical SED used in biohybrid catalysis, can be metabolized to acetate through intracellular pathways in *Moorella thermoacetica*, that are triggered without involving the photosensitizer (CdS) or light. By avoiding the use of “non-innocent” SEDs one can unmask the true efficiency of the photobiocatalytic processes and prevent metabolic cross-reactivity. In this regard, several research groups highlighted the necessity of performing additional experiments, such as isotope labeling, or high-resolution mass spectrometry, to accurately quantify the absolute concentrations of the associated products and to verify the catalytic reaction.^186,187^ Ideally, the use of sacrificial reagents and organic additives should be minimized or even eliminated.

#### 5.1.3 Toxic Byproducts from SEDs

The decomposition or side reactions of certain SEDs can result in the formation of toxic byproducts that are harmful to both biocatalysts and photosensitizers, thereby shortening the operational lifetime of biohybrid systems. In addition to impairing performance, toxic decomposition products pose environmental and safety concerns that may hinder large-scale applications. Careful selection of SEDs, along with strategies to neutralize or remove harmful byproducts, is therefore essential for maintaining the long-term activity and sustainability.

Among SEDs, TEOA and EDTA are notable for generating decomposition products during photocatalysis that are harmful to biohybrid assemblies.^181,188^ TEOA is a two-electron donor, implying that two oxidation steps are involved. Its single-electron oxidation yields an N-centered aminyl radical. This radical is a very potent oxidant, which implies the possibility of back electron transfer from the reduced form of the photosensitizer, redox mediator or catalyst, respectively.^189^ However, the radical is kinetically unstable, resulting in rapid radical delocalization on a time scale faster than that of electron back transfer reactions. Via radical delocalization, a C-centered radical is obtained, with two possible locations: the radical is located at either the α-position to the hydroxyl-group or the N-atom. The formation of a C-centered radical is also associated with deprotonation, whereby H^+^ is transferred to a second equivalent of TEOA. C-centered radicals are capable of the donating or accepting a second electron.^190,191^ With respect to the radical position, two pathways for further TEOA oxidation are conceivable. If centered on the C-atom in the α-position to the OH-group (pathway [1] in Figure 15), oxidation is associated with a second deprotonation step, yielding a terminal aldehyde. If the C-centered radical is in the α-position to the N-atom (pathway [2] in Figure 15, however, it is converted into a highly unstable imine radical. Subsequent decomposition upon addition of H_2_O and deprotonation leads to the formation of the secondary amine diethanolamine and hydroxyethanol, also referred to as glycolaldehyde. Both of these components are highly reactive and have been proven to exhibit toxicity toward microorganisms, such as *E. coli*, thus the longevity of suchlike biohybrid systems is impacted and it can be concluded that TEOA is not an optimal choice as a sacrificial electron donor when whole-cell-based systems or enzymes are used.^28^ Similarly, the oxidation of ascorbic acid yields dehydroascorbic acid, a reactive species capable of further oxidizing other components in the photocatalytic system. Therefore, transitioning toward more sustainable approaches, such as those excluding SEDs is desirable.^181^

**Figure 15:**
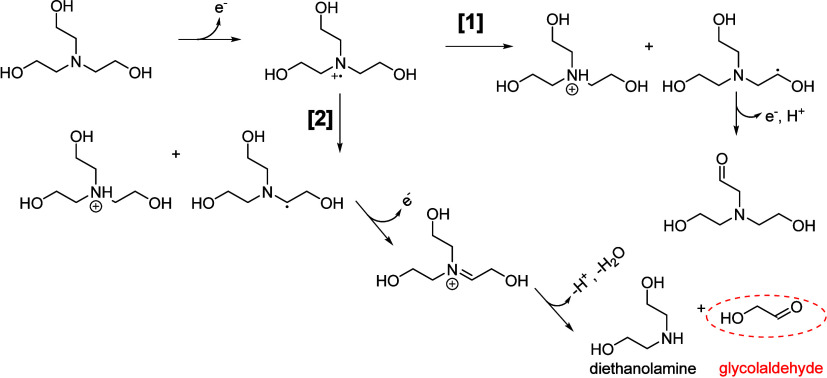
Decomposition pathways of TEOA upon two-electron oxidation. The initial one-electron oxidation yields an unstable N-centered radical species that is rapidly relocated, turning it into a C-centered radical. For the radical being located in the α-position to the OH-group, further oxidation and deprotonation yield a terminal aldehyde species (Pathway [1]). Instead, the radical position in α-position to the iminyl-group (Pathway [2]) is attributed to a decomposition pathway yielding diethanolamine and glycolaldehyde upon oxidation, deprotonation and H_2_O-addition. Reproduced from ref 181. Creative Commons CC BY 4.0.

### 5.2 Regeneration of Organic Photosensitizers by Water and Alternative Approaches

Water is often seen as the only sustainable and environmentally benign electron donor for the regeneration of photosensitizers that is available for large-scale Photobiocatalytic fuel production.^4,192^ In 2014, Park’s group presented a semiartificial system based on an inorganic photosensitizer that coupled biocatalytic transformations with photocatalytic water splitting.^193^ The system was designed to regenerate NAD­(P)H from NAD­(P)^+^ via a complete photocatalytic redox reaction rather than separate half-reactions. The idea was to enable the synthesis of complex chemicals, such as chiral alcohols and natural or unnatural amino acids, using redox enzymes fueled by in situ cofactor regeneration. By exploiting electrostatic interactions between light-harvesting inorganic dyes ([Ru­(bpy)_3_]^2+^) and redox catalysts, they achieved efficient charge separation. However, the overall efficiency, and thus the accumulation of the reduced cofactor, was limited by competing oxidation–reduction reactions, back electron transfer, and charge recombination. Almost at the same time, Mifsud et al. reported a biohybrid system that utilized a TiO_2_–based photocatalyst with flavin redox mediators in the presence of oxidoreductases to drive photobiocatalytic, water-driven bioredox reactions.^194^

While the use of water as an electron donor enables “clean” regeneration without byproduct accumulation, it also introduces several limitations when coupling the regeneration of organic photosensitizers by water to redox biocatalytic reactions. These challenges primarily arise from (1) slow water oxidation reaction kinetics due to its multicharge nature and (2) the risk of oxygen evolution causing the deactivation of biocatalysts, (e.g., oxygen-sensitive enzymes), as well as potentially generating reactive oxygen species.

A promising approach to address the challenges introduced by water as an electron donor is the use of PEC devices, which offer spatial separation of oxidation and reduction reactions, thereby maximizing product separation, suppressing unwanted side reactions, and enabling optimal conditions for both redox reactions (see Section 6 for details). At the same time, it is important to note that PEC assemblies are often technically complex, and in some cases they require elaborate electrodes and ion-exchange membranes, additionally posing challenges in maintaining the neutral pH required by biohybrids due to mass-transport-induced pH gradients.^12^

An alternative approach is to incorporate additional catalysts into the biohybrid assembly or to use other substrates besides water that can serve as feasible oxidation targets in scaled-up systems, thus closing the redox cycle. Advancing the field will require a dual approach: overcoming the challenges associated with currently used SED strategies while simultaneously developing alternative pathways that enable oxidation reactions yielding more value-added products. Building on the need to move beyond conventional SED-dependent systems, alternative oxidation strategies have emerged that sustain photobiocatalytic activity while simultaneously generating value-added products at the oxidative side of the photocatalytic cycle. These approaches aim to couple the reductive half-reaction to the selective oxidation of organic substrates, enabling paired catalysis in which both half-reactions yield useful chemicals. To date, there are not many examples of such systems. When metabolite-associated biocatalysts are integrated into biohybrid systems, endogenous metabolites generated through native metabolic pathways may be strategically utilized as electron donors for photosensitizer regeneration, rather than being treated merely as metabolic byproducts.^60^ In this context, the system leverages pre-existing metabolic flux and redox-balancing processes, thereby minimizing reliance on externally added sacrificial electron donors and avoiding unnecessary energy dissipation into nonproductive pathways. For example, amino acids naturally secreted by certain bacteria, which were raised from overflow metabolism or redox homeostasis, can serve as in situ electron donors to regenerate polymeric photosensitizers and sustain continuous catalysis. This highlights the importance of matching the optical energy levels of the light-harvesting material (e.g., polymer PFP in this case) with the redox potentials of intracellular proteins, highlighting the need to optimize the material-microbe interface for efficient electron transfer.^60^

#### 5.2.1 Proposed Limitations and Failure Modes

Biohybrid photocatalytic systems are prone to several recurring failure modes that can compromise the mechanistic interpretation and reproducibility. First, redox mediators such as methyl viologen (MV^2+^) may generate highly reactive radical intermediates (MV^•+^), leading to cytotoxicity, enzyme modification, and competing side reactions; therefore, mediator stability, reactivity, and biocompatibility should be systematically evaluated, and alternative mediators (e.g., diquat derivatives with lower radical reactivity) considered. Second, SEDs are often not metabolically inert; their degradation products or metabolic conversion can distort cellular redox balance, induce stress responses, and alter reaction selectivity. Control experiments assessing the cell viability, enzyme integrity, and product distribution in the absence of illumination are therefore essential. Third, mediator and SED photophysics (competitive light absorption, surface charge modulation, and nanoparticle aggregation under high ionic strength) can significantly affect interfacial charge transfer and must be carefully controlled through spectral characterization, buffer optimization, and surface-interaction studies. Fourth, reactive oxygen species (ROS) formation and oxygen sensitivity of enzymes require monitoring of O_2_ levels and appropriate headspace control. Finally, where water is used as an electron donor, slow oxidation kinetics and oxygen evolution may limit the performance or deactivate biocatalysts; spatial separation strategies such as PEC architectures or coupling to alternative oxidation catalysts can mitigate these effects. Collectively, systematic reporting of mediator/SED stability, toxicity, redox potentials, control experiments, and long-term activity retention is critical to avoid misinterpretation and improve reproducibility.

## 6 Design Principles for Organic Light-Harvester-Based Biohybrid Photoelectrochemical Systems

Photoelectrochemical (PEC) systems are integrated platforms in which all functional components, including light-harvesting units, charge separation layers, and catalytic sites, are coimmobilized onto a charge-collecting electrode. They harness solar energy by directly coupling photoinduced charge generation with electrochemical transformations at the electrode, enabling the conversion and storage of solar energy in chemical bonds at the electrode/electrolyte interface. This architecture simplifies downstream catalytic applications, enhances device compactness, and offers potential reductions in operational costs. The high degree of integration on the electrodes also facilitates catalyst recovery and system recyclability, which are critical for long-term operation. Consequently, PEC devices have been successfully utilized in a wide range of catalytic reactions, including water splitting, CO_2_ reduction, and value-added organic synthesis, *etc.* However, conventional fully abiotic PEC devices often face substantial limitations, including high overpotential requirements, limited product selectivity, and dependence on critical-metal catalysts. These challenges constrain their scalable applications in solar-to-chemical conversion. To overcome these limitations, the integration of biocatalysts with abiotic photoelectrode substrates has led to the development of biohybrid PEC systems. The resulting hybrid devices combine the unique catalytic properties of biological components such as low overpotential requirements, high substrate specificity, critical-metal-free active sites, and operation under mild conditions. As a result, biohybrid PEC devices represent a promising next-generation platform for high-efficiency, selective, and sustainable solar-to-chemical energy conversion.

This section focuses exclusively on biohybrid photoelectrochemical (PEC) systems in which the primary light-harvesting unit is organic, in the form of (i) biogenic pigment–protein complexes (e.g., PSI/PSII), (ii) molecular organic dyes, or (iii) conjugated organic semiconductors (OPV materials) (Figure 16). Additionally, biohybrid photocathodes and photoanodes have also been integrated into biohybrid solar cell architectures for electricity generation. As this topic has been comprehensively covered in previous reviews,^195,196^ it is not discussed in detail in the present article. We deliberately exclude PEC systems in which inorganic semiconductors serve as the dominant light absorbers as those fall outside the scope of this review. Instead, the objective here is to analyze how organic light harvesters dictate device energetics, charge separation behavior, photocurrent generation, and catalytic performance when they are interfaced with biocatalysts.

**Figure 16:**
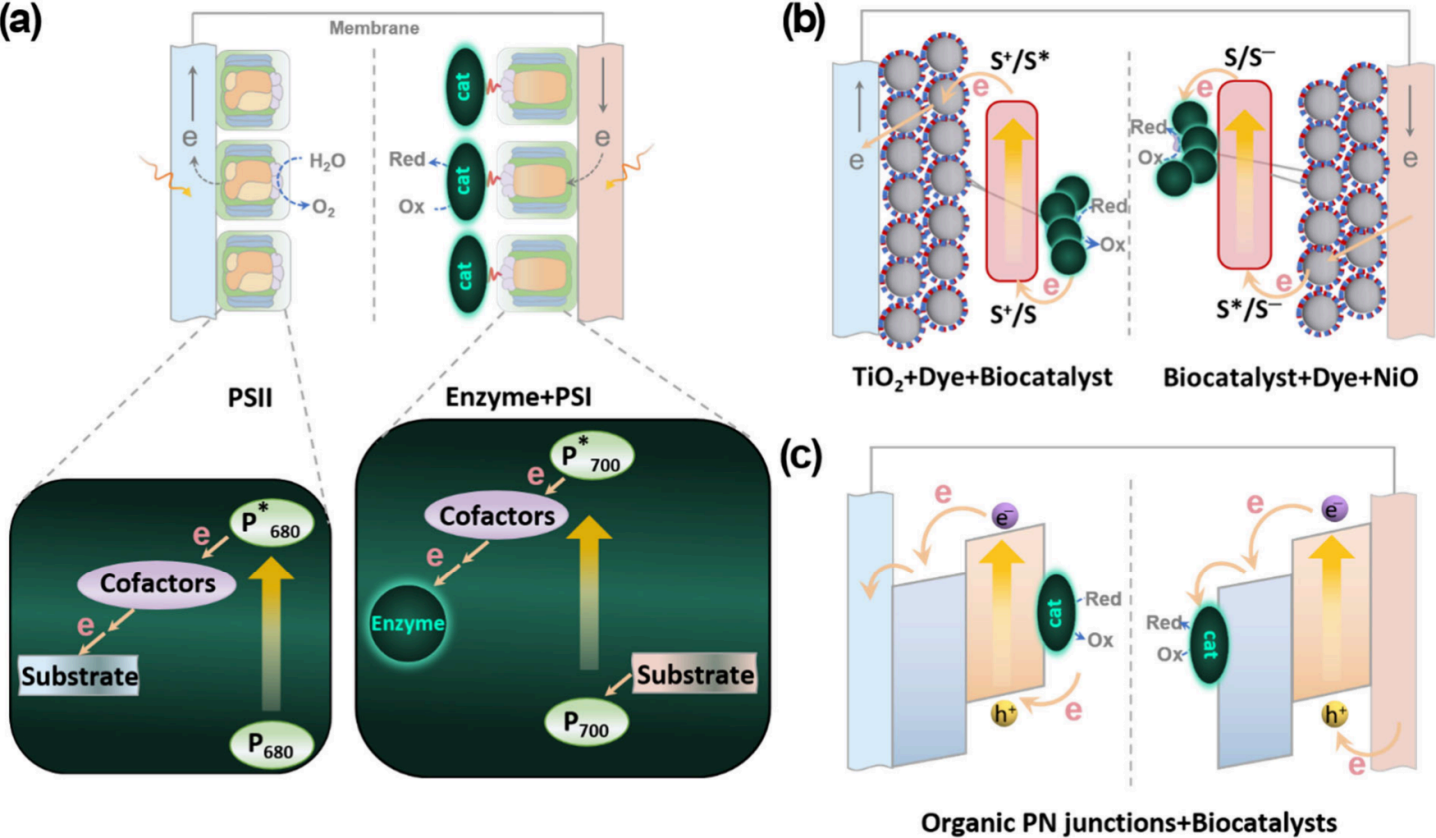
Three categories of organic-based biohybrid PEC devices: (a) Type I, conductive electrode + Enzymes linked PS light-harvesting center; (b) Type II, Dye-sensitized metal oxide semiconductor + biotic catalyst; (c) Type III, Organic photovoltaic semiconductor + biocatalyst.

Organic-light-harvester-driven PEC architecture can be categorized into three mechanistically different types, defined by how light absorption and charge separation are achieved. (Type I) Biogenic organic photosensitizer systems (PSI/PSII-based).

In these devices, light harvesting and primary charge separation occur within the protein-embedded chlorophyll network of PSI or PSII. The abiotic electrode functions purely as a conductive collector. Device efficiency is therefore governed by (i) protein orientation, (ii) electronic coupling to the electrode, and (iii) the suppression of interfacial recombination. Because PSI exhibits near-unity internal quantum efficiency, performance limitations arise predominantly from external factors such as spectral gaps (500–650 nm), limited absorption cross-sections, and wiring efficiency. (Type II) Molecular organic dye-sensitized systems. Here, a synthetic organic dye serves as the exclusive visible-light absorber. The inorganic scaffold (e.g., TiO_2_, NiO) functions only as a charge-separation and transport layer. Device performance is dictated by dye energetics (HOMO/LUMO alignment), excited-state lifetime, dye regeneration kinetics, and interfacial recombination pathways. Unlike Type I systems, spectral coverage and redox potentials can be more easily systematically tuned through molecular design. (Type III) Organic semiconductor (OPV)-based systems. In this configuration, conjugated polymers or small-molecule bulk heterojunctions perform both light harvesting and charge separation. These systems benefit from high absorption coefficients, tunable energy levels, and scalable fabrication. However, exciton binding energy and interfacial enzyme coupling become the dominant limiting factors. Among organic-based biohybridPEC systems, the Type III architecture typically delivers the highest photocurrent densities.

Across all three types, the central design challenge is the alignment of organic absorber energetics with the redox potential of the biocatalyst. The conduction band (or LUMO) must provide sufficient thermodynamic driving force for reduction reactions while maintaining a bandgap narrow enough to efficiently harvest visible light. Organic materials offer a particular advantage in this respect: their frontier orbital energies and absorption spectra can be rationally engineered via chemical modification, donor–acceptor design, or heterojunction formation.

The following subsections analyze photocathodes and photoanodes exclusively through this organic light-harvester lens, highlighting quantitative performance metrics and extracting design principles specific to organic materials integration.

### 6.1 Organic Material-Based Biohybrid Photocathodes

Biohybrid photocathodes are engineered devices that harness incident photons to generate electron–hole pairs, with photoexcited electrons directed toward immobilized biocatalysts to drive reductive transformations at the biocatalyst–electrolyte interface. Simultaneously, the photogenerated holes are extracted through the underlying substrate, either under an applied external bias or *via* an internal built-in potential. This architecture enables the coupling of efficient light harvesting with high selectivity and mild operational conditions of biocatalytic processes and allows the separation of the cathode reaction and anode reaction with different chambers. Emerging applications of biohybrid photocathodes include the hydrogen evolution reaction (HER),^51,197^ carbon dioxide reduction reaction (CO_2_RR),^55,56^ oxygen reduction reaction (ORR),^66,198^ and ketone hydrogenation reaction (KHR),^48^ *etc.*

#### 6.1.1 Hydrogen Evolution Reaction

Zhao et al. exploited the amphiphilic nature of PSI to fabricate a monolayer at the water/air interface, which could then be subsequently transferred onto a gold-coated electrode surface with controlled orientation, ingeniously positioning the stromal side outward and the luminal side in contact with the electrode by the amphipathic nature of PSI.^197^ To facilitate efficient electron transfer while minimizing CR and short-circuiting, the PSI layer was laminated between two redox polymers: a P-type mediator Os-complex polymer beneath and an N-type mediator viologen-based polymer above. For catalytic hydrogen production, they further immobilized a H_2_ase atop the viologen polymer matrix (Figure 17b). Photocurrent generation from the assembled photocathode was confirmed under chopped-light linear sweep voltammetry (LSV) within the H_2_ase assembled device with a photocurrent of around 1.6 μA cm^–2^ (Figure 17c). Moreover, they verified hydrogen evolution *via* mass spectrometric detection of the *m*/*z* = 2 peak. The photocathode was then coupled to a PSII-based photoanode to construct a tandem biohybrid Z-scheme system, successfully achieving light-driven water splitting under bias-free conditions. Nevertheless, the photocurrent achieved in this work is still far below the industrial standard, which requires more efficient photo-to-electron conversion systems.

**Figure 17:**
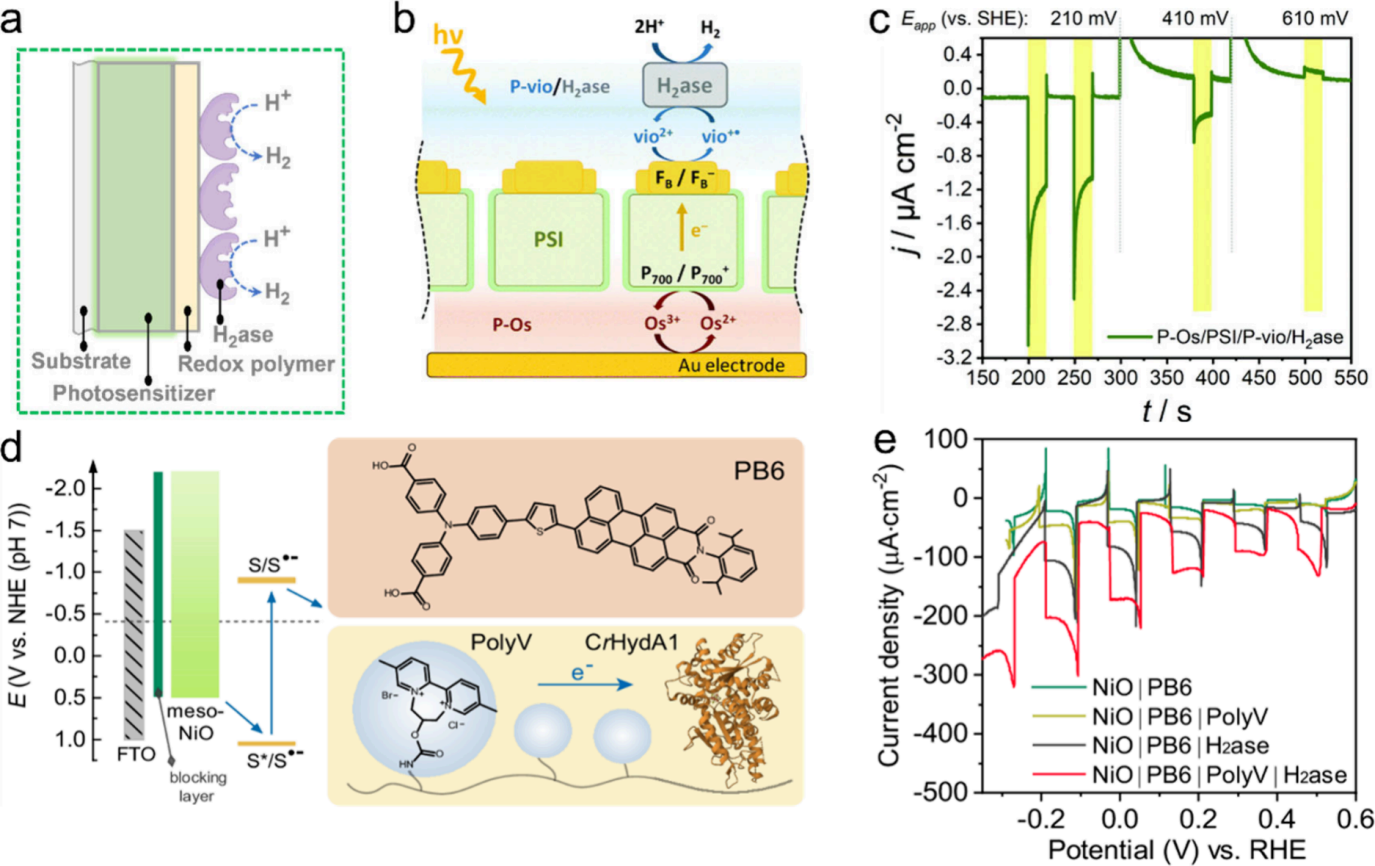
(a) Scheme of type I biohybrid PEC for H_2_ production; (b) Device diagram of a H_2_ase-immobilized PSI based biohybrid photocathode, and (c) its photocurrent response under chopping light LSV; (d) Device diagram of a H_2_ase immobilized dye-sensitized NiO based photocathode for H_2_ production, and e) its photocurrent response under varied conditions. Panel **b**, **c** reproduced with permission from ref 197. Copyright (2019) Royal Society of Chemistry; Panel **d**, **e** reproduced from ref 51. Available under a CC-BY 4.0 license. Copyright (2024) Springer Nature Ltd.

In a related study, we developed a dye-sensitized NiO-based photocathode for hydrogen evolution by immobilizing a hydrogenase through a viologen-functionalized redox polymer.^51^ Upon photoexcitation, the sensitizing dye injects photogenerated holes into the NiO valence band, while electrons are transferred through the viologen polymer to the hydrogenase, where catalytic hydrogen production occurs (Figure 17d). The photocurrent response, assessed via chopped-light LSV, revealed a stepwise increase upon sequential incorporation of the viologen redox polymer and H_2_ase, with the highest photocurrent observed when both components were present, highlighting the successful construction of an efficient photoinduced electron transfer pathway from the dye to the H_2_ase through the redox polymer (Figure 17e). Consequently, the fully assembled biohybrid NiO photocathode exhibited a photocurrent of around 141 μA cm^–2^ at neutral pH under 0 V vs the reversible hydrogen electrode (RHE), along with a stable output over 5 h of continuous operation and a faradaic efficiency (FE) of 94%, which represents one of the best catalytic performances ever reported with similar architecture. To demonstrate the feasibility of integrated solar water splitting, the H_2_ase-immobilized photocathode was paired with a BiVO_4_ photoanode to construct a bias-free tandem PEC device. With this system, it achieved a solar-to-hydrogen (STH) conversion efficiency of 0.124%, maintaining stable performance with only minor degradation over 10 h of continuous illumination.

#### 6.1.2 Carbon Dioxide Reduction

The excited-state light-harvesting center P700* in PSI exhibits a highly negative reduction potential of approximately −1.2 V vs RHE, enabling it to directly transfer electrons to a wide range of redox enzymes to drive desired catalytic transformations. Utilizing this strategy, Morlock et al. designed a macroporous inverse-opal ITO substrate, which offers a large surface area for PSI immobilization, high electrical conductivity for efficient charge collection, good light transmittance, and a biocompatible environment that supports long-term enzyme functionality.^55^ In their system, FDH derived from *Methylobacterium extorquens* was immobilized atop the PSI layer, establishing an efficient direct electron transfer pathway from the photoexcited PSI to FDH (Figure 18a). Upon illumination, the device generated a photocurrent of approximately 7.12 μA under chopped-light chronoamperometry, which remained stable for nearly 2 h before gradually declining (Figure 18b). After 22 h of continuous operation, approximately 10% of the initial photocurrent was retained. Despite relatively low photocurrent achieved, the high catalytic activity of the enzyme still produced 0.2 μmol of formic acid with a FE of ∼15%.

**Figure 18:**
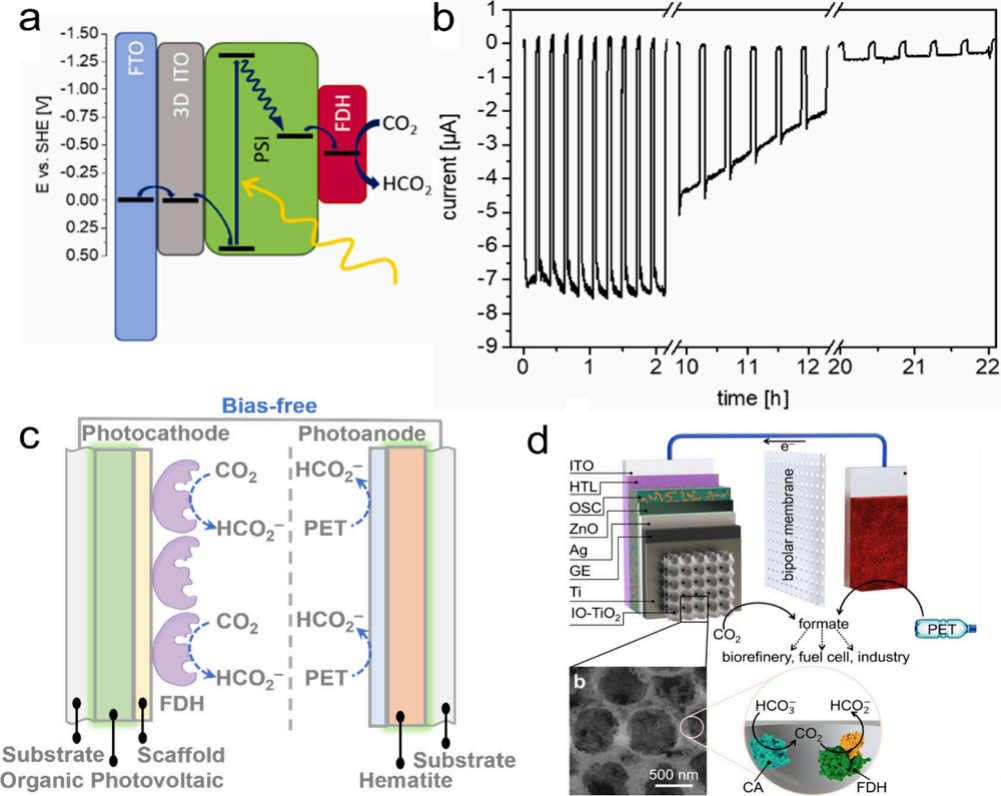
(a) Scheme of biohybrid PEC device architecture of a FDH immobilized PSI based photocathode for CO_2_ reduction, and (b) its photocurrent response under chopping light LSV; (c) Scheme of combining a biohybrid photocathode with a photoanode to achieve bias-free tandem device; (d) FDH immobilized OPV based photocathode for CO_2_ reduction, coupled a hematite photoanode. Panel **a**, **b** reproduced from ref 55. Available under a CC-BY-NC-ND license. Copyright (2024) Elsevier; Panel **d** reproduced from ref 56. Available under a CC-BY 3.0 license. Copyright (2025) Elsevier.

To enhance CO_2_ reduction performance, Liu et al. developed an OPV-based photocathode that offers several advantages, including earth-abundant composition, solution processability, tunable energy levels, and favorable optoelectronic properties for strong light absorption and efficient charge separation.^56^ This OPV photocathode was interfaced with FDH and carbonic anhydrase (CA) via a macroporous inverse-opal TiO_2_ scaffold, enabling effective enzyme immobilization and electron transfer. The coimmobilization of FDH and CA allowed the system to utilize dissolved carbonate ions as an in situ CO_2_ source, thus enabling efficient CO_2_ reduction under mild conditions without the need for noninnocent buffers or chemical additives associated with high concentration CO_2_. This coenzyme strategy could also be applied in similar pH-sensitive situations. Under operational conditions, the biohybrid photocathode achieved a photocurrent of up to 10 mA cm^–2^ at 0 V vs RHE. Furthermore, chopped-light chronoamperometry conducted over 10 h at 0.6 V vs RHE showed an initial photocurrent of approximately 3 mA cm^–2^, with a FE exceeding 90%, representing one of few mA scale photocurrent reports in biohybrid PEC systems. In contrast, control experiments without the enzymes yielded a negligible photocurrent, indicating the essential role of the biocatalysts to promote substrate conversion. When integrated with a hematite photoanode into a bias-free tandem configuration, the system enabled simultaneous formic acid production at both the cathode and the anode, with CO_2_ reduction on cathode side and plastic oxidation on anode side. This dual-site reaction achieved a combined formic acid FE approaching 200% over 10 h of continuous illumination, with the reduction side and oxidation side producing the same product. Thus, it demonstrated the remarkable efficiency and potential of the OPV-based biohybrid PEC architecture for solar-driven CO_2_ conversion.

#### 6.1.3 Oxygen Reduction Reaction

Biocompatible interfacial microenvironment between the abiotic electrode and the biotic catalyst and the biocatalyst loading enhancement are critical to construct biohybrid PEC devices. To this end, Morlock et al. developed a three-dimensional (3D) reduced graphene oxide (rGO) electrode using a templated fabrication approach, which further wired with PSI for the O_2_ reduction reaction in the absence of a native electron acceptor, O_2_^–^ radicals are formed by PSI (Figure 19a).^198^ Device optimization revealed that a short incubation time with high concentrations of the PSI bath yielded a higher photocurrent, likely due to improved surface coverage and preserved protein functionality. To evaluate the influence of the electron transfer mechanisms, device performance was compared under DET and MET conditions by assessing photocurrents in the absence and presence of cytochrome c (cyt c), respectively (Figure 19b). The introduction of cyt c as a redox mediator enhanced photocurrent generation by over an order of magnitude compared with DET-based configurations (Figure 19c). Moreover, a significantly faster photocurrent response was observed after adding cyt c, with an improved electron transfer kinetics. The results indicated that DET process was less efficient than the MET mechanism in the PSI-rGO interface. However, due to the biocompatibility of the r-CO nature, the as-prepared biohybrid PEC device exhibited promising operational stability, maintaining activity over several days in solution. Moreover, the researchers found a relatively high external quantum efficiency (EQE) of 6.8% under low light intensity (0.1 mW cm^–2^), highlighting the potential of engineered 3D interfaces for high-performance biohybrid solar energy conversion.

**Figure 19:**
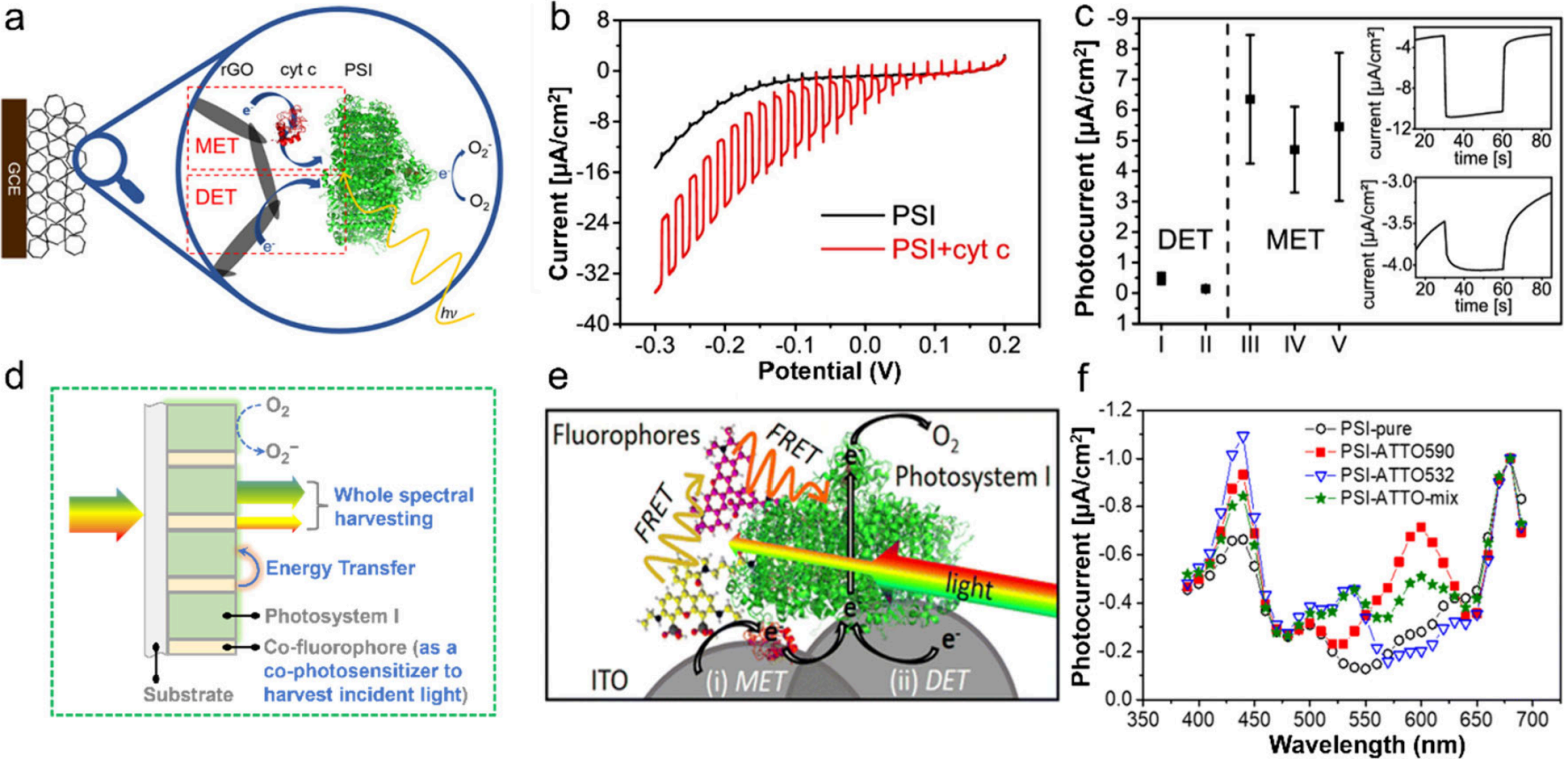
(a) Scheme of biohybrid PEC device architecture of a PSI based photocathode for O_2_ reduction, (b) its photocurrent response under chopping light LSV; and (c) the comparison between DET and MET; (d) Scheme of cosensitizer strategy to improve light harvesting ability of PS I-based device; (e) Device architecture of artificial dyes and PSI coimmobilized photocathode for O_2_ reduction, (f) its photocurrent response under different incident wavelengths; Panel **a**, **b**, **c** reproduced from ref 198. Copyright (2021) American Chemical Society; Panel **e**, **f** reproduced from ref 66. Available under a CC-BY 3.0 license. Copyright (2023) Royal Society of Chemistry.

Although PSI exhibits near-unity internal quantum efficiency, its external quantum efficiency remains significantly limited due to its reliance on chlorophyll-based light-harvesting pigments. Specifically, PSI possesses an inherent spectral absorption gap between 500 and 650 nm, where solar photon capture is minimal. To address this limitation, the same research group introduced two artificial dyes with absorption maxima centered within the PSI gap region and coimmobilized them with PSI onto a 3D ITO substrate (Figure 19e).^66^ Fluorescence spectroscopy confirmed efficient FRET from the excited artificial dyes to PSI, resulting in enhanced photocurrent responses specifically within the spectral window originally underutilized by PSI. This modification effectively broadened the device’s spectral-response and improved solar energy utilization across the full visible spectrum (Figure 19f). Furthermore, they found that the photocurrent difference between DET and MET configurations was significantly reduced in the dye-modified PSI systems, compared to those with unmodified PSI. This observation suggests that the coimmobilized dyes may contribute to improved PSI orientation on an electrode surface, thereby facilitating not only more consistent electron transfer irrespective of the transfer mechanism but also reducing interfacial charge recombination.

#### 6.1.4 Ketone Hydrogenation Reaction

Biocatalysts offer a unique advantage in enabling stereoselective product synthesis, which remains a highly challenging task for conventional abiotic catalysts. To achieve efficient solar-driven chiral alcohol production, Bouwens et al. developed a biohybrid PEC system incorporating an OPV photocathode as the light-harvesting and charge-separation unit, coupled with a cascade of three immobilized enzymes.^48^ The enzymatic sequence begins with FDH_*NVH*_ from *Nitratidesulfovibrio vulgaris*, which catalyzes the reduction of CO_2_ to formate (HCOO^–^). The second enzyme, FDH_*CB*_ from *Candida boidinii*, is an NAD^+^-dependent formate dehydrogenase that facilitates the regeneration of NADH from formate oxidation. Finally, the third enzyme is an ADH, which uses the *in situ* generated NADH to reduce acetophenone to enantiomerically pure 1-phenylethanol (Figure 20a). The stereoselectivity of the final product, either (*S*)- or (*R*)-1-phenylethanol, is governed by the specific ADH isoform (ADH_*S*_ or ADH_*R*_) employed.

**Figure 20:**
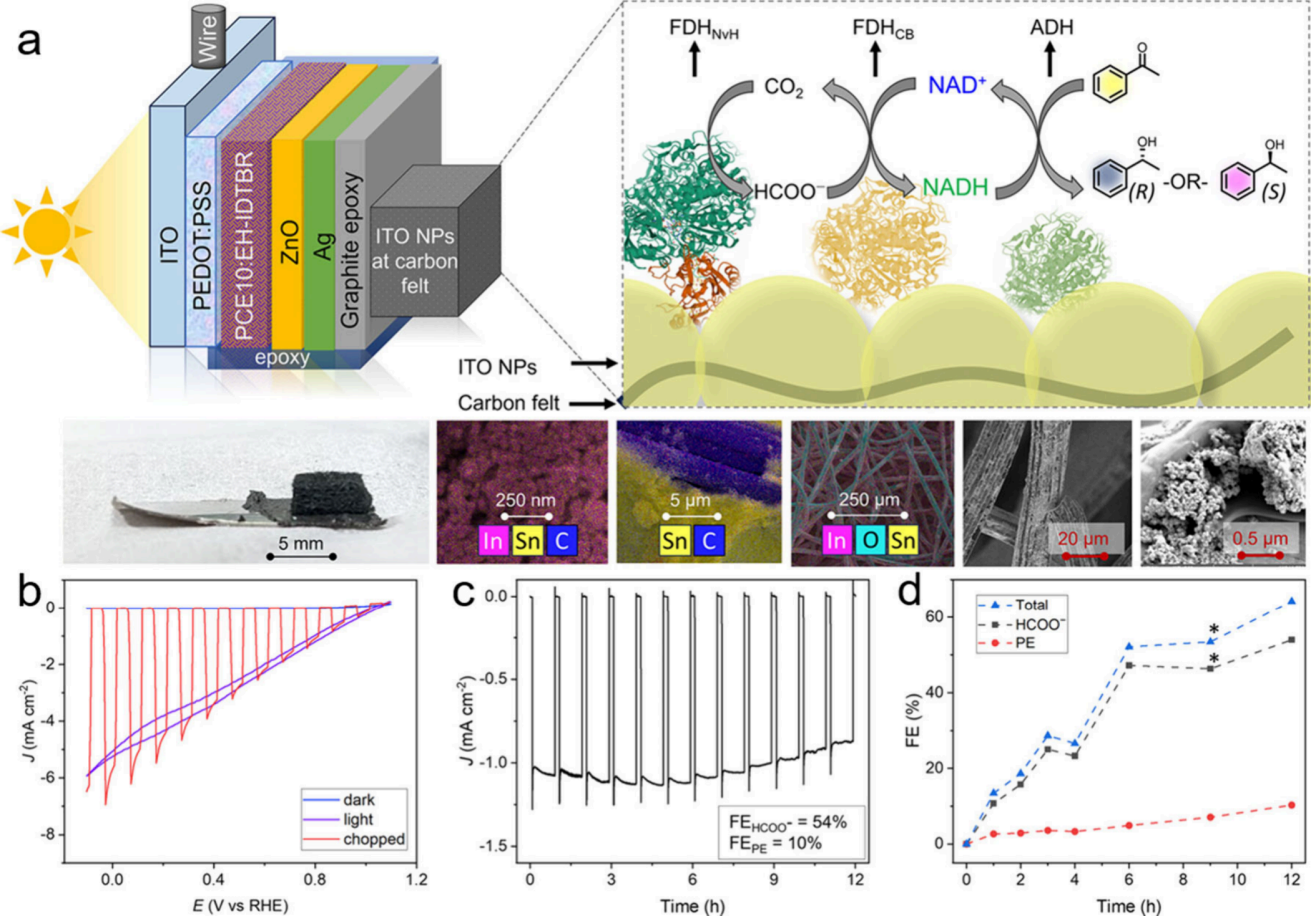
(a) Scheme of OPV based photocathode immobilized with a cascade of enzymes for ketone reduction, (b) its photocurrent response under chopping light LSV; and (c) chronoamperometry test under bias of 0.8 V vs RHE; (d) Product Faradaic efficiency over different time scale. Panel **a**, **b**, **c**, **d** reproduced from ref 48. Copyright (2025) American Chemical Society.

The OPV photocathode and the enzyme cascade could be independently optimized and electrically connected via a graphite–epoxy paste interface, enabling flexible tuning of the materials and biocatalytic functions. Under simulated solar illumination (100 mW cm^–2^), the biohybrid PEC device exhibited an onset potential of 1 V vs RHE and delivered a high photocurrent of approximately 6 mA cm^–2^ at 0 V vs RHE (Figure 20b). Notably, the system maintained a relatively stable photocurrent of ∼1 mA cm^–2^ at 0.8 V vs RHE over 12 h of operation (Figure 20c). Product analysis revealed an FE of 10% for chiral 1-phenylethanol and 54% for HCOO^–^, demonstrating effective electron utilization and CO_2_-mediated NADH regeneration for chiral synthesis (Figure 20d). This work represents a significant advance in solar-to-chemical energy conversion by integrating multienzyme catalysis with organic semiconductor platforms, highlighting the feasibility of sustainable production of stereochemically defined organics driven by solar energy and CO_2_. Therefore, bulk heterojunction organic semiconductors provide superior photon absorption and charge separation. The dominant limitation shifts to enzyme–electrode electronic communication and long-term operational stability.

### 6.2 Organic Material-Based Biohybrid Photoanodes

In contrast to photocathodes, biohybrid photoanodes feature a similar device architecture fabricated on an electrode but operate with reversed charge carrier dynamics. Upon light absorption, photoexcited holes are directed toward immobilized biocatalysts at the photoanode–electrolyte interface, where they drive oxidative transformations. Concurrently, photogenerated electrons are extracted through the conductive substrate. Emerging applications of biohybrid photoanodes include the oxygen evolution reaction (OER)^35,36^ and the glucose oxidation reaction (GOR),^37,38^ *etc.*

#### 6.2.1 Oxygen Evolution Reaction

Water oxidation enables the use of water as a sustainable electron and proton source for solar fuel and solar chemical production, such as CO_2_ fixation and H_2_ evolution. For instance, Sokol et al. developed a cosensitized TiO_2_ photoanode by integrating PSII with a spectrally and redox potentially complementary artificial dye, DPP, to drive the oxygen evolution reaction. Upon illumination, the DPP dye injects electrons into the TiO_2_, while photogenerated holes in PSII drive water oxidation (Figure 21a).^49^ A Z-scheme electron transfer was then constructed, facilitated by an Os-based redox polymer, which shuttles electrons from reduced PSII to oxidized DPP, thus regenerating both components. Moreover, this Z-scheme can also improve the solar spectrum utilization by the complementary light harvesting region of PSII and artificial dye. Meanwhile, electrons collected at the substrate were transferred to and used for CO_2_ reduction by FDH at the counter electrode. The incorporation of PSII into the DPP-sensitized TiO_2_ photoanode led to more than a 2-fold increase in photocurrent, as demonstrated by chopped-light step potential chronoamperometry, attributed to enhanced charge separation stemming from PSII-driven water oxidation (Figure 21b). PSII/dye cosensitized TiO_2_ photoanodes illustrate how complementary organic absorption windows improve spectral utilization. Meanwhile, redox polymers play a decisive role in suppressing recombination and facilitating the Z-scheme charge transfer.

**Figure 21:**
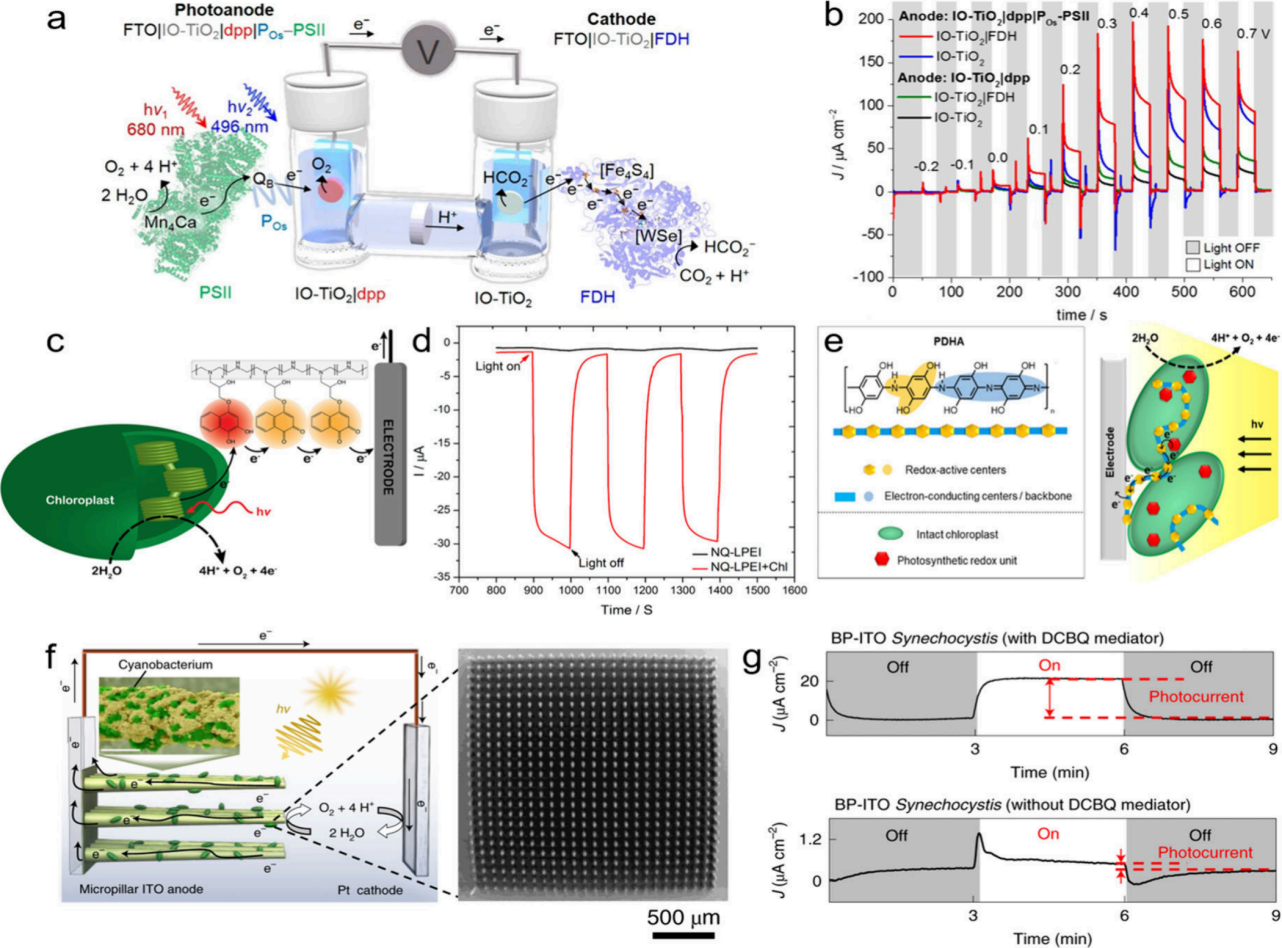
(a) Scheme of artificial dye and PSII cosensitized TiO_2_ photoanode, coupled with a FDH immobilized TiO_2_ as cathode, and (b) its photocurrent response under chopping light LSV define oxidative photocurrent as negative; (c) chloroplast immobilized biohybrid PEC photoanode wired with branched redox polymer; and (d) its photocurrent response under chopping light LSV with/w.o. biocatalyst, define oxidative photocurrent as negative; (e) chloroplast immobilized biohybrid PEC photoanode wired with linear redox polymer; (f) cyanobacterium immobilized micropillar ITO photoanode, inserted magnified SEM image of micropillar ITO electrode, and (g) its photocurrent response with/w.o. DCBQ. Panel **a**, **b** from ref 49. Copyright (2018) American Chemical Society; Panel **c**, **d** from ref 199. Copyright (2017) American Chemical Society; Panel **e** from refs 35, 49, 199 . Copyright (2021) American Chemical Society; Panel **f**, **g** reproduced with permission from ref 36. Copyright (2022) Springer Nature Ltd.

Despite PSII’s high catalytic efficiency for the OER, its complex purification and susceptibility to photoinactivation pose major limitations for large-scale application. Chloroplasts, photosynthetic organelles in oxygenic organisms, offer a more practical alternative due to their easier extraction and enhanced stability, supported by their protective membrane structures and intrinsic self-repair mechanisms. To overcome electron-transfer barriers from insulating membranes and facilitate interfacial charge transfer inherent to these biocatalysts, Hasan et al. employed a naphthoquinone-grafted linear polyethyleneimine (NQ-LPEI) polymer as an electron mediator (Figure 21c).^199^ This redox polymer not only promoted electron transport but also served as a 3D immobilization matrix for the biocatalyst. The resulting biohybrid photoanode showed more than a 3-fold enhancement in photocurrent compared to its DET configuration counterpart (Figure 21d). Similarly, Weliwatte et al. reported the use of PDHA, a linear redox polymer, to wire chloroplasts to the electrode, achieving over a 4-fold increase in photocurrent relative to devices lacking the redox polymer (Figure 21e).^35^ Extending beyond subcellular components, Chen et al. developed a whole-cell photoanode by immobilizing cyanobacteria onto micropillar-patterned ITO electrodes for water oxidation (Figure 21f).^36^ To overcome the multiple membrane barriers within the cyanobacterial cells, a membrane-permeable small molecule redox mediator, 2,6-dichloro-1,4-benzoquinone (DCBQ), was introduced, resulting in a nearly 2 orders of magnitude increase in photocurrent (Figure 21g). When membrane barriers exist, mediated electron transfer dramatically enhances photocurrent (often by one-2 orders of magnitude). After systematically optimizing the micropillar architecture to enhance both light penetration and cell loading, they achieved a record-high photocurrent of 245 μA cm^–2^, approaching the theoretical maximum, and an external quantum yield of up to 29% under low light intensities (3 mW cm^–2^ and 1 mW cm^–2^, respectively).

#### 6.2.2 Glucose Oxidation Reaction

Coupling the water oxidation reaction to supply electrons and protons for the reductive half-reactions at the photocathode is promising but is often hindered by its inherently sluggish four-electron transfer kinetics, requiring a high overpotential and causing significant energy loss. Replacing the OER with two-electron organic oxidation processes, such as glucose-to-gluconic acid/gluconolactone conversion, provides a kinetically favorable alternative. To develop an efficient biohybrid photoanode for glucose oxidation, Efrati et al. immobilized the glucose oxidase (GOx) with PSI, wired through an Os-complex redox polymer mediator.^37^ The biocatalytic assembly was further immobilized onto an indium tin oxide (ITO) electrode using a chemically bonded quinone-based interfacial monolayer (Figure 22a). Despite PSI normally being utilized on the photocathode, by careful selection of the interfacial redox mediator, they achieved an efficient photodriven glucose oxidation reaction, which was evidenced by a clear correlation between anodic photocurrent and glucose reactant concentration (Figure 22b). This work established a new paradigm for constructing photobioelectrochemical systems with a configuration of PSI/redox polymer/oxidase conjugates. In a related study, Riedel et al. introduced a dual-functional organic polymer, polyaniline, that simultaneously served as a light-harvesting unit and a charge transfer mediator (Figure 22c).^38^ In their biohybrid photoanode, polyaniline facilitated photoinduced electron injection into TiO_2_ while also enabling efficient transfer of photogenerated holes to the immobilized GOx. This dual functionality allowed for a significant simplification of the device architecture. With the designed photoanode, they obtained a glucose-concentration-dependent photocurrent (Figure 22d). It confirmed the effectiveness of the light-driven electron transfer cascade, initiated by enzymatic glucose oxidation and mediated via the polyaniline mediator to the underlying TiO_2_. Compared to flat TiO_2_ electrodes, they found an over 20-fold higher glucose-dependent photocurrent in 3D-TiO_2_ electrodes due to a higher polymer and enzyme loading. Consequently, a maximum photocurrent density of 44.7 ± 6.5 μA cm^–2^ at low potentials was achieved in the presence of glucose. Thus, multifunctional organic materials reduce interfacial resistance and simplify the biohybrid device configuration, improving robustness.

**Figure 22:**
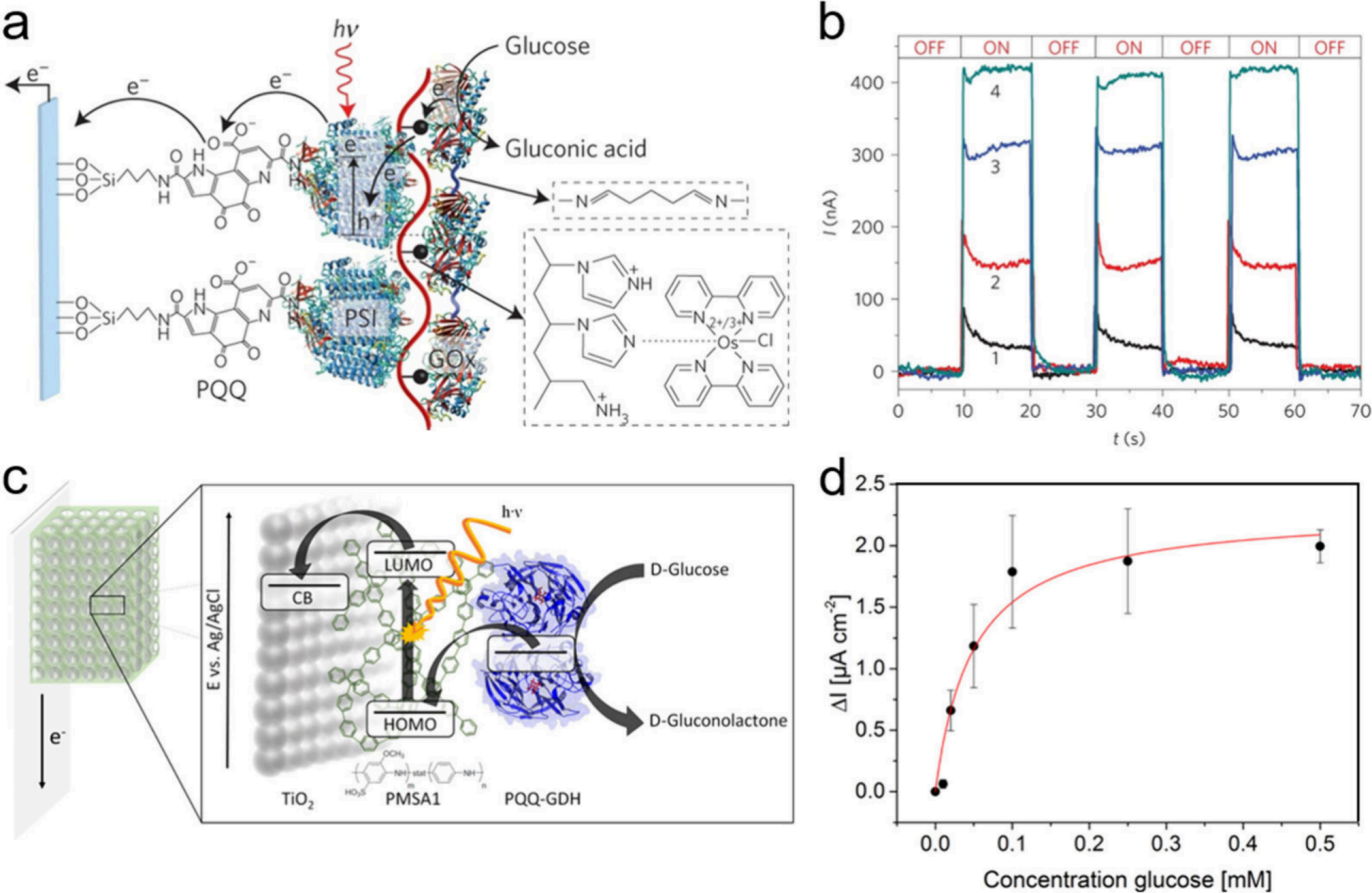
(a) Scheme of biohybrid PEC device architecture of a glucose oxidase immobilized PSI based photoanode for glucose oxidation, wired with a covalent bonding redox mediator, and (b) its photocurrent response under chopping light with a bias of at 0 V vs Ag QRE, 1 to 4 indicates an increased glucose concentration; (c) Glucose oxidase immobilized TiO_2_ based photoanode for glucose oxidation, with polyaniline polymer acting as both light-harvesting unit and electron transfer mediator, and (d) its photocurrent response as the increased concentration of glucose reactant. Panel **a**, **b** reproduced with permission from ref 37. Copyright (2016) Springer Nature Ltd.; Panel **c**, **d** reproduced from ref 38. Copyright (2018) American Chemical Society.

A comparative analysis of Type I-III organic light-harvester-based biohybrid PEC systems reveals several design principles. First, the breadth of light absorption and the intrinsic charge separation and transport ability of the photoactive material are the primary determinants of photocurrent scaling. In this regard, OPV-based (Type III) systems generally outperform PSI- (Type I) and dye-sensitized (Type II) architectures owing to their superior photon capture, high extinction coefficients, and more efficient exciton dissociation and transport within bulk heterojunction structures. Second, interfacial charge recombination emerges as the dominant loss pathway across all configurations. In Type I and Type II systems, in particular, the incorporation of redox polymers or molecular mediators is often essential to accelerate charge extraction and suppress back-electron transfer, thereby improving photocurrent stability and Faradaic efficiency. Third, a key advantage of synthetic organic absorbers over biogenic counterparts lies in their energetic tunability: rational molecular and polymer design enables precise alignment of frontier orbital energies with enzymatic redox potentials, optimizing the thermodynamic driving force while minimizing excess overpotential. Collectively, the current performance hierarchy in terms of photocurrent density follows the trend Type III (mA cm^–2^) > Type II (10^2^ μA cm^–2^) > Type I (μA cm^–2^). Looking toward higher technology readiness levels, future development should target sustained operation at mA cm^–2^ current densities for more than 100 h, Faradaic efficiencies exceeding 90%, higher value-added products, and solar-to-chemical conversion efficiencies above 1%, which together represent meaningful benchmarks for practical biohybrid PEC devices. Additionally, to improve reproducibility and enable meaningful cross-comparison between studies, we recommend that biohybrid and photoelectrochemical investigations report key experimental parameters in a standardized manner. These should include detailed light conditions (spectral distribution, intensity at the sample position, irradiation geometry, and calibration method), headspace composition and control, dissolved O_2_ levels, buffer composition and pH, temperature, and mass transport conditions. In addition, operational stability should be clearly defined (e.g., activity retention over time under continuous illumination), and the number of independent replicates and error analysis should be explicitly reported. Establishing such reporting benchmarks would significantly enhance transparency and comparability across the field.

## 7 Outlook and Future Perspectives

Biohybrid catalytic systems represent a frontier at the intersection of biology, chemistry, and materials science for renewable fuel generation and sustainable chemical production. By merging the precision of the biocatalytic function of enzymes and bacteria with the robustness and versatility of synthetic components, these systems hold the potential to revolutionize catalysis. Over the last few decades, organic molecules have played central roles as photosensitizers, redox mediators, and sacrificial donors, significantly boosting the performance of biohybrid photocatalysts. Despite these advances, major challenges remain. These include (i) ensuring biocompatibility between biological and synthetic components, (ii) enhancing and sustaining efficient electronic communication across bioabiotic interfaces, (iii) achieving balanced coupling between the oxidation and reduction half-reactions to avoid cross-inhibition and/or side reactions, (iv) identifying conditions that push biocatalysts beyond their natural “comfort zones” without compromising viability, and (v) maintaining long-term stability and scalability for the production of high-value products. Addressing these interrelated challenges is essential for translating current laboratory successes into durable, scalable systems capable of practical implementation and societal benefit.

### 7.1 Biocompatibility and Biological Integrity

Biocompatibility remains one of the most decisive yet often underestimated factors in the design of biohybrid photocatalytic systems. The long-term activity and stability of biocatalysts depend critically on the chemical environment established by the photosensitizer, redox mediator, and electron donor. Organic photosensitizers commonly offer intrinsic advantages, their metal-free composition minimizes the risk of metal poisoning in the case of whole-cell catalysis, and their structural tunability facilitates design of the catalyst/photosensitizer interface. Still, similar to their inorganic counterparts, degradation products or excited-state organic components can introduce cytotoxic effects that compromise the cell viability or enzyme integrity. Similarly, the widespread use of traditional redox mediators and sacrificial reagents, such as methyl viologen and triethanolamine, has revealed severe compatibility issues, including the generation of reactive radicals and harmful byproducts. Future progress will rely on a systematic evaluation of component toxicity through colony-forming unit assays,^172^ respiratory activity measurements, assessments of cell membrane integrity,^54^ or utilization of multiomic assays;^173^ coupled with the development of nontoxic, and biocompatible mediators that sustain biological function under illumination. Moreover, tailoring surface charges, hydrophobicities, and functional groups of organic materials to mimic natural interfaces could further minimize stress responses and enhance the longevity of biohybrid assemblies. Advances in time-resolved spectroscopy, electrochemistry, and in situ characterization will be crucial for revealing the mechanistic details of charge transfer and degradation processes. Such interdisciplinary approaches will be key to developing the next generation of light-driven biotechnologies and establishing biohybrid PEC systems. Achieving high photochemical efficiency must therefore go hand in hand with maintaining biological integrity as it will define the practical viability of organic-material-based semiartificial photosynthesis.

### 7.2 Interfacial Electron Transfer and Reaction Coupling

Efficient interfacial charge transfer and reaction coupling remain core engineering and mechanistic bottlenecks. Dispersed biohybrid photocatalytic systems also have challenges in terms of coupling two productive half reactions and ensuring that byproducts caused by one-half reaction do not poison the other half reaction. Immobilizing biohybrid components on electrodes within photoelectrochemical cells offers a promising strategy to separate reduction and oxidation reactions into distinct chambers; however, the interfacial charge transfer from the electrode to the coupled biocatalyst is often insufficient. Organic redox mediators have been proven to be effective in facilitating charge transfer between abiotic and biological units, although as noted above their cytotoxicity to biosystems must be carefully considered. The next generation of mediator-assisted systems should focus on materials that combine high electron mobility, minimal leaching, and strong biocompatibility. The coupling of the half reactions and choice of oxidation half reaction are also important scientific questions to the research community. While H_2_O oxidation is arguably the only electron donor of relevance on global scale, arguments can be made for other oxidation reactions in specific settings, which would also help with the economic analysis of the process.

### 7.3 Beyond the Biological “Comfort Zone”

Even as progress in material design and interface engineering continues, a more fundamental biological challenge persists. Biocatalysts have evolved to operate within narrow windows of temperature, pH, and light exposure that favor survival, rather than maximal productivity. When coupled with artificial components, they often remain in this “comfort zone”, which limits their catalytic performance. Unlocking their full potential will require identifying conditions and regulatory mechanisms that can safely drive biocatalysts beyond their natural operational limits without triggering irreversible stress responses or metabolic collapse. Meeting this challenge will demand both biological and materials innovation: adaptive evolution or synthetic biology could be used to develop more robust strains or enzyme variants, while dynamic material environments might provide tunable conditions that push biological systems to perform under higher light fluxes or redox stress.^200^ Ultimately, achieving this synergy between biological resilience and artificial functionality is what will define the stability and technological relevance of biohybrid systems. The next generation of research must therefore focus on reprogramming biological “comfort zones” to expand the operational boundaries in service of sustainable catalysis for the production of production of complex fuels and chemical feedstocks.^201^

### 7.4 From Stability to Scalability: Toward Industrially Relevant Systems

When biohybrid systems can maintain biocompatibility, efficient charge transfer, and balanced reaction coupling under operational regimes, improving stability and catalytic efficiency at higher active-component concentrations will become the key step toward industrial scale-up. To accelerate progress toward practical implementation, more quantitative performance targets and clearer benchmarking criteria are required for organic-material-based biohybrid systems. As far as we know, the state-of-art photobiocatalytic H_2_ production reported by Yang et al. is around 3.4 mmol g^–1^ h^–1^,^71^ photobiocatalytic CO_2_-to-PHB yield reported by Bai et al. is around 15.4 g L^–1^,^202^ while biohybrid PEC based on NiO photocathode for H_2_ production performance reported by Cheng et al. is around 120 μA cm^–2^.^51^ These values remain far below the realistic applications. The primary bottlenecks preventing such performance include limited exciton dissociation efficiency in purely organic absorbers, interfacial charge recombination by a large exciton binding energy in organic materials, insufficient electronic coupling between abiotic light harvesters and biocatalysts, reactive oxygen species formation under illumination, mediator instability or leaching, and loss of biological activity under redox stress. Moreover, direct comparison across reported systems remains challenging due to substantial variations in light intensity and spectrum, electrode architecture, pH, buffer composition, sacrificial electron donor concentration, applied bias, and performance evaluation methodology. To address this, standardized benchmarking practices should be adopted, including reporting photocatalytic performance normalized to illuminated area under calibrated AM 1.5G conditions, faradaic efficiency over defined time intervals, turnover number and turnover frequency of the biocatalyst, operational lifetime under continuous illumination, and solar-to-chemical conversion efficiency. As for practical and large-scale implementation of biohybrid photoelectrochemical devices, high-performance systems will likely require the synergistic integration of multiple interfacial design strategies rather than reliance on a single electron transfer pathway.^40^ Addressing these challenges in interface design, efficiency, stability, and scalability will open new avenues for transformative applications in sustainable chemistry, renewable energy, and beyond. In particular, developing biohybrid systems capable of converting renewable feedstocks into high-value molecules, such as fine chemicals, and energy-dense molecules, for example sustainable aviation fuels,^200^ or activate nitrogen into fertilizer at near ambient conditions could enable the gradual replacement of current oil and gas refinery outputs, positioning semiartificial systems as key contributors to a circular carbon economy.^203^ Based on current evidence, particularly promising strategies include the integration of broad-absorption conjugated organic semiconductors or high-triplet-yield organic photosensitizers with biocompatible redox polymer wiring layers and robust enzymes or engineered whole-cell catalysts. Such modular architectures combine efficient light harvesting, controlled electron delivery, and improved biological resilience, offering a rational pathway toward scalable and durable semiartificial photosynthetic systems.

## Abbreviations

3D: three-dimensional
AA: l-ascorbic acid
ABTS: 2,2-azinobis (3-ethylbenzothiazolin-6-sulfinic acid) diammonium salt
ABA: poly­(N,N-dimethylamino ethyl methacrylate)-B-poly­(9,9-N-dihyxyl-2,7-fluorene)-B-poly­(N,N-dimethylamino ethyl methacrylate)
ADH: alcohol dehydrogenase
Asp-CDs: aspartic acid-based carbon dots
ATP: adenosine triphosphate
BET: Brunauer–Emmett–Teller
BVMO: Baeyer–Villiger monooxygenase
CA: carbonic anhydrase
CDs: carbon dots
CODH: carbon monoxide dehydrogenase
CO2RR: carbon dioxide reduction reaction
COEs: conjugated oligoelectrolytes
COFs: covalent–organic frameworks
CR: charge recombination
CS: charge separation
DCBQ: 2,6-dichloro-1,4-benzoquinone
DCPIP: 2,6-dichlorophenolindophenol
DQ: diquat
DET: direct electron transfer
DMEN: N,N′-dimethylethylenediamine
DPP: diketopyrrolopyrrole
DTT: dl-dithiothreitol
EDC: 1-ethyl-3-(3-dimethylaminopropyl)­carbodiimide hydrochloride
EDTA: ethylenediaminetetraacetic acid
*E. coli*: *Escherichia coli*
EQE: external quantum efficiency
ET: electron transfer
EY: eosin Y
F8T2: poly­[(9,9-dioctylfluorenyl-2,7-diyl)-co-bithiophene]
FAD: flavin adenine dinucleotide
FaldDH: formaldehyde dehydrogenase
FccA: fumarate reductase
FDH: formate dehydrogenase
FE: faradaic efficiency
FF-THPP: light-harvesting peptide nanotubes synthesized through the self-assembly of diphenylalanine (FF) and tetrahydroxyphenylporphyrin (THPP)
FMN: flavin mononucleotide
FRET: fluorescence resonance energy transfer
GDH: glutamate dehydrogenase
g-CNx or g-C_3_N_4_: graphitic carbon nitride
g-N-CDs: graphitic nitrogen doped carbon dots
GOR: glucose oxidation reaction
HAT: hydrogen atom transfer
HEPES: 2-[4-(2-hydroxyethyl) piperazin-1-yl] ethane-1-sulfonic acid
HER: hydrogen evolution reaction
HOMO: highest occupied molecular orbital
HTCC: hydrothermal carbonation carbon
ISC: intersystem crossing
KHR: ketone hydrogenation reaction
LSV: linear sweep voltammetry
LUMO: lowest unoccupied molecular orbital
MB: methylene blue
MEH-PPV: poly­[2-methoxy-5-(2-ethylhexyloxy)-1,4-phenylenevinylene]
MET: mediated electron transfer
MOPS: 3-(N-morpholino) propanesulfonic acid
MtrAB: outer-membrane-spanning porin·cytochrome complex
MV: methyl viologen
NADH: nicotinamide adenine dinucleotide
NIR: near-infrared
N_pyo_-CDs: nitrogen-doped carbon dots
NPs: nanoparticles
NQ-LPEI: naphthoquinone-grafted linear polyethyleneimine polymer
OER: oxygen evolution reaction
OPSs: organic photosensitizers
OPV: organic photovoltaic
ORR: oxygen reduction reaction
OYEs: Old Yellow Enzymes
P-Os: poly­(1-vinylimidazole-co-allylamine)-Os­(bipy)­2Cl
P2: poly­(N,N-dimethylamino ethyl methacrylate)-B-poly­(9,9-N-dihyxyl-2,7-fluorene)-B-poly­(N,N-dimethylamino ethyl methacrylate)
P3HT: poly­(3-hexylthiophene-2,5-diyl)
PCBM: [6,6]-phenyl-C61-butyric acid methyl ester
PDHA: polydihydroxy aniline
PDI: perylene diimide derivative
Pdots: polymer dots
PEC: photoelectrochemical
PEDOT:PSS: poly­(3,4-ethylenedioxythiophene) polystyrene sulfonate
PFBT: poly­(9,9-dioctylfluorene-alt-benzothiadiazole)
PFP: poly­(fluorene-co-phenylene)
PHB: poly­(3-hydroxybutyrate)
PNPs: polymer nanoparticles
PSI: photosystem I
PSII: photosystem II
RF: riboflavin
RHE: reversible hydrogen electrode
rGO: reduced graphene oxide
RuBisCo: ribulose-1,5-bisphosphate carboxylase/oxygenase
RuP: [Ru­(bpy)­2­{4,4′-(PO_3_H_2_)_2_bpy}]­Br_2_)
*S. oneidensis*: *Shewanella oneidensis*
SED: sacrificial electron donors
SEM: scanning electron microscope
SHE: standard hydrogen electrode
sMNP: organic small molecule nanoparticles
STH: solar-to-hydrogen
TEA: triethylamine
TEOA: triethanolamine
TOF: turnover frequency
TON: turnover number
UV: ultraviolet
γ-PGA: γ-polyglutamic acid
SEM: scanning electron microscope
